# Acidification Enhances Hybrid N_2_O Production Associated with Aquatic Ammonia-Oxidizing Microorganisms

**DOI:** 10.3389/fmicb.2016.02104

**Published:** 2017-01-09

**Authors:** Caitlin H. Frame, Evan Lau, E. Joseph Nolan, Tyler J. Goepfert, Moritz F. Lehmann

**Affiliations:** ^1^Department of Environmental Sciences, University of BaselBasel, Switzerland; ^2^Department of Natural Sciences and Mathematics, West Liberty UniversityWest Liberty, WV, USA; ^3^Helmholtz Center for Ocean Research, GEOMARKiel, Germany

**Keywords:** nitrous oxide, ammonia oxidation, nitrification, acidification, Lake Lugano, isotopomer, 16S rRNA multiplex sequencing, hybrid nitrous oxide

## Abstract

Ammonia-oxidizing microorganisms are an important source of the greenhouse gas nitrous oxide (N_2_O) in aquatic environments. Identifying the impact of pH on N_2_O production by ammonia oxidizers is key to understanding how aquatic greenhouse gas fluxes will respond to naturally occurring pH changes, as well as acidification driven by anthropogenic CO_2_. We assessed N_2_O production rates and formation mechanisms by communities of ammonia-oxidizing bacteria (AOB) and archaea (AOA) in a lake and a marine environment, using incubation-based nitrogen (N) stable isotope tracer methods with ^15^N-labeled ammonium (^15^${\text{NH}}_{4}^{+}$) and nitrite (^15^${\text{NO}}_{2}^{-}$), and also measurements of the natural abundance N and O isotopic composition of dissolved N_2_O. N_2_O production during incubations of water from the shallow hypolimnion of Lake Lugano (Switzerland) was significantly higher when the pH was reduced from 7.54 (untreated pH) to 7.20 (reduced pH), while ammonia oxidation rates were similar between treatments. In all incubations, added ${\text{NH}}_{4}^{+}$ was the source of most of the N incorporated into N_2_O, suggesting that the main N_2_O production pathway involved hydroxylamine (NH_2_OH) and/or ${\text{NO}}_{2}^{-}$ produced by ammonia oxidation during the incubation period. A small but significant amount of N derived from exogenous/added ^15^${\text{NO}}_{2}^{-}$ was also incorporated into N_2_O, but only during the reduced-pH incubations. Mass spectra of this N_2_O revealed that ${\text{NH}}_{4}^{+}$ and ^15^${\text{NO}}_{2}^{-}$ each contributed N equally to N_2_O by a “hybrid-N_2_O” mechanism consistent with a reaction between NH_2_OH and ${\text{NO}}_{2}^{-}$, or compounds derived from these two molecules. Nitrifier denitrification was not an important source of N_2_O. Isotopomeric N_2_O analyses in Lake Lugano were consistent with incubation results, as ^15^N enrichment of the internal N vs. external N atoms produced site preferences (25.0–34.4‰) consistent with NH_2_OH-dependent hybrid-N_2_O production. Hybrid-N_2_O formation was also observed during incubations of seawater from coastal Namibia with ^15^${\text{NH}}_{4}^{+}$ and ${\text{NO}}_{2}^{-}$. However, the site preference of dissolved N_2_O here was low (4.9‰), indicating that another mechanism, not captured during the incubations, was important. Multiplex sequencing of 16S rRNA revealed distinct ammonia oxidizer communities: AOB dominated numerically in Lake Lugano, and AOA dominated in the seawater. Potential for hybrid N_2_O formation exists among both communities, and at least in AOB-dominated environments, acidification may accelerate this mechanism.

## Introduction

Ammonia oxidizing bacteria (AOB) and archaea (AOA) are a source of the greenhouse gas nitrous oxide (N_2_O) (Goreau et al., 1980; Santoro et al., 2011; Löscher et al., 2012) in soils and aquatic environments. The rate at which these microorganisms produce N_2_O depends on the rate at which they carry out chemosynthetic reactions that oxidize ammonia (NH_3_) to nitrite (${\text{NO}}_{2}^{-}$). However, other environmental factors can enhance their N_2_O production rate, such as reduced oxygen (O_2_) concentrations (Goreau et al., 1980; Löscher et al., 2012), higher ${\text{NO}}_{2}^{-}$ concentrations, and higher densities of ammonia-oxidizing cells (Frame and Casciotti, 2010). In soils, pH is another factor that influences N_2_O production, with acidic soils generally producing more N_2_O than alkaline soils (Martikainen, 1985). Certain lakes and marine environments also experience pH decreases, which may occur naturally as a result of rapid respiration of organic carbon to carbon dioxide (CO_2_), or by the dissolution of acid-forming gases (e.g., CO_2_, sulfur dioxide, and nitrogen oxides) produced by human activities.

There are several ways in which reducing the pH of aquatic environments (i.e., acidification) may affect the rate of N_2_O production by ammonia oxidizers. Some evidence suggests that acidification will cause ammonia oxidation rates to decline. Specifically, the ammonia monooxygenase enzyme (AMO), which catalyzes conversion of NH_3_ to the intermediate hydroxylamine (NH_2_OH), is thought to act on the free base form of the substrate (NH_3_), rather than the protonated form, ammonium (${\text{NH}}_{4}^{+}$) (Suzuki et al., 1974; Stein et al., 1997). In the pH range of many natural aquatic systems (pH 6–8) ${\text{NH}}_{4}^{+}$/NH_3_ is mostly present as ${\text{NH}}_{4}^{+}$ (pKa = 9.25 at 25°C). Any acidification will further reduce the fraction of ${\text{NH}}_{4}^{+}$/NH_3_ that is present as NH_3_, and thus reduce the substrate concentration for ammonia oxidizers.

The net effect of ammonia oxidation is also acidifying, releasing protons (H^+^) to the surrounding environment:

(1) $$2{\text{NH}}_{4}^{+}+3{\text{O}}_{2}\to 2{\text{NO}}_{2}^{-}+4{\text{H}}^{+}+2{\text{H}}_{2}\text{O},$$

so that, for example, AOB batch cultures that are actively consuming NH_3_ are normally exposed to pH decreases as they grow. In these cultures, once the pH drops below ~6.5, further ammonia oxidation is inhibited (Allison and Prosser, 1993; Jiang and Bakken, 1999b). However, the reason for this may not be decreased substrate availability, since decreases in the activity of AOB are not necessarily correlated with reductions in the NH_3_ concentration (Jiang and Bakken, 1999a). It is more likely that inhibition is caused by other factors, such as toxic buildup of nitrous acid (HNO_2_), nitric oxide (NO), and nitrogen dioxide (NO_2_) under acidic conditions (Schmidt and Bock, 1997; Stein and Arp, 1998; Schmidt et al., 2002; Udert et al., 2003; Park and Bae, 2009). Recent environmental studies suggest that ammonia oxidation rates may not have a single relationship to pH. For example, ammonia oxidation rates in the open ocean are inhibited by acidification (from pH 8.1–8.2 down to pH 7.6–7.8; Beman et al., 2011; Rees et al., 2016), whereas sedimentary ammonia oxidation rates do not seem to be sensitive to acidification (from pH 8 down to 6; Kitidis et al., 2011).

AOA may not be subject to the same growth inhibition as AOB at lower pH ranges. For example, an obligately acidophilic AOA with an optimum pH range of 4–5 was discovered in acidic soil (Lehtovirta-Morley et al., 2011), and in soil pH manipulation experiments, archaeal *amoA* transcript abundances outnumbered those of AOB in acidic soils (Nicol et al., 2008), suggesting that AOA may outcompete AOB in acidic environments. Marine AOA, which are generally regarded as more important than AOB to ammonia oxidation in the ocean (Wuchter et al., 2006), may also be more tolerant of acidic conditions. For example, certain marine AOA strains are capable of maintaining near-maximal growth rates down to a pH of 5.9 (Qin et al., 2014), perhaps because they express ${\text{NH}}_{4}^{+}$-transport proteins that actively transport ${\text{NH}}_{4}^{+}$ into AOA cells, thus supplying AMO with NH_3_ under acidic conditions (Lehtovirta-Morley et al., 2011, 2016).

Unlike ${\text{NO}}_{2}^{-}$, N_2_O is not the major nitrogenous product of ammonia oxidation, and it is not known to what degree the reactions that produce N_2_O are convolved with the main energy-harnessing reactions of ammonia oxidizers (i.e., NH_3_ oxidation to NH_2_OH and then to ${\text{NO}}_{2}^{-}$). This means that the impact of pH on the N_2_O production rate may be decoupled from its impact on the ammonia oxidation rate. That is, even if acidification decreases the ammonia oxidation rate, the N_2_O production rate may not necessarily also decrease proportionally. In fact, many of the reactive nitrogen oxides produced during ammonia oxidation undergo N_2_O-forming reactions over relevant timescales, with or without enzyme catalysis, and with their own pH-dependencies.

**Figure 1:**
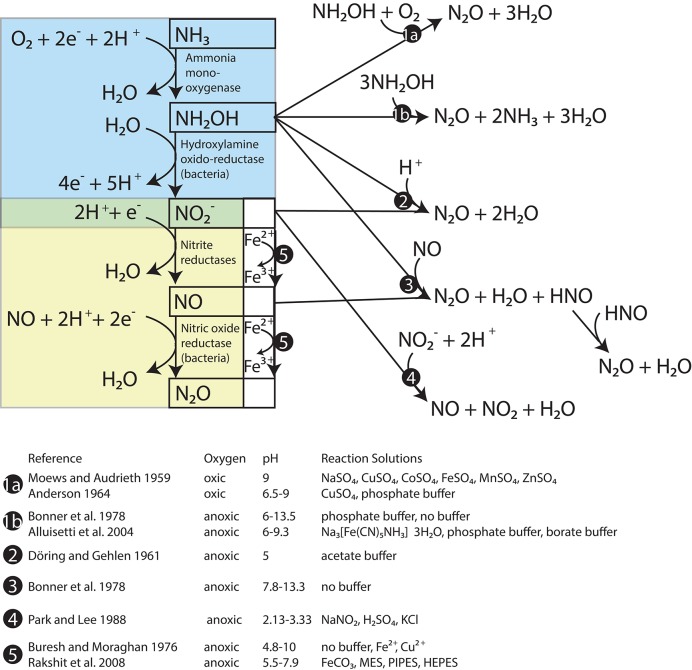
**Reactions between products of ammonia oxidation that produce N_2_0**. The steps of ammonia oxidation are in the blue box and the steps of nitrifier denitrification are in the yellow box. Known abiotic pathways to N_2_0 formation are located outside these boxes.

One of these nitrogen oxides is NH_2_OH, which is the enzymatic product of NH_3_ oxidation by AMO in both AOB and AOA (Figure 1, blue box; Hofman and Lees, 1953; Vajrala et al., 2013). Although most NH_2_OH is converted to ${\text{NO}}_{2}^{-}$ during active ammonia oxidation, NH_2_OH is also subject to abiotic autoxidation (Figure 1, pathway 1a) and disproportionation reactions (Figure 1, pathway 1b) that produce N_2_O as well as nitrogen (N_2_), nitric oxide (NO), and NH_3_/${\text{NH}}_{4}^{+}$. N_2_O yields during these reactions vary with alkalinity (Bonner et al., 1978), redox conditions (Moews and Audrieth, 1959; Pacheco et al., 2011), and the presence of certain transition metals (Anderson, 1964; Alluisetti et al., 2004). NH_2_OH may also react with ${\text{NO}}_{2}^{-}$/HNO_2_ to produce N_2_O (Figure 1, pathway 2). This reaction occurs abiotically at a rate that accelerates as pH decreases (Döring and Gehlen, 1961; Bonner et al., 1983). It can also be catalyzed by the copper- and iron-containing ${\text{NO}}_{2}^{-}$ reductases of certain denitrifying bacteria (Iwasaki et al., 1963; Kim and Hollocher, 1984), as well as soluble enzyme extracts of AOB that have an acidic optimum pH (Hooper, 1968). A reaction such as pathway 2 could explain the “hybrid” N_2_O production observed in AOA cultures, where one ${\text{NO}}_{2}^{-}$-derived N atom and one NH_3_-derived N atom (e.g., from NH_2_OH) were combined into the same N_2_O molecule (Stieglmeier et al., 2014b). Furthermore, Harper et al. (2015) found that this hybrid reaction between NH_2_OH and ${\text{NO}}_{2}^{-}$ was responsible for most of the N_2_O produced by activated sludge during bioreactor experiments. NH_2_OH may also react abiotically with NO to form N_2_O and N_2_ in proportions that are pH-dependent (Figure 1, pathway 3; Bonner et al., 1978). In terms of tracing the source compounds contributing N to N_2_O, NO can be derived abiotically from HNO_2_ through a disproportionation reaction (Figure 1, pathway 4; Park and Lee, 1988), and the reaction of HNO_2_-derived NO with NH_2_OH could also produce a hybrid type N_2_O. However, abiotic disproportionation HNO_2_ tends to be most important only in very acidic environments (pKa HNO_2_ = 2.8; Riordan et al., 2005).

Reduction of ${\text{NO}}_{3}^{-}$ and ${\text{NO}}_{2}^{-}$ by trace metal ions (Buresh and Moraghan, 1976) and metal-containing minerals (e.g., Rakshit et al., 2008) is known as chemodenitrification (Figure 1, pathway 5). In this process, reduced metal species, particularly Fe^2+^ (and possibly also Mn^2+^) are oxidized, and NO, N_2_O, and N_2_ are produced (Picardal, 2012). This pathway has a recognized importance in soils (Zhu-Barker et al., 2015), but is less studied in seawater and eutrophic lake water, which typically have much lower metal concentrations (Morel et al., 2003) than soil. Reducing sediments along productive continental margins may support significant rates of chemodenitrification (Scholz et al., 2016).

Enzymatic reduction of ${\text{NO}}_{2}^{-}$ to NO and N_2_O in AOB is known as nitrifier denitrification (Figure 1, yellow box). This pathway produces N_2_O whose N atoms are both derived from ${\text{NO}}_{2}^{-}$.^1^ The existence of this pathway in AOB was confirmed in cultures of *Nitrosomonas europaea* by the production of N_2_O with a molecular mass of 46 (^46^N_2_O = ^15^N^15^N^16^O) after tracer additions of ^15^${\text{NO}}_{2}^{-}$ (Poth and Focht, 1985). However, in similar experiments with AOA cultures, Stieglmeier et al. (2014b) observed no ^46^N_2_O production, even at low O_2_ concentrations that are thought to stimulate nitrifier denitrification in AOB (Goreau et al., 1980). Similarly, microrespirometry measurements of *Nitrososphaera viennensis* cultures indicate that this AOA does not reduce ${\text{NO}}_{2}^{-}$ to N_2_O (Kozlowski et al., 2016).

In AOB, NO produced by nitrite reduction is converted to N_2_O by a membrane-bound NO reductase (NOR) that reduces 2NO to N_2_O (Figure 1, yellow box; Beaumont et al., 2004b; Kozlowski et al., 2014). In some denitrifiers, the NOR homolog that carries out the same reduction of NO to N_2_O, has a neutral to acidic pH optimum (5–7.6; Hoglen and Hollocher, 1989) raising the possibility that this step in nitrifier denitrification also has a slightly acidic pH optimum. Among AOA, however, no homologs for the catalytic subunit of bacterial NOR (*norB*) have been found in any sequenced genomes to date (Santoro et al., 2015), confirming tests of AOA cultures that indicate that nitrifier denitrification does not occur in these organisms (Stieglmeier et al., 2014b; Kozlowski et al., 2016).

NO is a precursor of N_2_O during bacterial nitrifier denitrification, but its production and consumption may be involved in other processes in ammonia oxidizers. For example, NO is an intermediate in the catalytic cycle of hydroxylamine oxidoreductase (HAO) (Cabail and Pacheco, 2003), an enzyme that oxidizes NH_2_OH to ${\text{NO}}_{2}^{-}$ in AOB (Figure 1, blue box). NO production is also required for AOA to carry out their ammonia oxidation cycle (Shen et al., 2013; Martens-Habbena et al., 2015; Kozlowski et al., 2016), though no HAO homologs have been identified among AOA (Hallam et al., 2006; Walker et al., 2010).

Field studies assessing the importance of N_2_O production pathways in aquatic environments have relied on two approaches to date: (1) ^15^N tracer incubation studies that track the incorporation of N derived from ^15^N-labeled precursor molecules, and (2) dissolved N_2_O measurements of the bulk O and N stable isotopic composition as well as the intramolecular distribution of ^15^N and ^14^N between the internal and external N atoms of the linear, asymmetrical N_2_O molecule (known as site preference; SP = δ^15^N^internal^-δ^15^N^external^; Toyoda and Yoshida, 1999). The SP signature can be useful for distinguishing N_2_O production pathways because it is often (but not always) independent of the isotopic composition of the starting compounds (Yang et al., 2014). Using the first approach, Nicholls et al. (2007) and Trimmer et al. (2016) may have observed hybrid N_2_O formation by an ammonia oxidizer community immediately above the oxygen minimum zone (OMZ) of the Arabian Sea and in the Eastern Tropical North Pacific, respectively, where they observed ^45^N_2_O but not ^46^N_2_O production during tracer incubations with ^15^${\text{NH}}_{4}^{+}$ and ${\text{NO}}_{2}^{-}$ with a natural abundance (NA) isotopic composition. In studies using the second approach, profiles of the SP of N_2_O have been used to distinguish N_2_O produced by NH_2_OH-dependent pathway(s), which have a distinctly higher SP (~34‰; e.g., Sutka et al., 2006; Heil et al., 2014; Frame and Casciotti, 2010) than N_2_O that is formed during denitrification and nitrifier denitrification, which has a much lower SP (0 to −5‰; Toyoda et al., 2005; Sutka et al., 2006; Yamazaki et al., 2014).

Here we have used profiles of dissolved inorganic N concentrations (${\text{NH}}_{4}^{+}$, ${\text{NO}}_{3}^{-}$, ${\text{NO}}_{2}^{-}$, and N_2_O) and the natural abundance isotopic composition of ${\text{NO}}_{3}^{-}$, ${\text{NO}}_{2}^{-}$, and N_2_O to locate depths where ammonia oxidation and/or N_2_O production are important in the water columns of Lake Lugano, a human-impacted lake in southern Switzerland, and the marine upwelling zone off the Namibian coast of southwestern Africa. N_2_O isotope and site preference profiles were used to identify the likely pathways of N_2_O production and the involved substrates/intermediates in the two environments. Short (24–30 h) incubations with ^15^N-tracers (^15^${\text{NH}}_{4}^{+}$ and ^15^${\text{NO}}_{2}^{-}$) at targeted depths revealed that hybrid N_2_O formation occurred in both the shallow hypolimnion of Lake Lugano, as well as in water from the Namibian upwelling zone. Furthermore, N_2_O yields produced during incubations of Lake Lugano water were significantly higher when the pH was reduced experimentally. The isotopic composition of the N_2_O that was produced indicated that the increase was due, at least in part, to enhanced incorporation of N derived from exogenous ${\text{NO}}_{2}^{-}$. Multiplex sequencing of microbial 16S rRNA from the incubation locations indicated that AOB numerically dominated the ammonia-oxidizing community in Lake Lugano whereas AOA dominated in the Namibian Upwelling zone. The lines of evidence presented here suggest that there is potential, at least over the short term, for acidification to enhance hybrid N_2_O formation in aquatic environments.

## Methods

### Sampling

Lake Lugano is separated into a permanently stratified northern basin and a monomictic southern basin. This study focuses on the 95 m-deep southern basin. Water samples and incubation water were collected with a 5L Niskin bottle at the Figino Station (45.95°N, 8.90°E) during a sampling campaign on November 5, 2013. Profiles of dissolved O_2_, temperature, salinity, and pH were collected by a conductivity, temperature, and depth sensor (CTD). O_2_ profiles were calibrated by Winkler titration. Water from the Namibian Upwelling zone was collected by hydrocast with a 10L-Niskin bottle rosette at station 89 (20.65°S, 10.95°E) on January 28, 2014 during the NamUFil cruise of the R/V *Meteor*.

### Geochemical profiles

Water samples for ${\text{NH}}_{4}^{+}$ and ${\text{NO}}_{3}^{-}$ concentrations, as well as ${\text{NO}}_{3}^{-}$ isotope measurements were immediately filtered through 0.22 μm-pore sterivex filters (Millipore) and then frozen within 2 h of sampling. ${\text{NH}}_{4}^{+}$ concentrations were measured fluorometrically (Holmes et al., 1999). ${\text{NO}}_{2}^{-}$ concentrations were determined by converting ${\text{NO}}_{2}^{-}$ present in 10 ml of sample water to N_2_O by azide reduction (McIlvin and Altabet, 2005) and then quantifying the amount of N_2_O in each sample by gas chromatography-isotope ratio mass spectrometry (GC-IRMS, see below). ${\text{NO}}_{2}^{-}$ concentration standards were prepared in 10 ml of distilled water and in lake-water or seawater, and were analyzed by GC-IRMS along with the samples. For ${\text{NO}}_{3}^{-}$ concentration and isotopic measurements, sulfamic acid was used to remove ${\text{NO}}_{2}^{-}$ prior to analysis (Granger and Sigman, 2009). ${\text{NO}}_{3}^{-}$ concentrations were measured by reduction to NO with Vanadium (III) and chemiluminescence detection (Braman and Hendrix, 1989). Nitrate N and O isotope measurements of duplicate samples were performed by conversion of ${\text{NO}}_{3}^{-}$ to N_2_O using the denitrifier method (Sigman et al., 2001; Casciotti et al., 2002) and subsequent purification and analysis of this N_2_O with a modified purge-and-trap gas bench GC-IRMS (Thermo Finnigan DeltaV Plus) system. Isotopic calibration was performed by concurrent analysis of ${\text{NO}}_{3}^{-}$ isotope standards USGS 32, USGS 34, and USGS 35 (Casciotti et al., 2008). N and O isotopic data are reported on the permil (‰) scale referenced to air N_2_ and Vienna Standard Mean Ocean Water (VSMOW), respectively (δ^15^N = ([^15^N]/[^14^N])_sample_ / [^15^N]/[^14^N]_air_N2_ − 1) × 1000‰ and δ^18^O = ([^18^O]/[^16^O]_sample_ / [^18^O]/[^16^O]_VSMOW_ − 1) × 1000‰).

Samples for N_2_O concentration and isotope analyses were taken by overfilling 160 ml glass sample bottles twice from the bottom through a plastic hose connected to the Niskin outlet. The Lake Lugano N_2_O samples were preserved by adding 100 μl of saturated mercuric chloride solution (HgCl_2_) after a headspace was added by pipetting 1 ml of water off the top of each bottle. Each bottle was then sealed with a butyl rubber septum (VWR, 5483369) and aluminum crimps (CS Chromatographie, 300219). The marine samples were preserved by adding 5 ml of 10 M sodium hydroxide (NaOH) to the bottom of each bottle with a syringe (Mengis et al., 1997), pipetting 1 ml of water off the top for headspace, sealing with butyl septa and aluminum crimps, and then shaking vigorously to distribute the NaOH. Lake Lugano N_2_O samples were analyzed within 1 week of collection. Marine samples were analyzed within 3 months of collection. The total N_2_O in each sample was purged with carrier helium directly into a customized purge-and-trap system (McIlvin and Casciotti, 2010) and analyzed by continuous-flow GC-IRMS. Duplicate N_2_O samples at each depth were collected for the Lake Lugano profile and one sample from each depth was analyzed for the Namibian Upwelling profile. N_2_O isotope ratios were referenced to N_2_O injected from a reference N_2_O tank (≥99.9986%, Messer) calibrated on the Tokyo Institute of Technology scale (Mohn et al., 2012) for bulk and site-specific isotopic composition by J. Mohn (EMPA, Switzerland). Ratios of m/z 45/44, 46/44, and 31/30 signals were converted to δ^15^N-N_2_O (referenced to N_2_AIR_), δ^18^O-N_2_O (referenced to Vienna Standard Mean Ocean Water), and site-specific δ^15^N^α^ and δ^15^N^β^-N_2_O according to Frame and Casciotti (2010), with an additional two-point correction (Mohn et al., 2014) using measurements of two isotopic mixtures of N_2_O in synthetic air (CA-06261 and 53504; kindly provided by J. Mohn). N_2_O concentrations were calculated by converting the N_2_O sample peak areas measured by GC-IRMS to N_2_O standards prepared by converting ${\text{NO}}_{3}^{-}$ to a known quantity of N_2_O by the denitrifier method (McIlvin and Casciotti, 2010). At each depth, temperature and salinity data were used to calculate the N_2_O concentrations at equilibrium with the atmosphere according to Weiss and Price (1980), based on atmospheric partial pressures reported by NOAA ESRL Global Monitoring Division (http://esrl.noaa.gov/gmd/). The saturation disequilibrium (ΔN_2_O) was calculated as the difference between the measured N_2_O concentration and the atmospheric equilibrium concentration, with positive values corresponding to oversaturation.

### Incubations

A list of all incubation treatments is provided in Table 1. Water for the Lake Lugano incubations that was collected at 17 m depth was poured into opaque 10 L HDPE canisters (Huber, 15.0250.03), stored in the dark for ~5 h during transport back to the laboratory, and then amended with ^15^N-labeled incubation reagents. Water for the seawater incubations was drawn from 200 m depth and immediately mixed with the ^15^N-labeled substrates inside 3.4 L LDPE drinking water containers (Campmor, 81027). A dilution (1:2) of 30% hydrochloric acid (Fluka TraceSelect, 96208) with milliQ water was added to water for reduced-pH incubations of Lake Lugano water and mixed immediately before ${\text{NH}}_{4}^{+}$ and ${\text{NO}}_{2}^{-}$ substrates were added. Tracer ^15^NH_4_Cl (98.5%) and Na^15^NO_2_ (99.2%) purchased from Cambridge Isotope Laboratories (NLM-658 and NLM-467) were paired, respectively, with NaNO_2_ and NH_4_Cl with natural abundance (NA) isotopic compositions, so that each incubation received either 1 μM ^15^${\text{NH}}_{4}^{+}$ + 1 μM NA ${\text{NO}}_{2}^{-}$ or 1 μM NA ${\text{NH}}_{4}^{+}$ + 1 μM ^15^${\text{NO}}_{2}^{-}$. For each set of experimental conditions (Table 1), 10 acid-washed 160 ml glass incubation bottles (Wheaton, 223748) were rinsed with milliQ and lake or sea water, and then filled with 120 ml of incubation water and closed with gray fluorobutyl PTFE-lined septa (National Scientific, C4020-36AP) and aluminum crimps. Headspace O_2_ concentrations of the reduced-O_2_ incubations were adjusted by displacement with either high purity (99.999%) helium for the Lake Lugano incubations, or N_2_ for the Namibian Upwelling incubations. O_2_ in the headspace was quantified using a gas chromatograph with an electron-capture detector (SRI 8610C), and O_2_ concentrations were calculated according to Weiss and Price (1980). During the Lake Lugano incubations, one bottle was sacrificed at the beginning of the incubation, and three bottles each were sacrificed after ~5, 17, or 30 h. During the Namibian Upwelling incubations, one bottle for each set of experimental conditions was sacrificed at the beginning of each incubation, and three bottles were sacrificed after ~12 and ~24 h. Bottles were incubated in the dark at 20–21°C for both experiments.

**Table 1:** **Summary of experimental conditions for the Lake Lugano and Namibian Upwelling experiments**.

**Location**	**Name**	**pH**	**[O_2_] μM**	**Tracers**	**Timepoints (hours)**
Lake Lugano	control-pH, control-O_2_	7.54±0.02	290±14	1) 1 μM ^15^${\text{NH}}_{4}^{+}$ + 1 μM NA NO_2_− 2) 1 μM NA ${\text{NH}}_{4}^{+}$ + 1 μM ^15^NO_2_−	0, 5, 17, 30
Lake Lugano	control-pH, reduced-O_2_	7.54±0.02	70±10	1) 1 μM ^15^${\text{NH}}_{4}^{+}$ + 1 μM NA NO_2_− 2) 1 μM NA ${\text{NH}}_{4}^{+}$ + 1 μM ^15^NO_2_−	0, 5, 17, 30
Lake Lugano	Reduced-pH, control-O_2_	7.20±0.02	290±14	1) 1 μM ^15^${\text{NH}}_{4}^{+}$ + 1 μM NA NO_2_− 2) 1 μM NA ${\text{NH}}_{4}^{+}$ + 1 μM ^15^NO_2_−	0, 5, 17, 30
Lake Lugano	reduced-pH, reduced-O_2_	7.20±0.02	70±9	1) 1 μM ^15^${\text{NH}}_{4}^{+}$ + 1 μM NA NO_2_− 2) 1 μM NA ${\text{NH}}_{4}^{+}$ + 1 μM ^15^NO_2_−	0, 5, 17, 30
Namibian upwelling	control-O_2_	–	220±12	1) 1 μM ^15^${\text{NH}}_{4}^{+}$ + 1 μM NA NO_2_− 2) 1 μM NA ${\text{NH}}_{4}^{+}$ + 1 μM ^15^NO_2_−	0, 24
Namibian upwelling	reduced-O_2_	–	50±10	1) 1 μM ^15^${\text{NH}}_{4}^{+}$ + 1 μM NA NO_2_− 2) 1 μM NA ${\text{NH}}_{4}^{+}$ + 1 μM ^15^NO_2_−	0, 24
Namibian upwelling	reduced-O_2_	–	20±10	1) 1 μM ^15^${\text{NH}}_{4}^{+}$ + 1 μM NA NO_2_− 2) 1 μM NA ${\text{NH}}_{4}^{+}$ + 1 μM ^15^NO_2_−	0, 24

Immediately before an incubation bottle was sacrificed, 40 ml of liquid was withdrawn by syringe for colorimetric pH measurements with phenol red (Robert-Baldo et al., 1985) or frozen at −80°C and then stored at −20°C prior to measurements of concentration and dissolved N isotope composition. Seawater incubation samples were also filtered through polycarbonate membrane filters (Whatman nuclepore) with 0.22 μm pores before freezing. ${\text{NH}}_{4}^{+}$ and ${\text{NO}}_{3}^{-}$ concentration measurements were made as described above. The initial ^15^N atom fraction (^15^F = [^15^N]/[^15^N + ^14^N]) of ${\text{NH}}_{4}^{+}$ was calculated at the beginning of the incubation using the added tracer concentration and the ambient concentration of ${\text{NH}}_{4}^{+}$ before tracer addition, and assuming that the δ^15^N of ambient ${\text{NH}}_{4}^{+}$ was 0‰. The ^15^F of ${\text{NH}}_{4}^{+}$ was not measured during the incubations. The ^15^F of ${\text{NO}}_{2}^{-}$ during the incubations was measured using the azide reduction/GC-IRMS method (McIlvin and Altabet, 2005), and the ^15^F of ${\text{NO}}_{3}^{-}$ was measured using the denitrifier method (Sigman et al., 2001) after removal of ${\text{NO}}_{2}^{-}$ with sulfamic acid. ${\text{NO}}_{x}^{-}$-derived N_2_O with a ^15^N composition <15% was analyzed using the IRMS manufacturer's resistor (3 × 10^10^ Ω) and capacitor pairing on the m/z 45 Faraday cup. For more ^15^N-enriched N_2_O, the resistance on the m/z 45 cup was reduced to 3 × 10^8^. Internal isotope standards for ^15^F_*NO*2−_ and ^15^F_*NO*3−_ were prepared in triplicate by mixing NA NaNO_2_ or KNO_3_ of known δ^15^N values with either 99.2% Na^15^NO_2_ or 98.5% Na^15^NO_3_ (Cambridge Isotope Laboratories, NLM-157). The total N_2_O in each incubation bottle was analyzed for concentration and m/z ion ratios 45/44 and 46/44 as described for natural abundance stable isotope measurements of water column N_2_O samples. Additional information about converting IRMS measurements to ^45^N_2_O and ^46^N_2_O production rates is provided in the Supplementary Material S.1.

### DNA extraction

Water from the incubation depth was filtered through a single 0.22 μm-pore size 47 mm polycarbonate Nuclepore filter (Whatman). The volume of water filtered from the Namibian Upwelling was 4 L and from the Lake Lugano was 0.3 L. Filters were frozen immediately and stored at ≤ −80°C until extraction. DNA from either environment was extracted using the FastDNA spin kit for soil (MP Biomedicals).

### Illumina 16S rRNA library generation

The polymerase chain reaction (PCR) was used to amplify the V4 region of the 16S ribosomal RNA (rRNA) gene of prokaryotes using universal 16S rRNA V4 primers F515 (5′- GTGCCAGCMGCCGCGGTAA -3′) and R806 (5′-GGACTACHVGGGTWTCTAAT-3′; Caporaso et al., 2011). Forward and reverse primers were barcoded and appended with Illumina-specific adapters (Kozich et al., 2013). PCR amplifications were carried out using BioReagents™ exACTGene™ Complete PCR Kit and Core Reagent Sets (Thermo Fisher Scientific) for 30 cycles (Caporaso et al., 2012). Agarose gel electrophoresis was used to separate PCR products of the correct size that were then band-excised and recovered using a QIAquick gel extraction kit (Qiagen). For each library, triplicate PCR products were combined and quantified with a Qubit fluorometric assay (ThermoFisher Scientific) and pooled at equimolar ratios. The final pool was analyzed on an Agilent 2100 Bioanalyzer System (Agilent Technologies) using a High Sensitivity DNA chip to determine the average size of the amplicon pool. Quantitative PCR was performed on the pool using a Biorad IQ5 real time thermocycler (Bio-Rad Laboratories) and Illumina quantification standards (KAPA Biosystems). The samples were sequenced on an Illumina MiSeq (http://www.illumina.com/systems/miseq.ilmn) using 201 nucleotide paired-end multiplex sequencing with 5% PhiX spiked into the run. The library was sequenced on a single flow cell using V2 sequencing reagents, generating paired reads of ~400 bp, with ~150 bp overlap between forward and reverse reads.

### Bioinformatic analyses

Raw 16S rRNA sequence data were initially processed in Basespace using Illumina's metagenomic pipeline (https://basespace.illumina.com/home/index). Merging the paired reads and further analyses were carried out using Axiome with installed PANDAseq and the Quantitative Insights Into Microbial Ecology (QIIME v.1.8.0) software pipeline, including taxaplot for overall bacterial diversity, and calculations of Chao1 to estimate taxon richness and rarefaction curves to calculate species richness for a given number of individual sequences sampled (Caporaso et al., 2010; Masella et al., 2012; Lynch et al., 2013). On PANDAseq, the minimum overlap length was set at the threshold of 0.9. The read length maximum for all sequences was 253 bp. Since the V4 region of the 16S rRNA gene is conserved, reads were removed from further analysis if at least one of the following criteria was met: reads were ≥4 bp shorter than the maximum mentioned above, the number of ambiguous bases was ≥1, homopolymers with >4 bp were present, or sequences did not match any sequences in the database by more than 97% based on the percent coverage in BLAST. The clustering of the sequences into operational taxonomic units (OTUs) was initially performed using UCLUST (Edgar, 2010; Edgar et al., 2011) with a cutoff value of 97% sequence identity. The taxonomic identity of a representative sequence from each cluster was classified using the RDP classifier (Wang et al., 2007) and Greengenes datafiles compiled in October 2012 (downloaded from: http://greengenes.lbl.gov/), which includes chimera screening based on 16S rRNA gene records from GenBank (DeSantis et al., 2006). The confidence threshold was set at the default cutoff value of 80% in order to retrieve potential sequences from the nitrifying taxa Thaumarchaeota and Nitrosomonadaceae.

### Phylogenetic analyses

Phylogenetic trees were constructed using MEGA 7 (Kumar et al., 2016). The 16S rRNA gene sequences of representative AOA and AOB were downloaded from GenBank (Benson et al., 2015) for all phylogenetic analyses. Gene sequences were aligned using the MUSCLE algorithm in MEGA 7 and manually inspected. The final alignments consisted of 253 characters, comprising 481 and 108 taxa for sequences related to AOA and AOB, respectively. Phylogenetic reconstruction was implemented using Maximum Parsimony (MP) and Maximum Likelihood (ML). MP was implemented with complete deletion if gaps were present, and tree-bisection-reconnection (TBR) utilized for tree generation. The resulting trees were obtained via random stepwise addition of sequences, at MP search level 1, with 25 initial trees generated from a heuristic search. ML was implemented using Tamura-Nei model of nucleotide substitution rates, with tree inference based on Nearest-Neighbor-Interchange (NNI). Statistical support for MP and ML trees were obtained from 1000 bootstrap replicates under the same initial settings (only bootstrap values >50% are reported). Pairwise base comparisons of OTUs and their closest relatives were determined using BLAST (Altschul et al., 1990) and reported as % identity values.

### Statistical and ecological analyses

The abundances (frequencies) of ammonia oxidizer OTUs in the Namibian Upwelling and Lake Lugano samples were counted using a program written in Python (v.2.7) (https://www.python.org/download/releases/2.7) to search for each OTU that was verified by the above phylogenetic analyses (Lau et al., 2015). The Shannon-Weiner Index and Pielou Evenness were calculated using the BiodiversityR package (Kindt and Coe, 2005) in R version 3.2.2 (http://www.R-project.org/). All partial 16S rRNA gene sequence data are available through the European Bioinformatics Institute (EBI) with project accession number PRJEB11492. The nucleotide sequences for the partial 16S rRNA genes have been deposited in EBI under individual accession numbers (LN908279-LN908785).

## Results

### Geochemical profiles

#### Lake lugano

**Figure 2:**
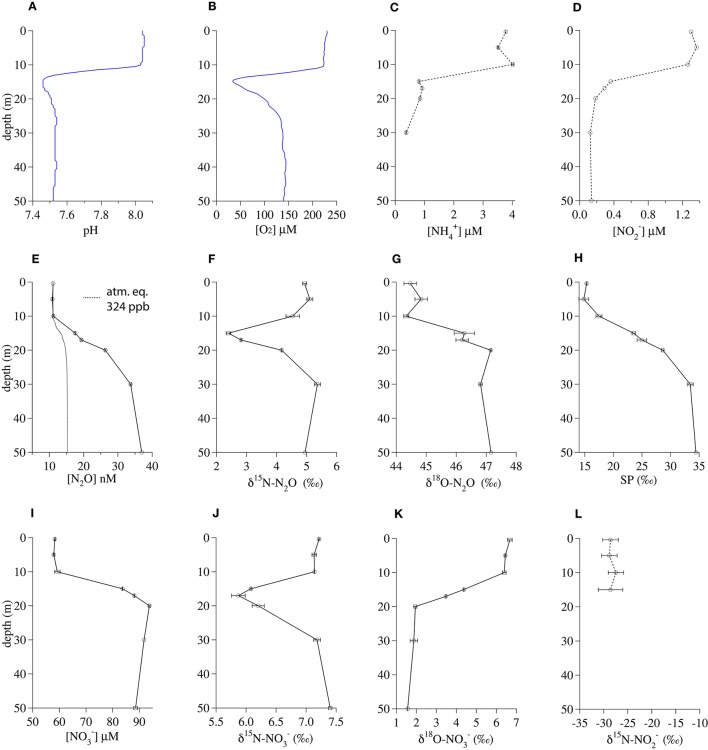
**Geochemical profiles from November 2013 in the south basin of Lake Lugano. (A)** pH, **(B)** O_2_ concentration, **(C)** ${\text{NH}}_{4}^{+}$concentration, **(D)** ${\text{NO}}_{2}^{-}$ concentration, **(E)** N_2_O concentration, **(F)** δ^15^ N-N_2_O, **(G)** δ^18^ O-N_2_O, **(H)** N_2_O Site Preference, **(I)** ${\text{NO}}_{3}^{-}$ concentration, **(J)** δ^15^ N-${\text{NO}}_{3}^{-}$, **(K)** δ^18^ O-${\text{NO}}_{3}^{-}$, **(L)** δ^15^ N-${\text{NO}}_{2}^{-}$. Bars indicate standard deviations among duplicate measurements.

The 17-m incubation depth in Lake Lugano corresponded to the shallow local minima in both pH (7.47) and O_2_ concentration (58 μM; Figures 2A,B). Below 15 m, the respective ${\text{NH}}_{4}^{+}$ and ${\text{NO}}_{2}^{-}$ concentrations dropped from their surface values of ~3.8 μM and ~1.2 μM to ~0.9 μM and ≤ 0.3 μM, respectively (Figures 2C,D). In contrast, N_2_O concentrations increased steadily from a surface concentration of 11 nM to 37 nM at 50 m (ΔN_2_O = 22 nM; Figure 2E). The δ^15^N-N_2_O dropped from a surface value of 4.9‰ to a minimum of 2.4‰ at 15 m and then increased again to 4.9‰ at 50 m (Figure 2F). In contrast, the δ^18^O-N_2_O and SP increased in parallel, from the surface values of 44.5‰ and 15.3‰, respectively, to values of 47.2‰ and 34.4‰, respectively, at 50 m (Figures 2G,H). Like N_2_O, ${\text{NO}}_{3}^{-}$ concentrations increased between the surface (58 μM) and 50 m (92 μM; Figure 2I) and the δ^15^N-${\text{NO}}_{3}^{-}$ profile had a distinct minimum around 15–17 m (Figure 2J), corresponding with the δ^15^N-N_2_O minimum. However, the shape of the δ^18^O-${\text{NO}}_{3}^{-}$ profile was the mirror opposite of the δ^18^O-N_2_O profile, with δ^18^O-${\text{NO}}_{3}^{-}$ decreasing from 6.7‰ in the surface to 1.6‰ at 50 m (Figure 2K). Surface ${\text{NO}}_{2}^{-}$ was depleted in ^15^N relative to both ${\text{NO}}_{3}^{-}$ and N_2_O, with δ^15^N-${\text{NO}}_{2}^{-}$ values between −29‰ and −27‰ (Figure 2L).

With the onset of seasonal anoxia in the south basin of Lake Lugano (Lehmann et al., 2004; Blees et al., 2014), the sediments (95 m) and deep redox transition zone (70–90 m) become important in the production and consumption of deep N_2_O in this system (Freymond et al., 2013; Wenk et al., 2016). Here we have restricted our discussion to the top 50 m of the water column. See Wenk et al. (2014) and Wenk et al. (2016) for details about N cycle dynamics in the redox transition zone.

#### Namibian upwelling zone

**Figure 3:**
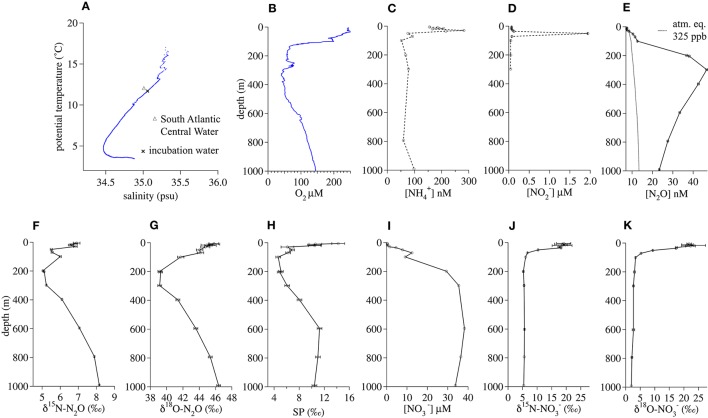
**Geochemical profiles from January 2014 in the Namibian Upwelling Zone (20.65°S, 10.95°E). (A)** Salinty vs. potential temperature, **(B)** O_2_ concentration, **(C)** ${\text{NH}}_{4}^{+}$ concentration, **(D)** ${\text{NO}}_{2}^{-}$ concentration, **(E)** N_2_O concentration, **(F)** δ^15^ N-N_2_O, **(G)** δ^18^ O-N_2_O, **(H)** N_2_O Site Preference, **(I)** ${\text{NO}}_{3}^{-}$ concentration, **(J)** δ^15^ N-${\text{NO}}_{3}^{-}$, **(K)** δ^18^ O-${\text{NO}}_{3}^{-}$.

The salinity (35.06) and potential temperature (11.74°C) of the water at the 200-m incubation depth (Figure 3A) was characteristic of a deeper tropical branch of South Atlantic Central Water that flows northwestward along the African continental shelf at this latitude (Brea et al., 2004). The *in situ* O_2_ concentration (56.8 μM) at 200 m fell within a broader O_2_-depleted zone extending from 150 to 500 m (Figure 3B). The incubation depth was well-below the depth of the ${\text{NH}}_{4}^{+}$ concentration maximum (30 m, Figure 3C) and the ${\text{NO}}_{2}^{-}$ maximum (50 m, Figure 3D). N_2_O oversaturation (ΔN_2_O) was greatest between 200 and 400 m (Figure 3E). At 200 m, there were minima in δ^15^N-N_2_O (5.1 ± 0.02‰, Figure 2F), δ^18^O-N_2_O (39.3 ± 0.24‰, Figure 3G), and SP (4.9 ± 0.4‰ Figure 3H). ${\text{NO}}_{3}^{-}$ concentrations were near zero in the surface water and increased with depth to a near-maximal concentration of 29 μM at 200 m (Figure 3I). At this depth, ${\text{NO}}_{3}^{-}$ had a δ^15^N (5.3 ± 0.2‰, Figure 3J) similar to that of N_2_O and a δ^18^O (3.0 ± 0.3‰, Figure 3K) that was much lower than that of N_2_O.

### Nitrification rates

#### Lake lugano

**Figure 4:**
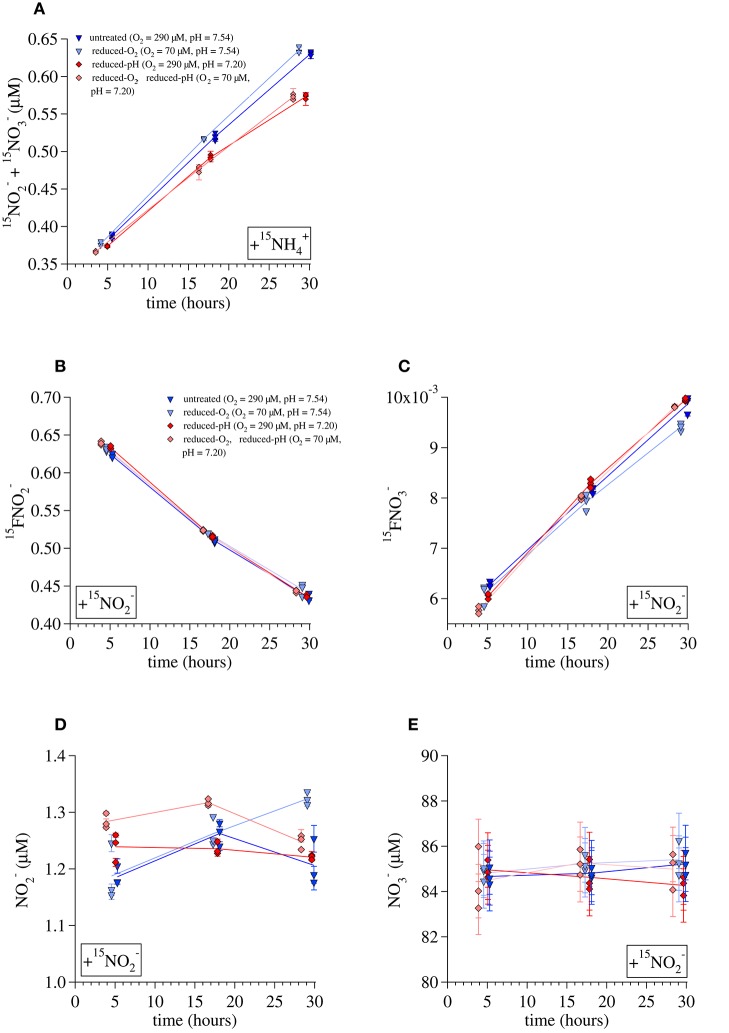
**Data used to calculate ammonia-oxidation rates during the Lake Lugano incubations. (A)** ^15^${\text{NO}}_{x}^{-}$ production during ^15^${\text{NH}}_{4}^{+}$ incubations, **(B)** ^15^${\text{F}}_{{\text{NO}}_{2}^{-}}$ during ^15^${\text{NO}}_{2}^{-}$ incubations, **(C)** ^15^${\text{F}}_{{\text{NO}}_{2}^{-}}$ during ^15^${\text{NO}}_{2}^{-}$ incubations, **(D)** ${\text{NO}}_{2}^{-}$ concentrations during ^15^${\text{NO}}_{2}^{-}$ incubations, and **(E)** ${\text{NO}}_{3}^{-}$ concentrations during ^15^${\text{NO}}_{2}^{-}$ incubations. Lines are drawn between averages of triplicate incubations at each time point. Error bars represent one standard deviation from the average of duplicate measurements.

Ammonia oxidation rates were estimated in two ways, using data from either the ^15^${\text{NH}}_{4}^{+}$ incubations or the ^15^${\text{NO}}_{2}^{-}$ incubations. The first method was by linear regression of the [^15^N_NOx−_] produced over time during the ^15^${\text{NH}}_{4}^{+}$ incubations (Figure 4A) multiplied by an isotopic dilution factor (1/^15^F_NH4+0_ = [^15^${\text{NH}}_{4_{0}^{+}}$ + ^14^${\text{NH}}_{4_{0}^{+}}$]/[^15^${\text{NH}}_{4_{0}^{+}}$]) calculated from the added tracer, the concentration of ${\text{NH}}_{4}^{+}$ present in the water before tracer addition, and assuming that this ${\text{NH}}_{4}^{+}$ had a δ^15^N of 0‰. The pH reduction from 7.54 to 7.20 was associated with a 12% decrease in ammonia oxidation rates in incubations at the untreated-O_2_ concentrations (rates calculated in this way were 0.356 ± 0.006 μM/day in the untreated-pH incubations and 0.314 ± 0.027 μM/day in the reduced-pH incubations). Among the reduced-O_2_ incubations, those with a reduced pH had 14% lower ammonia oxidation rates than those at the untreated pH (0.325 ± 0.004 μM/day and 0.380 ± 0.005 μM/day, respectively). Because we did not measure the ^15^F_NH4+_ over the course of the ^15^${\text{NH}}_{4}^{+}$ incubations, we were not able to account for the dilution of the tracer ^15^${\text{NH}}_{4}^{+}$ over time by regeneration of ${\text{NH}}_{4}^{+}$, or removal of ${\text{NH}}_{4}^{+}$ from the system by processes other than ammonia oxidation (Ward and Kilpatrick, 1990). Therefore, in a second approach, we used the results of the incubations with ^15^${\text{NO}}_{2}^{-}$ and NA ${\text{NH}}_{4}^{+}$ to calculate zero-order rates of ammonia oxidation (R_amm_ox_) and nitrite oxidation (R_nit_ox_). Specifically, rates were calculated for each timepoint (t) using measurements of the ^15^N atom fractions of ${\text{NO}}_{2}^{-}$ (^15^${\text{F}}_{{\text{NO}}_{2}^{-}}$, Figure 4B) and ${\text{NO}}_{3}^{-}$ (^15^${\text{F}}_{{\text{NO}}_{3}^{-}}$, Figure 4C), and the total concentrations of ${\text{NO}}_{2}^{-}$ ([${\text{NO}}_{2}^{-}$], Figure 4D) and ${\text{NO}}_{3}^{-}$ ([${\text{NO}}_{3}^{-}$], Figure 4E) to solve the following equations:

(2) $$\begin{array}{l} {[}^{14}{\text{NO}}_{2}^{-}](\text{t})={\text{R}}_{\text{amm\_ox}}\times \text{t}-(1{-}^{15}{\text{F}}_{{\text{NO}}_{2}^{-}})\times {\text{R}}_{\text{nit\_ox}}\times \text{t}\\ \;+{{[}^{14}{\text{NO}}_{2}^{-}]}_{0} \end{array}$$

(3) $${[}^{15}{\text{NO}}_{2}^{-}](\text{t})\;=-{(}^{15}{\text{F}}_{{\text{NO}}_{2}^{-}})\times {\text{R}}_{\text{nit\_ox}}\times \text{t}+{{[}^{15}{\text{NO}}_{2}^{-}]}_{0}$$

(4) $${[}^{14}{\text{NO}}_{3}^{-}](\text{t})\;=(1{-}^{15}{\text{F}}_{{\text{NO}}_{2}^{-}})\times {\text{R}}_{\text{nit\_ox}}\times \text{t}+{{[}^{14}{\text{NO}}_{3}^{-}]}_{0}$$

(5) $${[}^{15}{\text{NO}}_{3}^{-}](\text{t})\;={(}^{15}{\text{F}}_{{\text{NO}}_{2}^{-}})\times {\text{R}}_{\text{nit\_ox}}\times \text{t}+{{[}^{15}{\text{NO}}_{3}^{-}]}_{0}$$

Ammonia oxidation rates calculated this way were similar but somewhat higher than those derived from the ^15^${\text{NH}}_{4}^{+}$ incubation data. Average rates calculated for incubations terminated at the middle timepoint (~17 h) and final timepoint (~30 h) were 0.50 ± 0.02 μmol/day for the untreated-O_2_—untreated-pH incubations, 0.48 ± 0.02 μM/day for the reduced-O_2_—untreated-pH incubations, 0.54 ± 0.01 μM/day for the untreated-O_2_—reduced-pH incubations, and 0.54 ± 0.02 μM/day for the reduced-O_2_—reduced-pH incubations. Rates are given with the standard deviation among the calculated rates for each of the 17 and 30-h time points. Values of R_nit_ox_ calculated for the ^15^${\text{NO}}_{2}^{-}$ incubations were higher than R_amm_ox_ under all conditions. The average R_nit_ox_ was 0.64 ± 0.06 μmol/day among the untreated-O_2_—untreated-pH incubations, 0.59 ± 0.05 μmol/day among the reduced-O_2_—untreated-pH incubations, 0.72 ± 0.03 μM/day among the untreated-O_2_—reduced-pH incubations, and 0.72 ± 0.05 μM/day among the reduced-O_2_—reduced-pH incubations. Thus, there was net consumption of ${\text{NO}}_{2}^{-}$ of 0.1–0.2 μM/day.

#### Namibian upwelling zone

In the Namibian Upwelling incubations, ammonia-oxidation rates calculated from linear regressions of ^15^${\text{NO}}_{x}^{-}$ measured at 12 and 24 h during ^15^${\text{NH}}_{4}^{+}$ incubations were two orders of magnitude lower than during the Lake Lugano incubations. Average rates were 3.0 ± 0.3 nM/day (*r*^2^ = 0.96) at 220 μM O_2_, 2.4 ± 0.3 nM/day (*r*^2^ = 0.91) at 50 μM O_2_, and 2.3 ± 0.2 nM/day (*r*^2^ = 0.98) at 20 μM O_2_ (Table 2).

**Table 2:** **Results of incubations in the Namibian Upwelling zone**.

**[O_2_] μM**	**N tracers**	**Incubation time (hours)**	**Ammonia oxidation rate (nM/day)**	**δ^15^N-N_2_O (‰)**	**stdev δ^15^N-N_2_O (‰)**	**δ^18^O-N_2_O (‰)**	**stdev δ^18^O-N_2_O (‰)**	**Total N_2_O (nmoles)**	**Error total N_2_O (nmoles)**	***n***
Kill controls	^15^${\text{NH}}_{4}^{+}$, NA NO_2_−	0		7.0	0.9	42.8	0.7			3
220 ± 12	^15^${\text{NH}}_{4}^{+}$, NA NO_2_−	24.0	3.0 ± 0.3	10.8	1.5	43.3	0.5	2.7	0.08	2
50 ± 10	^15^${\text{NH}}_{4}^{+}$, NA NO_2_−	26.0	2.4 ± 0.3	13.8	0.9	44.0	0.2	2.0	0.05	3
20 ± 10	^15^${\text{NH}}_{4}^{+}$, NA NO_2_−	24.1	2.3 ± 0.2	20.8	2.0	46.0	0.5	1.7	0.05	3
Kill controls	NA ${\text{NH}}_{4}^{+}$, ^15^NO_2_−	0		6.8	0.2	45.5	0.5			3
220 ± 12	NA ${\text{NH}}_{4}^{+}$, ^15^NO_2_−	23.9	–	7.3	0.1	45.6	0.1	2.5	0.08	2
50 ± 10	NA ${\text{NH}}_{4}^{+}$, ^15^NO_2_−	25.2	–	8.0	0.5	45.2	0.2	1.9	0.06	3
20 ± 10	NA ${\text{NH}}_{4}^{+}$, ^15^NO_2_−	24.0	–	11.3	1.8	44.5	0.5	1.5	0.04	3

### N_2_O production rates and yields

#### Lake lugano

**Figure 5:**
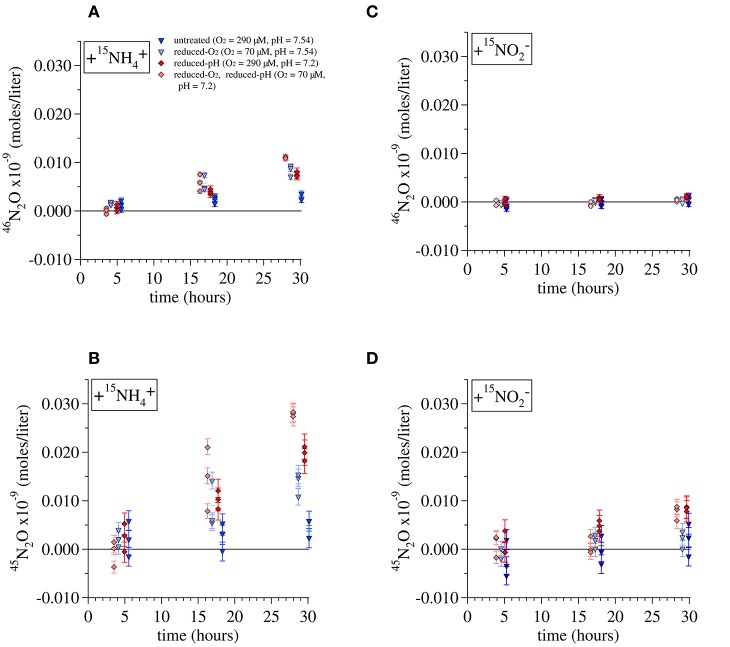
**Production of (A)** ^46^N_2_O and **(B)** ^45^N_2_O during incubations of Lake Lugano water with additions of 1 μM ^15^${\text{NH}}_{4}^{+}$ and 1 μM unlabeled ${\text{NO}}_{2}^{-}$. Production of **(C)** ^46^N_2_O and **(D)** ^45^N_2_O during incubations with 1 μM ^15^${\text{NO}}_{2}^{-}$ and 1 μM unlabeled ${\text{NH}}_{4}^{+}$. Error bars represent the propagated error from the measurements of the total N_2_O in each incubation, its constituent isotope ratios, and the volume of water present in each incubation. Lines are drawn to the average value of triplicate incubations at each time point.

Incorporation of tracer ^15^N into N_2_O over the course of the incubation can produce an excess of either m/z 45 N_2_O (total ^45^N_2_O = ^15^N^14^N^16^O + ^14^N^15^N^16^O + ^14^N^14^N^17^O) or m/z 46 N_2_O (total ^46^N_2_O = ^15^N^15^N^16^O + ^14^N^14^N^18^O). During the Lake Lugano incubations with ^15^${\text{NH}}_{4}^{+}$ + NA ${\text{NO}}_{2}^{-}$, significant amounts of both ^45^N_2_O and ^46^N_2_O were produced (Figures 5A,B). The quantities of ^45^N_2_O and ^46^N_2_O present in each incubation bottle were calculated by converting the calibrated molecular ratios (^45^R = [^45^N_2_O]/[^44^N_2_O] and ^46^R = [^46^N_2_O]/[^44^N_2_O]) to molecular fractions (^45^F = [^45^N_2_O]/[^44^N_2_O + ^45^N_2_O + ^46^N_2_O] and ^46^F = [^46^N_2_O]/[^44^N_2_O + ^45^N_2_O + ^46^N_2_O]):

(6) $${}^{45}\text{F}=1/(1{+}^{46}\text{R}{/}^{45}\text{R}+1{/}^{45}\text{R})$$

(7) $${}^{46}\text{F}=1/(1{+}^{45}\text{R}{/}^{46}\text{R}+1{/}^{46}\text{R})$$

and then multiplying the molecular fractions by the total N_2_O:

(8) $${}^{45}{\text{N}}_{2}\text{O}{=}^{45}\text{F }\times \;[\text{total }{\text{N}}_{2}\text{O}]$$

(9) $${}^{46}{\text{N}}_{2}\text{O}{=}^{46}\text{F }\times \;[\text{total }{\text{N}}_{2}\text{O}].$$

For each set of experimental conditions, the total ^45^N_2_O and ^46^N_2_O measured in incubations killed immediately after tracer addition (t_0_) was subtracted from the measurements of incubations killed at all subsequent time points. The ^45^R and ^46^R measured in incubations killed at t_0_ were similar to those of the background N_2_O, indicating that our preservation methods prevented further N_2_O production from either ^15^${\text{NH}}_{4}^{+}$ or ^15^${\text{NO}}_{2}^{-}$. Error bars in Figure 5 represent the propagated error from measurements of the total N_2_O in each incubation, its isotope ratios, and the total volume of water and background N_2_O present in each incubation bottle. Total daily incorporation of ^15^N into N_2_O during ^15^${\text{NH}}_{4}^{+}$ incubations was calculated from these daily rates as (Rate_45N2O_ + 2 × Rate_46N2O_). Rates of ^15^N incorporation were 0.0086 ± 0.003 nM-N/day for the untreated-O_2_—untreated-pH ^15^${\text{NH}}_{4}^{+}$ incubation, 0.025 ± 0.005 nM-N/day for the reduced-O_2_—untreated-pH incubations, 0.028 ± 0.003 nM-N/day for the untreated-O_2_—reduced-pH incubations, and 0.043 ± 0.001 nM-N/day for the reduced-O_2_—reduced-pH incubation, where we have indicated ± one standard deviation from the daily average calculated using the final three incubations. Assuming that ${\text{NH}}_{4}^{+}$ is the ultimate source of all the N_2_O produced during these incubations, multiplying the rates of ^15^N incorporation by their respective isotope dilution factors (1/^15^${\text{F}}_{{\text{NH}}_{4}^{+}0}$) would increase total N_2_O production rates by factors of 1.50 for the untreated-O_2_—untreated-pH and reduced-O_2_—untreated-pH incubations, and 1.61 for the untreated-O_2_—reduced-pH and reduced-O_2_—reduced-pH incubations.

Multiplying rates by 1/^15^${\text{F}}_{{\text{NH}}_{4}^{+}0}$ does not account for incorporation of N from exogenous ${\text{NO}}_{2}^{-}$ into N_2_O, which probably also contributed to N_2_O production, particularly in the reduced-pH incubations. Indeed, during incubations with ^15^${\text{NO}}_{2}^{-}$, significant amounts of ^45^N_2_O formed during both the reduced-pH and reduced O_2_—reduced-pH incubations (*t*-test, *p* = 0.012 and 0.022, respectively; Figure 5D), indicating that ^15^N derived from tracer ^15^${\text{NO}}_{2}^{-}$ also contributed to N_2_O production. However, no significant ^46^N_2_O production was observed among any of the ^15^${\text{NO}}_{2}^{-}$ incubations (Figure 5C), including those that produced significant amounts of ^45^N_2_O.

The yield of N_2_O during the ^15^${\text{NH}}_{4}^{+}$ incubations was calculated as the rate of incorporation of ^15^N into N_2_O relative to the rate of ^15^${\text{NO}}_{x}^{-}$ production. The average yields (mol ^15^N-N_2_O/mol ^15^N-${\text{NO}}_{x}^{-}$) were 3.64 × 10^−5^ for the untreated-O_2_—untreated-pH incubations, 10.0 × 10^−5^ for the reduced-O_2_—untreated-pH incubations, 14.0 × 10^−5^ for the untreated-O_2_—reduced-pH incubations and 21.0 × 10^−5^ for the reduced-O_2_—reduced-pH incubations.

#### Namibian upwelling zone

Among the Namibian Upwelling incubations, there was detectable production of ^45^N_2_O when 1 μM ^15^${\text{NH}}_{4}^{+}$ + 1 μM NA ${\text{NO}}_{2}^{-}$ was added, but not when 1 μM NA ${\text{NH}}_{4}^{+}$ + 1 μM ^15^${\text{NO}}_{2}^{-}$ was added. The increases in ^45^N_2_O were too small to be converted to significant daily rates of ^45^N_2_O production, and therefore results are reported in Table 2 using the more sensitive delta notation where the δ^15^N-N_2_O signal increases in proportion to [^45^N_2_O]/[^44^N_2_O], and the δ^18^O-N_2_O signal increases in proportion to [^46^N_2_O]/[^44^N_2_O]. Increases in δ^15^N-N_2_O over 24 h were higher during the ^15^${\text{NH}}_{4}^{+}$ incubations (10.8 ± 1.5‰ to 20.8 ± 2‰) than during the ^15^${\text{NO}}_{2}^{-}$ incubations (7.3 ± 0.1‰ to 11.3 ± 1.8‰). During the ^15^${\text{NH}}_{4}^{+}$ incubations, increases in δ^18^O-N_2_O (43.3 ± 0.5‰ to 46.0 ± 0.5‰) were smaller than the increases in δ^15^N-N_2_O. The increases in δ^18^O-N_2_O during the 20 and 50 μM-O_2_ incubations were significantly higher than the δ^18^O of the background N_2_O (two-tailed *p* = 0.003 and 0.0462, respectively for the 20 μM- and 50 μM-O_2_ incubations). The ^15^${\text{NH}}_{4}^{+}$ incubations with the lower O_2_ concentrations showed the largest increases in δ^15^N-N_2_O. This was partly due to the fact that headspace O_2_ displacement with He and N_2_ also reduced background N_2_O in the reduced-O_2_ incubations (Table 2). However, correction for the differences in background N_2_O among O_2_ treatments explains less than half of the increase in δ^15^N-N_2_O during the 20 μM-O_2_ incubations as compared to the 220 μM-O_2_ incubations.

### Nitrifier community composition

#### Overall microbial diversity and abundances

The OTU abundances and their taxonomic affiliation, closest known cultured relatives, and the number of reads assigned to each OTU are summarized in Tables S1, S2. Three separate reactions with DNA extracted from the Lake Lugano water (17 m depth) yielded 382,374, 77,700, and 287,306 partial 16S rRNA gene sequence reads. Three separate reactions with DNA from the Namibian Upwelling incubation water yielded 246,743, 23,288, and 154,829 reads. These reads underwent quality filtration and de-noising to produce a total of 271,425 unique OTUs that were ~253 bp long. Figure S5 summarizes the bacterial and archaeal phyla into which these OTUs fall. Comparison of the observed taxon richness to Chao1-estimated richness revealed that multiplex sequencing coverage was 47.9 ± 1.4% in the Lake Lugano sample and 52.3 ± 3.7% in the Namibian Upwelling sample. Rarefaction analyses that assess taxon richness in the Namibian Upwelling and Lake Lugano samples were generated with the QIIME pipeline (see Figure S4; Caporaso et al., 2012). The Shannon-Weiner diversity index (H), which is directly proportional to the number of taxa and inversely proportional to the number of sequences falling into each taxon, was an order of magnitude higher for AOB in Lake Lugano than for AOB in the Namibian Upwelling, whereas this index was higher for AOA in the Namibian Upwelling than in Lake Lugano (Figure S6).

#### AOA and AOB diversity and abundances

**Figure 6:**
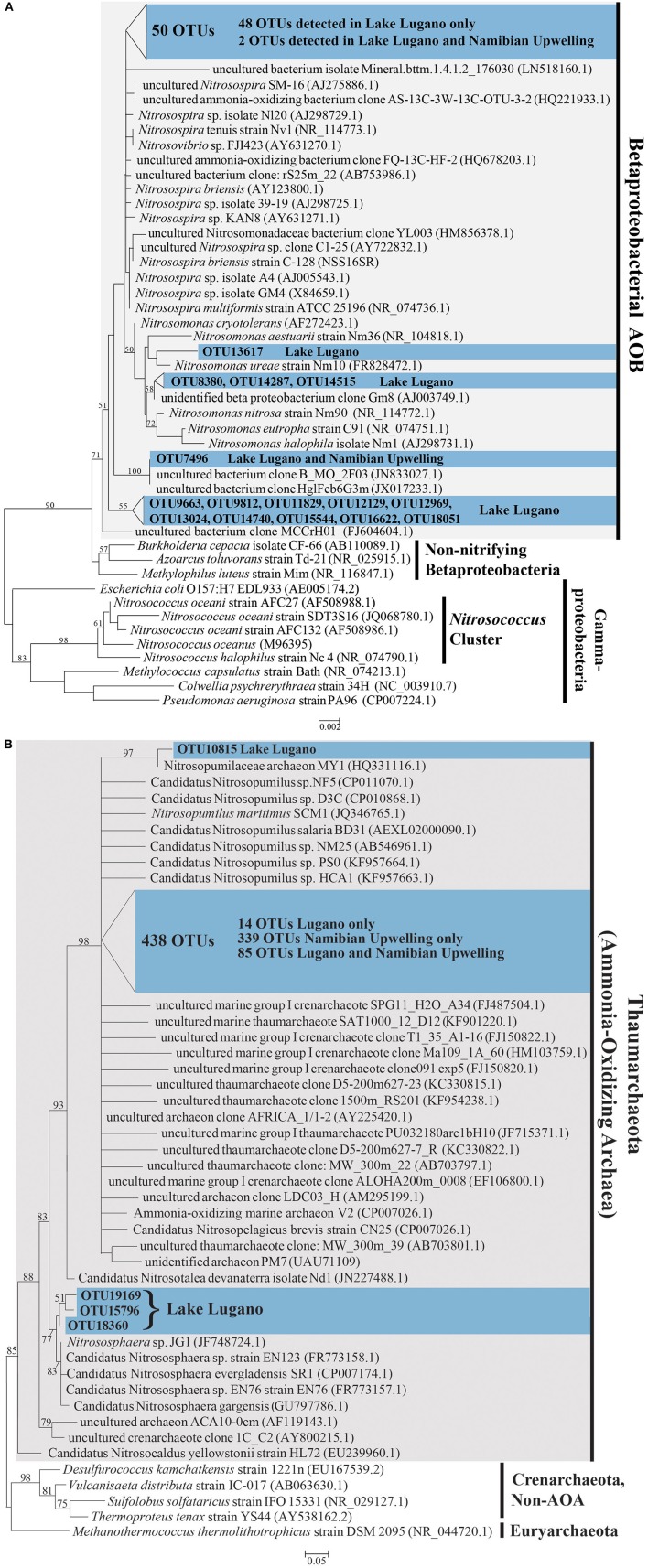
**(A)** Phylogenetic tree based on maximum likelihood (ML) analysis of 65 OTUs (~253 bp) detected in this study (in blue) in comparison with their close relatives and representatives from the Nitrosomonadaceae in the Betaproteobacteria. The locations where these OTUs were detected are indicated (Lake Lugano or Namibian Upwelling) and their accession numbers are LN908721-LN908785. **(B)** Phylogenetic tree based on maximum likelihood (ML) analysis of 442 OTUs (~253 bp) detected in this study (in blue) in comparison with their close relatives and representatives among the Thaumarchaeota. The locations where these OTUs were detected are indicated (Lake Lugano or Namibian Upwelling) and their accession numbers are LN908279-LN908720. Bootstrap values from 1000 replicates are indicated at the nodes of branches (if > 50). The scale bar represents the number of substitutions per site.

A total of 442 unique OTUs related to AOA and 65 unique OTUs related to the AOB family Nitrosomonadaceae were identified. The AOA OTUs constitute 0.3 and 31.2% of total microbial OTUs in Lake Lugano and the Namibian Upwelling site, respectively. AOB OTUs constituted 0.6% of total microbial OTUs in the Lake Lugano sample, but were extremely rare (< 0.01%) in the Namibian Upwelling sample. Both ML and MP trees were nearly identical in their placement of AOA and AOB OTUs with respect to their closest relatives. The 65 AOB OTUs fell into a monophyletic cluster that included cultured members of the family Nitrosomonadaceae (Figure 6A). The closest cultured representative to 27 of the 65 OTUs was the predominantly terrestrial species *Nitrosospira briensis* (with 91–100% 16S rRNA sequence identity), although their closest relatives were all uncultured freshwater organisms (see Figure 6A; Table S1). Most of the AOB OTUs (62) were detected only in the Lake Lugano sample and not the Namibian Upwelling sample, and the remaining 3 OTUs were present in both the Lake Lugano and Namibian Upwelling samples (Table S1).

The 442 unique AOA sequences fell into a clade with members of the Thaumarchaea (marine group I archaea) such as *Nitrosopumilus maritimus* SCM1 and *Nitrososphaera* sp. JG1, with moderate bootstrap support (Figure 6B). The closest cultured relatives of these OTUs were mainly found in seawater, with the majority most closely related to *Candidatus* Nitrosopelagicus brevis strain CN25 (with 88–100% sequence identity), and a large number most closely related to *Candidatus* Nitrosopumilus sp. NF5 and *Candidatus* Nitrosopumilus sp. D3C (with 86–98% sequence identity; see Figure 6B; Table S2). With a few exceptions, the uncultured closest relatives of these OTUs were also detected in seawater (Figure 6B). Of the 442 AOA OTUs identified, 339 were detected only in the Namibian Upwelling sample, 18 were detected only in Lake Lugano, and 85 were detected in both locations (Table S2). Among the AOA OTUs unique to Lake Lugano, their closest cultured relatives include *Candidatus* Nitrosopelagicus brevis strain CN25 (Santoro et al., 2015) and *Candidatus* Nitrosopumilus sp. HCA1 (KF957663.1) (Bayer et al., 2016), which are both marine, and also *Nitrososphaera viennensis* EN76 (Stieglmeier et al., 2014a) and *Candidatus* Nitrososphaera evergladensis SR1 (Zhalnina et al., 2014), which were both found in soil.

## Discussion

### Geochemical profiles

The profiles of the N_2_O concentration and isotopic composition were useful as qualitative indicators of the depths of rapid nitrification in Lake Lugano. Although the depth of the water used for the Lake Lugano experiments (17 m) was shallower than the N_2_O concentration maximum, its coincidence with a clear minimum in the δ^15^N-N_2_O profile (Figure 2F) suggests that there was rapid *in situ* N_2_O production there. However, the absence of a corresponding extremum in the SP profile (Figure 2H) at this depth suggests that the δ^15^N-N_2_O minimum probably reflects a minimum in the δ^15^N of the precursor N molecule, rather than a change in the mechanism of N_2_O formation at this depth. While there is also a minimum in the δ^15^N-${\text{NO}}_{3}^{-}$ profile at the incubation depth, the δ^18^O-${\text{NO}}_{3}^{-}$ profile indicates that ${\text{NO}}_{3}^{-}$ was probably not the precursor of N_2_O at this depth. More precisely, at this depth, the δ^18^O-N_2_O was 44‰ higher than the δ^18^O-${\text{NO}}_{3}^{-}$, whereas the combination of a 42‰ branching isotope effect (Casciotti et al., 2007; Frame et al., 2014) and a −22‰ kinetic isotope effect (Granger et al., 2006) associated with ${\text{NO}}_{3}^{-}$ reduction to N_2_O, should have produced N_2_O with a δ^18^O that was at most only 20‰ higher than the δ^18^O-${\text{NO}}_{3}^{-}$.

The similarity of the δ^15^N-N_2_O and δ^15^N-${\text{NO}}_{3}^{-}$ profiles suggests that both compounds are derived from a shared pool of relatively low-δ^15^N precursor N, which could be either ${\text{NH}}_{4}^{+}$ or ${\text{NO}}_{2}^{-}$. The δ^15^N-${\text{NO}}_{2}^{-}$ in the top 15 m ranged between −29‰ and −27‰ (Figure 2L). The NH_3_ oxidized to ${\text{NO}}_{2}^{-}$ at these depths could have been relatively depleted in ^15^N because of rapid remineralization of isotopically lighter organic N. The 20‰ equilibrium isotope effect between ${\text{NH}}_{4}^{+}$ and NH_3_ (Hermes et al., 1985) and/or expression of the isotope effect(s) associated with ammonia oxidation (Casciotti et al., 2003) may have also contributed to production of ^15^N-depleted N_2_O by ammonia oxidizers at this depth.

N_2_O production in the deeper water of this basin has been linked to a NH_2_OH-decomposition pathway, largely by the SP value of the N_2_O, which approaches a value of ~34‰ in the oxic water between 30 and 70 m (Wenk et al., 2016 and Figure 2H). This particular N_2_O formation mechanism happens during ammonia oxidation in aerobic conditions (Sutka et al., 2006; Frame and Casciotti, 2010; Santoro et al., 2011). Interestingly, such a high SP value is also observed in N_2_O that is formed abiotically by either the hybrid reaction of NH_2_OH with HNO_2_/${\text{NO}}_{2}^{-}$ or by the oxidation of NH_2_OH (Figure 1, pathways 1 and 2; Heil et al., 2014). Thus the production of high SP (~34‰) N_2_O often observed among AOB and AOA cultures may not distinguish a pathway involving only NH_2_OH from a hybrid pathway where N_2_O is formed via the reduction of HNO_2_/${\text{NO}}_{2}^{-}$ by NH_2_OH. To our knowledge, no data has been reported on the SP of N_2_O produced by the enzyme-catalyzed reaction of NH_2_OH and HNO_2_ described by Hooper (1968), but it seems reasonable to assume that it may also be ~34‰. Thus, while the SP of the N_2_O present in the shallower depths of Lake Lugano increases steadily between 5 and 50 m (Figure 2H), suggesting a NH_2_OH-dependent N_2_O formation mechanism, we cannot use SP alone to distinguish N_2_O produced by the reaction between NH_2_OH and ${\text{NO}}_{2}^{-}$ from N_2_O produced by NH_2_OH autoxidation or disproportionation.

The high concentration of N_2_O that had accumulated at the depth of the Namibian Upwelling incubation had a relatively low SP (Figure 3H), suggesting that the source of this N_2_O was either ${\text{NO}}_{x}^{-}$ reduction by denitrification or ${\text{NO}}_{2}^{-}$ reduction by nitrifier denitrification (Toyoda et al., 2005; Frame and Casciotti, 2010). However, in our incubations we observed hybrid N_2_O formation rather than denitrification or nitrifier denitrification. The absence of denitrification and nitrifier denitrification during the incubations is unsurprising, given the relatively high O_2_ concentrations (20, 50, or 220 μM), all of which were well above thresholds that limit transcription of *norB* in denitrifiers (Dalsgaard et al., 2014) and initiation of nitrifier denitrification by AOB (Frame and Casciotti, 2010). The lower O_2_ concentrations tested during these incubations were similar to the *in situ* O_2_ concentration at this depth (56.8 μM), suggesting that if denitrification or nitrifier denitrification had occurred in this water mass, it was not happening at the time and location where we sampled it. Frame et al. (2014) have argued that in this region of the South Atlantic, transport and mixing of continental shelf water that is O_2_-depleted and contains N_2_O produced by anaerobic or suboxic processes with relatively O_2_-rich offshore water, can produce water that contains relatively high O_2_ concentrations and also N_2_O with isotopic signatures that are characteristic of low-O_2_ processes like denitrification or nitrifier denitrification.

### Nitrification rates

The difference in ammonia oxidation rates (R_amm_ox_) calculated during incubations with ^15^${\text{NH}}_{4}^{+}$ vs. those with ^15^${\text{NO}}_{2}^{-}$ is probably the result of dilution and loss of the added ^15^${\text{NH}}_{4}^{+}$ due to rapid ${\text{NH}}_{4}^{+}$ regeneration and uptake, which would both tend to reduce our estimate of R_amm_ox_ during the ^15^${\text{NH}}_{4}^{+}$ incubations. It also suggests that the rate reduction that we observed in the reduced-pH ^15^${\text{NH}}_{4}^{+}$ incubations does not necessarily reflect an actual reduction in the rate of ammonia oxidation, but instead a more rapid reduction in the ^15^F_NH4+_ over time as compared to the control-pH incubations.

In both Lake Lugano and the Namibian Upwelling, zero-order reaction kinetics were assumed for ammonia oxidation, rather than first-order or Michaelis-Menten kinetics. This was probably a reasonable assumption in the Namibian Upwelling experiments, because AOA have an extremely high affinity for NH_3_/${\text{NH}}_{4}^{+}$, with half-saturation constants (K_m_) on the order of 100 nM (Martens-Habbena et al., 2009; Horak et al., 2013). In contrast, AOB have a lower affinity for ${\text{NH}}_{4}^{+}$ than marine assemblages of AOA (Horak et al., 2013; Newell et al., 2013). The lowest reported K_m_ among cultivated AOB representatives is 6 μM, and the typical range for cultivated AOB is 0.05–14 mM (Knowles et al., 1965; Keener and Arp, 1993; Martens-Habbena et al., 2009; Jiang and Bakken, 1999b). ${\text{NH}}_{4}^{+}$ concentrations during the incubations remained well above the K_m_ of AOA but below the K_m_ reported for AOB. Since both AOB and AOA were present in the Lake Lugano incubations, we used the results of the ^15^${\text{NO}}_{2}^{-}$ incubations to model first-order rate constants for ammonia oxidation that were 0.46 ± 0.04 M^−1^ day^−1^ for the untreated-O_2_—untreated-pH incubations, 0.45 ± 0.04 M^−1^ day^−1^ for the reduced-O_2_—untreated pH incubations, 0.47 ± 0.04 M^−1^ day^−1^ for the untreated-O_2_—reduced-pH incubations, and 0.42 ± 0.04 M^−1^ day^−1^ for the reduced-O_2_—reduced-pH incubations. Using the observed concentration of ${\text{NH}}_{4}^{+}$ (0.92 μM) at 17 m in Lake Lugano and assuming that this concentration is in steady-state, the actual ammonia oxidation rate may be slightly lower than what we calculated using the zero-order reaction model.

### N_2_O yields and mechanisms of N_2_O formation in lake lugano

Total N_2_O yields measured in Lake Lugano (3.64 × 10^−5^ to 21.0 × 10^−5^ mol ^15^N-N_2_O / mol ^15^N-${\text{NO}}_{\text{x}}^{-}$) were comparable to those observed by Yoshida et al. (1989) in the western North Pacific, also using ^15^N tracer techniques (8 to 54 × 10^−5^). They were at the low end of the range observed during growth of batch cultures of the AOB *Nitrosomonas marina* (10 to 60 × 10^−5^) at similar O_2_ concentrations (Frame and Casciotti, 2010) and were lower than those observed for batch cultures of the AOA *N. maritimus* (60 to 100 × 10^−5^) and *N. viennensis* (140 to 180 × 10^−5^) in media buffered to pH 7.5 (Stieglmeier et al., 2014b). The N_2_O formed during the Lake Lugano incubations was largely derived from intermediates or products of the ammonia oxidation reactions, and relatively little N was incorporated from exogenous ${\text{NO}}_{2}^{-}$, as indicated by the higher rates of ^15^N-N_2_O formation during the ^15^${\text{NH}}_{4}^{+}$ incubations than during the ^15^${\text{NO}}_{2}^{-}$ incubations (Figures 5A–D).

During all of the ^15^${\text{NH}}_{4}^{+}$ incubations, the measured ratio of ^46^N_2_O:^45^N_2_O production (0.38–0.67) was lower than the expected ratio produced by random pairing of N derived from ${\text{NH}}_{4}^{+}$ with the isotope ratio ^15^F_NH4+0_ (expected ^46^N_2_O/^45^N_2_O = (^15^F_NH4+0_)^2^ / (2 × ^15^F_NH4+0_ × (1−^15^F_NH4+0_)) = 1.0 for the untreated-O_2_—untreated-pH and reduced-O_2_—untreated-pH incubations and 0.82 for the untreated-O_2_—reduced-pH and reduced-O_2_—reduced-pH incubations). Interestingly, Jung et al. (2014) also report relatively high ^45^N_2_O production compared to ^46^N_2_O production during tracer incubations of AOB and soil AOA cultures in the presence of 99% ^15^${\text{NH}}_{4}^{+}$ and excess N.A. ${\text{NO}}_{2}^{-}$. Because they were working with laboratory cultures, their experiments started with almost no background ^14^${\text{NH}}_{4}^{+}$ and contained no ammonium regenerating processes that would increase ^14^${\text{NH}}_{4}^{+}$ over the course of the incubations, allowing them to attribute the ^45^N_2_O production to a reaction between ^15^${\text{NH}}_{4}^{+}$-derived N and ${\text{NO}}_{2}^{-}$-derived N.

In the present study, incubations with ^15^${\text{NH}}_{4}^{+}$ at the reduced pH produced lower ratios of ^46^N_2_O:^45^N_2_O than incubations at the untreated pH. Three factors may account for the difference: (1) the ^15^F_NH4+_0_ was lower among the reduced-pH incubations (0.62) than it was among incubations at the untreated pH (0.67), (2) there may have been differences in the regeneration rates of ${\text{NH}}_{4}^{+}$ among experimental treatments, which would have progressively reduced the ^15^F_NH4+_ at different rates in the two different pH treatments, and (3) an increased contribution of N from an unlabeled N pool (i.e., exogenous ${\text{NO}}_{2}^{-}$) enhanced ^45^N_2_O production. Although we lack knowledge of the evolution of ^15^F_NH4+_ during our experiments that would allow us to rule out factors (1) and (2), we can be certain that the more rapid production of ^45^N_2_O during the reduced-pH ^15^${\text{NO}}_{2}^{-}$ incubations was at least partly the result of additional N-incorporation from exogenous ${\text{NO}}_{2}^{-}$, given the production of ^45^N_2_O in the ^15^${\text{NO}}_{2}^{-}$-amended experiment (Figure 5D), and that this argues in favor of factor (3) discussed above.

Nitrifier denitrification is unlikely to have contributed to N_2_O production during the Lake Lugano experiments. During the ^15^${\text{NO}}_{2}^{-}$ incubations with untreated-O_2_—reduced-pH and reduced-O_2_—reduced-pH, the formation of ^45^N_2_O and not ^46^N_2_O (Figures 5C,D) suggests that nitrifier denitrification was not important. Although cultured representatives of *Nitrosospira*, the most abundant AOB genus in the Lake Lugano, are known to contain *norB* homologs (Garbeva et al., 2007), whose enzyme products reduce NO to N_2_O during nitrifier denitrification reactions (Figure 1, yellow box; Schmidt et al., 2004; Kozlowski et al., 2014), the incubation conditions, such as the relatively high O_2_ concentrations (70 and 290 μM), were unlikely to have stimulated nitrifier denitrification. Rather, a hybrid N_2_O formation mechanism (Figure 1, pathway 2) that combines one N derived from exogenous ${\text{NO}}_{2}^{-}$ with one N derived from a different, unlabeled N pool, explains the formation of ^45^N_2_O in the absence of ^46^N_2_O formation during the ^15^${\text{NO}}_{2}^{-}$ incubations. Stieglmeier et al. (2014b) also observed hybrid N_2_O formation by AOA cultures, and have suggested that this other pool of N is NH_2_OH. NH_2_OH is known to form N_2_O in the presence of ${\text{NO}}_{2}^{-}$, both enzymatically (Hooper, 1968) and abiotically (Döring and Gehlen, 1961). Since both ${\text{NO}}_{2}^{-}$ and NH_2_OH form in the periplasm (Hollocher et al., 1982), a reaction between these two compounds during the ^15^${\text{NH}}_{4}^{+}$ incubations is also consistent with the relatively high rates of both ^45^N_2_O and ^46^N_2_O production (Figures 5A,B). However, without knowledge of ^15^F_NH4+_ over the course of the incubations, we cannot rule out formation of some ^15^N-N_2_O through NH_2_OH autoxidation or disproportionation (Figure 1, pathway 1). A third possibility is that two intracellular ${\text{NO}}_{2}^{-}$ molecules react with each other to form N_2_O via nitrifier denitrification in a system where mixing between endogenous (i.e., periplasmic) ${\text{NO}}_{2}^{-}$ and exogenous ${\text{NO}}_{2}^{-}$ occurs so slowly that the two ${\text{NO}}_{2}^{-}$ pools have distinct isotopic compositions. In the ^15^${\text{NH}}_{4}^{+}$ incubations, nitrifier denitrification of the relatively ^15^N-enriched periplasmic ${\text{NO}}_{2}^{-}$ would produce more ^45^N_2_O and ^46^N_2_O than we would predict based on the measured isotopic composition of the total ${\text{NO}}_{2}^{-}$. In the ^15^${\text{NO}}_{2}^{-}$ incubations, nitrifier denitrification of periplasmic ${\text{NO}}_{2}^{-}$ would mainly produce ^44^N_2_O, and only a small influx of exogenous ^15^${\text{NO}}_{2}^{-}$ would be reduced to ^45^N_2_O after mixing with unlabeled periplasmic ${\text{NO}}_{2}^{-}$ (as we observed during the reduced-pH incubations; Figure 5D). In this way, it would be possible for nitrifier denitrification to produce ^45^N_2_O and not ^46^N_2_O during the ^15^${\text{NO}}_{2}^{-}$ incubations. We believe that this pathway is unlikely given the relatively high O_2_ concentrations in our incubations, but without more detailed knowledge of the size of the periplasmic ${\text{NO}}_{2}^{-}$ pool maintained by the ammonia-oxidizing cells, and the rate at which this pool exchanges with the external ${\text{NO}}_{2}^{-}$ pool, we cannot completely exclude the possibility of nitrifier denitrification.

### Possible explanations for the influence of pH on N_2_O production

Reducing the pH during the Lake Lugano incubations increased ^15^N_2_O production in both the ^15^${\text{NH}}_{4}^{+}$ experiments and the ^15^${\text{NO}}_{2}^{-}$ experiments. There may be several reasons for this, including structural (e.g., the outer cell membrane may exchange HNO_2_/${\text{NO}}_{2}^{-}$ more rapidly between the periplasm and the outer environment at a lower pH), enzymatic (e.g., a shift toward the optimal pH of the N_2_O-producing enzymes in the periplasm), transcriptional (regulation of the genes encoding the enzymes involved in N_2_O production may be pH-sensitive), and chemical (due to acceleration in the rates of abiotic reactions that produce N_2_O from precursor molecules made by AOB). As discussed below, the most likely explanations are a shift toward the optimal pH of enzymes that catalyze N_2_O production and/or the involvement of a non-biological catalyst that accelerates the abiotic reactions that form N_2_O.

The majority of ammonia oxidizers in Lake Lugano are Gram-negative bacteria, which means that they have a periplasmic space that is bounded by inner and outer cell membranes. In other Gram-negative species such as *Escherichia coli*, the pH of the periplasm rapidly changes to reflect that of the external environment (Wilks and Slonczewski, 2007). To our knowledge, it is unknown whether AOB regulate the pH of their periplasm or not, but decreases in the periplasmic pH are likely to enhance the rates of the N_2_O-forming reactions of NH_2_OH and/or ${\text{NO}}_{2}^{-}$. Furthermore, if there are differences in the rate at which HNO_2_ vs. ${\text{NO}}_{2}^{-}$ cross the outer cell membrane, as suggested by Hollocher et al. (1982), then a pH shift could also alter the rate at which ^15^N from the tracer ^15^${\text{NO}}_{2}^{-}$ enters the periplasm and the rate at which the ${\text{NO}}_{2}^{-}$ formed during ammonia oxidation is expelled from the periplasm into the outer environment. This effect would not necessarily change the actual rate of N_2_O production, just our ability to observe it with ^15^N tracers. However, if this occurs, it is unlikely to be the dominant effect, because we observed increased ^15^N-N_2_O production during the reduced-pH incubations with ^15^${\text{NH}}_{4}^{+}$, and in this case, more rapid exchange of ${\text{NO}}_{2}^{-}$/HNO_2_ across the outer cell membrane in the reduced-pH incubations would dilute the periplasmic concentration of ^15^${\text{NO}}_{2}^{-}$, and therefore decrease the rate of ^15^N-N_2_O production relative to total N_2_O production.

The results of the ^15^${\text{NH}}_{4}^{+}$-incubations are consistent with an acceleration of the periplasmic reactions that form N_2_O. Formation of ^46^N_2_O, which is composed only of ^15^${\text{NH}}_{4}^{+}$-derived N (and should therefore be relatively independent of any influx of external ${\text{NO}}_{2}^{-}$ into the periplasm), was also faster at the reduced pH (Figure 5A). At the enzyme level, Hooper (1968) observed acceleration of N_2_O formation with decreasing pH: AOB enzyme extracts converted NH_2_OH + HNO_2_ to N_2_O with an optimum pH of 5.75, via a reaction whose rate increased steadily as the pH dropped from 7.5 to 6. Assuming that a similar reaction also occurs in intact AOB cells, the reaction rate increase observed by Hooper (1968) was large enough to explain the pH effect observed during the Lake Lugano incubations. Decreases in pH also have effects at the level of transcription and protein expression. For example expression of enzymes involved in handling nitrogen oxides in AOB, increases as the pH of the growth medium decreases from 8.2 to 7.2 (Beaumont et al., 2004a). The mechanism for this appears to depend on a transcriptional regulator whose repression is reversed by the presence of ${\text{NO}}_{2}^{-}$ at lower pH values (Beaumont et al., 2004a).

Without some form of catalysis, the rate constants reported for the abiotic hybrid N_2_O formation reaction between NH_2_OH and ${\text{NO}}_{2}^{-}$ are too small for these reactions to have contributed significantly to N_2_O production during our incubations. In particular, the set of reactions thought to produce N_2_O that contains one NH_2_OH-derived N and one HNO_2_-derived N, has a second-order dependence on [HNO_2_] (Döring and Gehlen, 1961; Bonner et al., 1983; Schreiber et al., 2012):

(10) $$\begin{array}{l} 2{\text{HNO}}_{2}↔{\text{NO}}_{2}+\text{NO}+{\text{H}}_{2}\text{O}\\ \text{ k}=1.6{\text{M}}^{-1}{\text{day}}^{-1} \end{array}$$

(Park and Lee, 1988)

(11) $$\begin{array}{l} \text{NO}+{\text{NO}}_{2}↔{\text{N}}_{2}{\text{O}}_{3}\\ \text{ k}=9.5\times 10^{7}\\ \;{\text{M}}^{-1}{\text{day}}^{-1} \end{array}$$

(Grätzelet et al., 1970)

(12) $$\begin{array}{l} {\text{N}}_{2}{\text{O}}_{3}+{\text{NH}}_{2}\text{OH}\to {\text{N}}_{2}\text{O}+{\text{HNO}}_{2}+{\text{H}}_{2}\text{O}\\ \text{ k}=1.7\times 10^{13}{\text{M}}^{-1}{\text{day}}^{-1}\; \end{array}$$

(Döring and Gehlen, 1961; Casado et al., 1983)

This rate-limiting step (i.e., Equation 10, the formation of NO by disproportionation of HNO_2_) becomes important at pH values <4.5 (Hooper, 1968), but it is not fast enough to explain ^45^N_2_O production during the incubations, given the low rate constant for HNO_2_ disproportionation and the low [HNO_2_] during the incubations (1.8 × 10^−11^ M at the untreated pH and 4.0 × 10^−11^ M at the reduced pH). Thus a role for a catalyst, whether enzymatic or non-biological, is indicated.

It is important to note that the NO reacting in Equation (11) may be formed through mechanisms other than the rate-limiting abiotic HNO_2_ disproportionation step. As mentioned earlier, a number of processes in ammonia oxidizers release NO. For example, nitrite reductases can convert ${\text{NO}}_{2}^{-}$ to NO. In AOB, NO is an intermediate in the catalytic cycle of hydroxylamine oxido-reductase (HAO) (Cabail and Pacheco, 2003) and may be released from NH_2_OH in HAO enzyme preparations (Hooper and Nason, 1965; Ritchie and Nicholas, 1972; Hooper and Terry, 1979). In AOA, NO is needed to oxidize NH_2_OH to ${\text{NO}}_{2}^{-}$ (Kozlowski et al., 2016). Abiotic reactions between NH_2_OH and NO observed by Bonner et al. (1978) (pH 7.8, anaerobic conditions), produced N_2_O that was ~75% composed of equal proportions of NH_2_OH-derived N and NO-derived N, and ~25% composed of only NO-derived N. If this reaction occurs during ammonia oxidation, then depending on whether NO is derived from NH_2_OH, ${\text{NO}}_{2}^{-}$, or both, these reactions could produce N_2_O that is entirely derived from NH_2_OH, or some mixture of hybrid N_2_O and N_2_O that is entirely derived from ${\text{NO}}_{2}^{-}$.

Chemodenitrification, the reduction of ${\text{NO}}_{3}^{-}$ or ${\text{NO}}_{2}^{-}$ coupled to oxidation of ferrous iron (Fe^2+^) (Buresh and Moraghan, 1976; Rakshit et al., 2008; Picardal, 2012) was an unlikely source of N_2_O during the Lake Lugano incubations. In the top 20 m of the lake, concentrations of metals involved in chemodenitrification (Fe and possibly manganese, Mn) were less than the 1.7 μM detection limit when measured by induction coupled plasma optical emission spectrometry (J. Tischer, U. Basel, unpublished data). Furthermore, the ^15^F_NO2−_ values during incubations with ^15^${\text{NO}}_{2}^{-}$ (Figure 4B) were high enough that if chemodenitrification of ${\text{NO}}_{2}^{-}$ to N_2_O had been significant, the observed production of ^45^N_2_O (Figure 5C) would have been accompanied by detectable ^46^N_2_O production (Figure 5D), and it was not. The ^15^F_NO3−_ values were lower than ^15^F_NO2−_ values (Figure 4C), so that mixed reduction of both ${\text{NO}}_{2}^{-}$ and ${\text{NO}}_{3}^{-}$ by Fe^2+^ could explain detectable ^45^N_2_O production in the absence of detectable ^46^N_2_O production. However, ${\text{NO}}_{3}^{-}$ reduction by Fe^2+^ is much slower than oxidation by ${\text{NO}}_{2}^{-}$, except when Cu^2+^ > 1.6 μM (Buresh and Moraghan, 1976; Picardal, 2012). We did not measure Cu concentrations in Lake Lugano, but the total dissolved Cu measured in Lake Greifen, a similarly eutrophic lake in northeastern Switzerland, were much lower than this (0.5–2.8 × 10^−8^ M; Xue and Sigg, 1993).

Although trace metal concentrations were low in Lake Lugano, metal ions could have played a role in accelerating the hybrid N_2_O reaction. Harper et al. (2015) have reported that Cu^2+^ can drive abiotic hybrid N_2_O formation rates in activated sludge that are faster than the biologically-catalyzed reactions. Furthermore, NH_2_OH disproportionation and oxidation reactions may be driven by the presence of copper and iron ions (Anderson, 1964; Alluisetti et al., 2004).

### Evidence for hybrid N_2_O formation in the Namibian Upwelling zone

The relationship between the changes in δ^15^N-N_2_O and δ^18^O-N_2_O during the Namibian Upwelling incubations were too small to convert to N_2_O production rates, but they still contain information about the mechanism of N_2_O formation. In particular, in light of the high degree of ^15^N labeling of the ${\text{NH}}_{4}^{+}$ pool (^15^F_NH4+_0_ = 0.94) that was achieved during the ^15^${\text{NH}}_{4}^{+}$ incubations, random pairing of ${\text{NH}}_{4}^{+}$-derived N atoms cannot explain the large increase in δ^15^N-N_2_O relative to δ^18^O-N_2_O. In the Supplementary Material S.2 we demonstrate that the change in δ^15^N-N_2_O relative to the change in δ^18^O-N_2_O observed during these incubations is not consistent with the formation of N_2_O composed only of N derived from ${\text{NH}}_{4}^{+}$. Specifically, N_2_O produced with a binomial distribution of ^15^N and ^14^N derived from ${\text{NH}}_{4}^{+}$ with this ^15^F_NH4+_0_ would produce a much larger increase in δ^18^O-N_2_O (i.e., more ^46^N_2_O) relative to the increase in δ^15^N-N_2_O (^45^N_2_O) than what was observed. In contrast, hybrid N_2_O formation (for example, by reaction of highly ^15^N-labeled NH_2_OH with unlabeled NO or ${\text{NO}}_{2}^{-}$) would produce a much larger increase in δ^15^N-N_2_O with almost no change in δ^18^O-N_2_O (Figure S3).

If hybrid N_2_O formation produced ^45^N_2_O during the ^15^${\text{NH}}_{4}^{+}$ incubations, from which N pool was the ^14^N atom derived? ^15^N was more rapidly incorporated into N_2_O during the ^15^${\text{NH}}_{4}^{+}$ incubations than during the ^15^${\text{NO}}_{2}^{-}$ incubations, as indicated by the larger increase in δ^15^N-N_2_O (^45^N_2_O) during the ^15^${\text{NH}}_{4}^{+}$ incubations (Table 2). If both ${\text{NH}}_{4}^{+}$ and exogenous ${\text{NO}}_{2}^{-}$ had contributed equally to N_2_O formation, then we would expect approximately equal increases in δ^15^N-N_2_O when either ^15^${\text{NH}}_{4}^{+}$ or ^15^${\text{NO}}_{2}^{-}$ was added (^15^F_*NO*2−_0_ = 0.95). The fact that the δ^15^N-N_2_O increased by less during the ^15^${\text{NO}}_{2}^{-}$ incubations than during the ^15^${\text{NH}}_{4}^{+}$ incubations suggests that exogenous ${\text{NO}}_{2}^{-}$ could not have been the sole source of ^14^N to ^45^N_2_O produced during the ^15^${\text{NH}}_{4}^{+}$ incubations. Possibly, an intracellular pool of unlabeled ${\text{NO}}_{2}^{-}$ was held over from before the ^15^${\text{NH}}_{4}^{+}$ incubation started. If there was enough of this holdover ${\text{NO}}_{2}^{-}$ to dilute any endogenous ^15^${\text{NO}}_{2}^{-}$ produced by ^15^${\text{NH}}_{4}^{+}$ oxidation, then reactions between ^15^NH_2_OH with the holdover ^14^${\text{NO}}_{2}^{-}$ would produce an increase in δ^15^N-N_2_O (^45^N_2_O) without increasing δ^18^O-N_2_O (^46^N_2_O).

Previous studies in aquatic systems have reported some variation in the importance of ${\text{NH}}_{4}^{+}$-derived N vs. ${\text{NO}}_{2}^{-}$-derived N to N_2_O production. Similar results to ours are reported for incubations of suboxic Black Sea water, where Westley et al. (2006) also found production of ^15^N-N_2_O with the addition of ^15^${\text{NH}}_{4}^{+}$ but not ^15^${\text{NO}}_{2}^{-}$. In the Eastern Tropical South Pacific, above the OMZ (O_2_ ≥ 10 μM), the rates of ${\text{NH}}_{4}^{+}$-derived N incorporation into N_2_O (0.01–0.02 nM/day) were similar to what was observed in Lake Lugano, although the yield (as defined in this paper) was substantially higher (80 × 10^−5^; Ji et al., 2015). In the North Pacific Gyre, Wilson et al. (2014) observed no changes in δ^15^N-N_2_O during incubations with either 1 μM NA ${\text{NH}}_{4}^{+}$ + 1 μM ^15^${\text{NO}}_{2}^{-}$ or 1 μM ^15^${\text{NH}}_{4}^{+}$ + 1 μM NA ${\text{NO}}_{2}^{-}$. Critically, however, when they reduced the NA ${\text{NH}}_{4}^{+}$ addition to 100 nM and added 1 μM ^15^${\text{NO}}_{2}^{-}$, the δ^15^N-N_2_O increased significantly over the course of the incubation. This suggests that the rate of ammonia oxidation influences the degree to which N derived from exogenous ${\text{NO}}_{2}^{-}$ can be incorporated into N_2_O, with higher rates of ammonia oxidation perhaps flooding the intracellular ${\text{NO}}_{2}^{-}$ pool and preventing N derived from exogenous ${\text{NO}}_{2}^{-}$ from being incorporated into N_2_O.

It is difficult at this point to determine whether the same hybrid N_2_O reaction mechanism(s) can explain the results of both the Lake Lugano incubations, which were numerically dominated by AOB, and the Namibian Upwelling incubations, which were dominated by AOA. To date, it is not known whether AOA enzymes also catalyze the reaction between NH_2_OH and ${\text{NO}}_{2}^{-}$ (Figure 1, pathway 2) as observed for AOB by Hooper (1968). The original AOB periplasmic enzyme complex purified in that study included HAO as well as other enzyme components. No homologs of *hao* have been identified in AOA genomes, though alternatives have been proposed (Stahl and de la Torre, 2012). Furthermore, if a periplasmic reservoir of ${\text{NO}}_{2}^{-}$ plays a role in the incorporation of ${\text{NO}}_{2}^{-}$-derived N into N_2_O, as we hypothesize here, then differences in the ${\text{NO}}_{2}^{-}$ permeability of the outer membranes/cell walls of AOB vs. AOA could contribute to differences in the degree to which N derived from ${\text{NH}}_{4}^{+}$ vs. exogenous ${\text{NO}}_{2}^{-}$ contribute to N_2_O formation. Unlike AOB, almost all archaea tested have only a single cell membrane bounding the cytoplasm (Albers and Meyer, 2011). Rather than having an outer membrane, AOA have an S-layer protein cell wall separating a pseudo-periplasm from the surrounding environment (Stieglmeier et al., 2014a). Model predictions of AMO protein structure in the soil AOA *Candidatus* Nitrosotalea devanterra suggest that the membrane-bound enzyme faces outward into the pseudoperiplasm (Lehtovirta-Morley et al., 2016). For future reference during ^15^N tracer studies of N_2_O production, it would be helpful to confirm that NH_2_OH and ${\text{NO}}_{2}^{-}$ both form in the pseudoperiplasm of AOA, and investigate what controls the rates at which exogenous ${\text{NO}}_{2}^{-}$ enters, and periplasmic ${\text{NO}}_{2}^{-}$ exits, this compartment.

### A putative link to O_2_

During both the Lake Lugano and Namibian Upwelling incubations, more ^15^N-N_2_O was produced at the reduced-O_2_ concentrations (O_2_ = 70 and 20 μM, respectively) than at the untreated-O_2_ concentrations (O_2_ = 290 and 220 μM, respectively). It is well known that N_2_O yields by AOB increase during growth at suboxic O_2_ concentrations (Goreau et al., 1980), and previous work on the mechanisms causing this increase implicated induction of the nitrifier denitrification pathway at very low O_2_ concentrations (e.g., Frame and Casciotti, 2010). Reducing O_2_ from 290 to 70 μM in the Lake Lugano incubations nearly tripled the yield of ^45^N_2_O and ^46^N_2_O during the ^15^${\text{NH}}_{4}^{+}$ incubations. However, the reason for this increase was probably not increased nitrifier denitrification, since there was no ^46^N_2_O production during any of the incubations with ^15^${\text{NO}}_{2}^{-}$ (Figure 5C).

The mechanism conferring this O_2_ sensitivity may not necessarily involve direct regulation of enzyme activity. In particular, NO removal by O_2_ is a possible abiotic NO-sink that would become more important at higher O_2_ and NO concentrations:

(13) $$\begin{array}{l} 2\text{NO}(\text{aq})+{\text{O}}_{2}(\text{aq})\;\to \;2{\text{NO}}_{2}(\text{aq})\\ \text{ k}=1.8\times 10^{11}{\text{M}}^{-2}{\text{day}}^{-1} \end{array}$$

(Awad and Stanbury, 1993)

Once NO_2_ is formed, in aqueous solutions it tends to react with water to form ${\text{NO}}_{3}^{-}$ and ${\text{NO}}_{2}^{-}$ (Park and Lee, 1988), or react with NO to form N_2_O_3_ (Grätzel et al., 1970). Martens-Habbena et al. (2015) measured NO concentrations of ~50–80 nM during oxic incubations of *N. maritimus* with 10 μM ${\text{NH}}_{4}^{+}$. If similar NO concentrations were produced during incubations in the present study, liquid-phase reactions between NO and O_2_ may deplete NO concentrations significantly, with the rate of depletion increasing in proportion to [O_2_] as well as [NO]^2^. Thus, abiotic reaction with O_2_ may compete for NO with N_2_O-forming reactions that also consume NO, particularly when incubation O_2_ concentrations are high.

Like O_2_, NO tends to partition into the gas phase over the aqueous phase (Schwartz and White, 1981). If it is NO (aq), rather than ${\text{NO}}_{2}^{-}$, that participates in biological hybrid N_2_O formation, an implication is that inclusion of a headspace during incubations of ammonia oxidizers suspended in water will reduce aqueous NO concentrations, and therefore slow down liquid-phase NO-dependent reactions (such as the reaction of NO with NH_2_OH to form N_2_O). Differences in aqueous NO concentrations might contribute to the discrepancy in the literature over whether reduced-O_2_ growth conditions increase the yields of N_2_O produced by AOA (e.g., Löscher et al., 2012; Stieglmeier et al., 2014b), particularly if there is variation in aeration procedures and ratios of headspace to liquid volumes.

## Conclusions

Previous studies have shown that decreases in pH can increase N_2_O production by AOB cultures (e.g., Jiang and Bakken, 1999a) but did not separate the effect of pH-dependent NH_3_ limitation from the influence of pH on the N_2_O production mechanisms. Here we have shown that acidification enhances the N_2_O yields of ammonia oxidizers even when it does not substantially change the ammonia oxidation rates. We have demonstrated that hybrid N_2_O formation (i.e., the combination of ${\text{NH}}_{4}^{+}$- and ${\text{NO}}_{2}^{-}$-derived N) occurs among the *Nitrosospira*-dominated ammonia oxidizer community in the shallow hypolimnion of Lake Lugano and that this mechanism contributes to the increased yield of N_2_O under acidified conditions. The ${\text{NH}}_{4}^{+}$-derived reactant in this hybrid N_2_O production pathway is probably NH_2_OH, while the ${\text{NO}}_{2}^{-}$-derived reactant could be one of several inter-convertible nitrogen oxides (${\text{NO}}_{2}^{-}$/HNO_2_, NO, N_2_O_3_). Our results suggest that nitrifier denitrification was not an important source of N_2_O in this environment. While N derived from exogenous ${\text{NO}}_{2}^{-}$ contributed significantly to N_2_O formation under acidified conditions, N derived from ${\text{NH}}_{4}^{+}$ was always a more important contributor to N_2_O. Finally, we report preliminary isotopic evidence that hybrid N_2_O formation also occurs among the subsurface AOA-dominated nitrifier community present in the Namibian Upwelling zone.

Our results are not necessarily predictive of the long-term influence of acidification on N_2_O production by ammonia oxidizers, since acidification may also change ammonia oxidizer community composition (Bowen et al., 2013) and pH decreases may have cascading chemical and biological effects in lake and ocean ecosystems. However, our results are applicable to environments that experience rapid changes in pH such as stratified lakes that undergo episodic mixing or rapid influx of acidified precipitation, and ocean upwelling zones where CO_2_-rich, low-pH deeper water may enhance N_2_O production when it comes in contact with shallower ammonia-oxidizing communities.

## Author contributions

CF conceived of and performed experiments. EL and EN analyzed and interpreted genetic sequence data. TG provided instrumentation support and sample analysis. CF and ML performed chemical data analysis and interpretation. All authors contributed to writing this paper.

### Conflict of interest statement

The authors declare that the research was conducted in the absence of any commercial or financial relationships that could be construed as a potential conflict of interest.

## Footnotes

^1^For clarity in this paper, we will reserve the term nitrifier denitrification for this specific chain of enzymatic reactions and will not use it for other forms of reductive N incorporation from ${\text{NO}}_{2}^{-}$ into N_2_O.
