# Supplementary material for: Acidification Enhances Hybrid N2O Production Associated with Aquatic Ammonia-Oxidizing Microorganisms
Source: Front Microbiol. 2017 Jan 9;7:2104. doi: 10.3389/fmicb.2016.02104 (PMC5220105; doi:10.3389/fmicb.2016.02104)
Supplement: Supplementary file 1 [file DataSheet1.DOCX]

**Supplementary Materials for**

**Acidification enhances hybrid N_2_O production associated with aquatic ammonia-oxidizing microorganisms**

Caitlin H. Frame*, Evan Lau, E. Joseph Nolan IV, Tyler J. Goepfert, Moritz F. Lehmann

*Corresponding author. E-mail cframe@alum.mit.edu

**S.1 Evaluating GC-IRMS analyses of ^15^N-enriched N_2_O samples**

Most of the ^15^N in a N_2_O sample with a natural abundance isotopic composition is present in ^15^N^14^N^16^O or ^14^N^15^N^16^O, and a much smaller fraction is present in ^15^N^15^N^16^O. The reference N_2_O gas that was analyzed in parallel with every incubation sample had a natural abundance isotopic composition (δ^15^N = 1.3‰, δ^18^O = 38.1‰ referenced to air N_2_ and VSMOW, respectively)

δ^15^N_reference N2O_ = (^15^R_reference N2O_/^15^R_AIR N2_ -1) * 1000‰,

δ^18^O_reference N2O_ = (^18^R_reference N2O_/^18^R_SMOW_-1) * 1000‰, and

^15^R = [^15^N]/[^14^N],

^18^R = [^18^O]/[^16^O].

The following equations were used to calculate the ^45^N_2_O/^44^N_2_O and ^46^N_2_O/^44^N_2_O of our reference N_2_O, using values of ^15^R, ^18^R, and ^17^R (^17^R = [^17^O]/[^16^O] and ^17^R/0.0003799 = (^18^R/0.0020052)^0.516^ (Kaiser, Rockmann, & Brenninkmeijer, 2003)) derived from the calibrated δ^15^N and δ^18^O values measured for us by Dr. Joachim Mohn (EMPA, Switzerland):

[^45^N_2_O]/[^44^N_2_O] = 2*^15^R + ^17^R

[^46^N_2_O]/[^44^N_2_O] = 2*^15^R*^17^R + ^18^R + ^15^R^2^

For each incubation bottle, the total N_2_O ([N_2_O] units = moles) and the calibrated ratios of the sample [^45^N_2_O]/[^44^N_2_O] and [^46^N_2_O]/[^44^N_2_O] were converted to moles of ^45^N_2_O and ^46^N_2_O:

^45^N_2_O = [N_2_O]*[^45^N_2_O]/[^44^N_2_O]/(1+[^45^N_2_O]/[^44^N_2_O]+[^46^N_2_O]/[^44^N_2_O])

^46^N_2_O = [N_2_O]*[^46^N_2_O]/[^44^N_2_O]/(1+[^45^N_2_O]/[^44^N_2_O]+[^46^N_2_O]/[^44^N_2_O])

The ^15^NH_4_^+^ Lake Lugano incubations produced N_2_O with a ^15^N isotopic enrichment of several hundred ‰, and this ^15^N is present in significant amounts of both m/z ^45^N_2_O (^15^N^14^N^16^O + ^14^N^15^N^16^O) and ^46^N_2_O (^15^N^15^N^16^O). It is potentially problematic to calculate the enrichment in ^15^N^14^N^16^O + ^14^N^15^N^16^O or ^15^N^15^N^16^O using only our reference N_2_O as a calibration point, because any error or uncertainty in the actual or measured isotopic composition of our reference N_2_O is magnified as the isotopic enrichment of the sample increases (measurements are Rsample/Rreference where R is [^45^N_2_O]/[^44^N_2_O] or [^46^N_2_O]/[^44^N_2_O]). It is also possible that responses of the m/z 44, 45, and 46 detectors are not linear over the entire range of m/z ratios in enriched samples, and that any blank associated with purging, trapping, and analyzing the sample N_2_O could change the measured isotope ratios in a way that increases as the isotopic composition of the sample N_2_O diverges from that of the blank.

In order to calibrate our measurements in the absence of accepted N_2_O isotopic standards that bracket the isotopic composition of our incubation N_2_O (1) we mixed quantified amounts ^14^NO_3_^-^ and ^15^NO_3_^-^, reduced this NO_3_^-^ to N_2_O using the denitrifier method (Sigman et al., 2001), and compared the measured [^45^N_2_O]/[^44^N_2_O] and [^46^N_2_O]/[^44^N_2_O] ratios to the expected values assuming a binomial distribution of ^15^N and ^14^N among the N_2_O molecules (Figures S1A and S1B), and (2) we measured samples of isotopically calibrated N_2_O gas mixtures in synthetic air that were provided by Dr. Joachim Mohn (EMPA, Switzerland). These two gases have δ^15^N values that are ^15^N-enriched and ^15^N-depleted, respectively, relative to atmospheric N_2_O (53504 = 48.09‰ and CA-06261 = -35.74‰) and they were analyzed in continuous-flow mode using the same procedures as our incubation N_2_O samples.

For method (1), triplicate solutions of potassium nitrate (KNO_3_) with a known natural abundance isotopic composition and ^15^N-enriched (98.5%) sodium nitrate (Na^15^NO3, Cambridge Isotope Laboratories, NLM-157) were mixed to produce NO_3_^-^ solutions with the ^15^F ([^15^NO_3_^-^]/[^14^NO_3_^-^+^15^NO_3_^-^]) values indicated by the labels next to the data points in Figures S1A and S1B. These solutions were converted to N_2_O using the denitrifier method and then each N_2_O mixture was analyzed by GC-IRMS and plotted on the x-axes of Figure S1A and S1B. The ‘calculated’ ratios plotted on the y-axes are the ratios of [^46^N_2_O]/[^44^N_2_O] and [^45^N_2_O]/[^44^N_2_O] calculated assuming a binomial distribution of ^15^N and ^14^N into the product N_2_O:

S1A S1B

^46^N_2_O / ^44^N_2_O = [^15^NO_3_^-^]^2^ / [^14^NO_3_^-^]^2^

^45^N_2_O / ^44^N_2_O = 2*[^15^NO_3_^-^]*[^14^NO_3_^-^] / [^14^NO_3_^-^]^2^

**Figure S1A and S1B**: comparison of ^46^N_2_O/^44^N_2_O and ^45^N_2_O/^44^N_2_O calculated using the [^14^NO_3_^-^] and [^15^NO_3_^-^] and assuming a binomial distribution of ^14^N and ^15^N in N_2_O during the denitrifier method to values measured using the calibrated GC-IRMS analyses. Labels next to the data points indicate the ^15^F_NO3-_. For each value of ^15^F_NO3-_, N_2_O measurements were made in triplicate or quadruplicate.

Assuming that biological isotopic discrimination does not influence the rates at which ^14^NO + ^14^NO, ^15^NO + ^14^NO, and ^15^NO + ^15^NO react with each other, then the closer the slopes of the lines in Figures S1A and S1B are to 1, the better our GC-IRMS calibration and measurement procedure is at reproducing the actual ratios of [^46^N_2_O]/[^44^N_2_O] and [^45^N_2_O]/[^44^N_2_O]. Indeed, the slope of the ^46^N_2_O/^44^N_2_O plot (0.99514) is very close to 1 (Figure S1A), indicating that our GC-IRMS calibration and measurement procedure reproduces the actual ratios of [^46^N_2_O]/[^44^N_2_O] very well. The slope of the [^45^N_2_O]/[^44^N_2_O] plot is 1.0549, which indicates a ~5% scale compression of the measured values relative to the ‘actual’ values. Using method (2) discrete continuous flow GC-IRMS measurements of N_2_O mixtures 53504 and CA-06261 were made 12 and 13 times, respectively. Their [^45^N_2_O]/[^44^N_2_O] values were also calculated using their calibrated δ^15^N and δ^18^O values and compared to the corresponding calibrated measurements of the gases (Figure S2). The corresponding linear regression had a slope of 1.0534, which is quite similar to the values obtained using method (1), and therefore suggests that the scale compression is due to instrumental biasing effects and not biological isotope effects in method (1). Therefore, we applied a scale-compression correction (5.49%) to all of the incubation [^45^N_2_O]/[^44^N_2_O] measurements.

S2

**Figure S2**: comparison of [^45^N_2_O]/[^44^N_2_O] calculated using the calibrated δ^15^N and δ^18^O values of gas mixtures CA-06261 and 53504 and the values measured via continuous flow methods on the GC-IRMS.

**S.2 Relating ^15^N^15^N^16^O and ^14^N^15^N^16^O + ^15^N^14^N^16^O production to δ^18^O-N_2_O and δ^15^N-N_2_O**

During the ^15^NH_4_^+^ incubations presented here, production of ^14^N^15^N^16^O and ^15^N^14^N^16^O produces corresponding increases in the measured δ^15^N-N_2_O because the calculation of ^15^R for samples with natural abundance isotopic compositions depends mainly on [^45^N_2_O]/[^44^N_2_O] (see equations above). Similarly, production of ^15^N^15^N^16^O increases the reported δ^18^O-N_2_O because the corresponding calculation of ^18^R for N_2_O with a natural abundance isotopic composition depends mainly on [^46^N_2_O]/[^44^N_2_O], which increases in proportion to both [^14^N^14^N^18^O] and [^15^N^15^N^16^O].

The Namibian Upwelling incubations with ^15^NH_4_^+^ produced enrichments of δ^15^N-N_2_O (see Table 2 in the main text), but the enrichments were not high enough to calculate significant N_2_O productions rates, as we did for the Lake Lugano incubation data. Nevertheless, we wanted to know whether the observed increases in δ^15^N-N_2_O corresponded to hybrid N_2_O production (i.e. one NH_4_^+^-derived N and one N derived from an unlabeled N pool such as NO_2_^-^), which would mainly produce ^45^N_2_O, or whether they corresponded to production of N_2_O solely from NH_4_^+^-derived N (i.e. N_2_O with a binomial distribution of the ^15^N and ^14^N in the substrate NH_4_^+^), which would produce ^45^N_2_O and ^46^N_2_O as a function of ^15^F__NH4+_. In the case of hybrid N_2_O formation, we would expect to see a very small increase in the reported δ^18^O-N_2_O relative to the change in δ^15^N-N_2_O, while in the second case, we would expect a much larger increase in the apparent δ^18^O-N_2_O relative to the increase in δ ^15^N-N_2_O.

In Figure S3, we have calculated the relationship between the increase in the reported δ^15^N-N_2_O (x-axis) and δ^18^O-N_2_O (y-axis), assuming a binomial distribution of ^14^N and ^15^N from NH_4_^+^ in the produced N_2_O, and that the relative rate of ionization of ^15^N^15^N^16^O and ^14^N^14^N^18^O by the source of the mass spectrometer is 1:1. Each curve corresponds to a different ratio of the amount of N_2_O produced during the incubation to the amount of background natural abundance N_2_O present at the start of the incubation. The black circles correspond to the average changes in the measured δ^15^N-N_2_O and δ^18^O-N_2_O over the 24-hour incubation period for the three sets of O_2_ concentrations tested during the Namibian Upwelling incubations (Table 2, main text). These values fall closest to the portions of the curves where ^15^F__NH4+_ < 20%. However, we know that the initial atom fraction of NH_4_^+^ (^15^F__NH4+0_) during these incubations was 0.94 (94%). Although we did not measure the change in ^15^F__NH4+_ over the 24-hour incubation period, given the high %^15^N and initial concentration (1 μM) of the added tracer ^15^NH_4_^+^, it seems unlikely that the average ^15^F__NH4+_ dropped to < 20% over this period. For this reason, we conclude that the N_2_O production mechanism observed during the experiments was a hybrid one.

**Figure S3**: Expected changes in the reported δ^18^O-N_2_O and δ^15^N-N_2_O during ^15^NH_4_^+^ incubations if N_2_O is produced solely from a binomial distribution of NH_4_^+^-derived N. These changes are a function of the ^15^F of the substrate NH_4_^+^ and the ratio of N_2_O produced to preexisiting/background N_2_O. Black circles represent incubation results from the Namibian Upwelling experiments. The solid black line corresponds to the increase of measured δ^15^N-N_2_O and δ^18^O-N_2_O expected if the ^15^F_NH4+_ = 0.94.

S3

­­


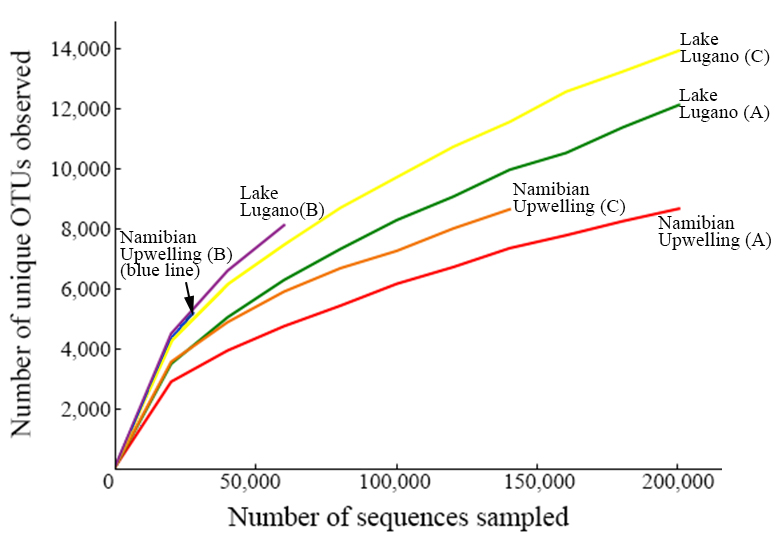
**Figure S4:** Microbial taxon richness in Namibian Upwelling (A-C) and Lake Lugano (A-C) sequencing reactions, assessed by rarefaction curves. The rarefaction curves show the relationship between increasing the number of random samplings and new, unique microbial OTUs in order to assess species richness from the results of sampling.

.


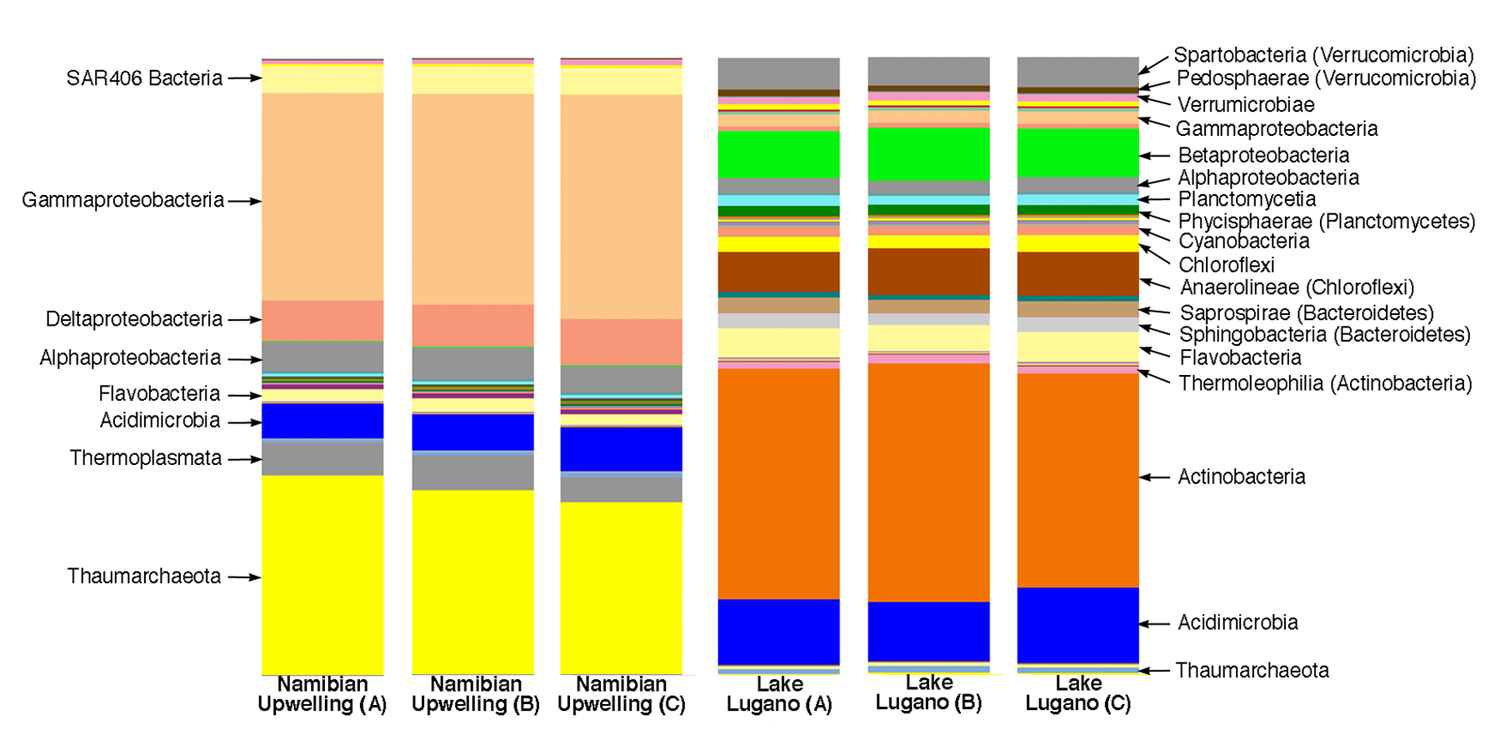


**Figure S5:** Relative sequence abundances of bacterial phyla and candidate archaeal divisions based on the 271,425 unique OTU reads obtained from triplicate reactions (A, B, C) performed with DNA extracted from either the Namibian Upwelling or the Lake Lugano incubation water.

**S.3 Statistical Analysis of Putative AOB and AOA OTUs**

The Shannon-Weiner diversity index (H) is directly proportional to the number of taxa and inversely proportional to the number of sequences falling into each taxon. AOB-sequence abundances in Lake Lugano were significantly higher than AOB-sequence abundances in the Namibian Upwelling (see Table S1, Table S2), and the H values for AOB an order of magnitude higher in Lake Lugano than the Namibian Upwelling (Figure S6). In the Namibian Upwelling sample, where the abundance of AOA sequences is significantly higher than that of AOA sequences in Lake Lugano, the H value for AOA sequences was also higher (Figure S6). The evenness value (E) is inversely proportional to the number of individuals per taxon, with a higher evenness value indicating a more uneven taxa distribution. Evenness among AOA sequences was higher (i.e., less even) in the Namibian Upwelling sample than in the Lake Lugano sample (Figure S6).

S6

**
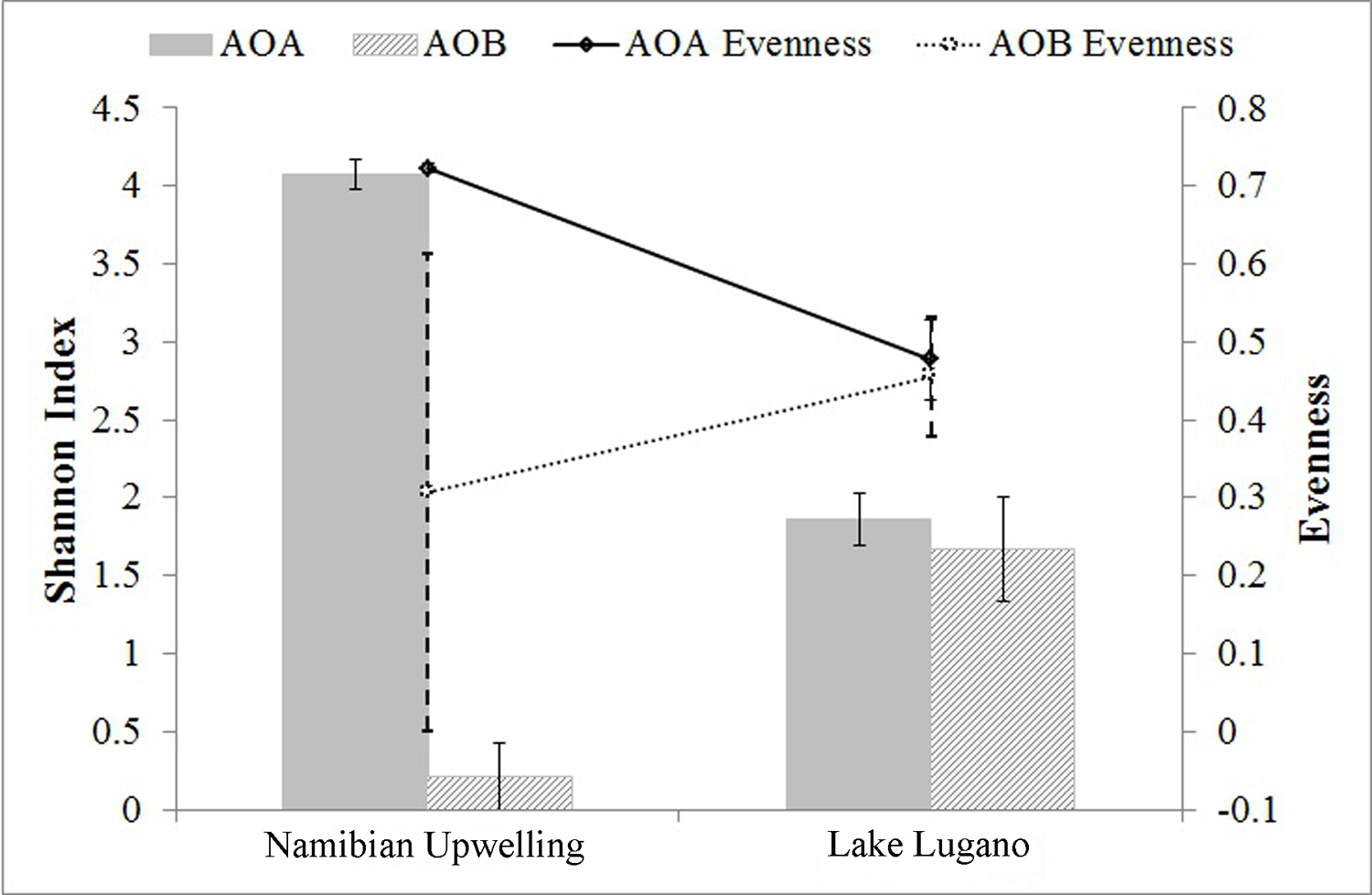
**

**Figure S6:** Calculated Shannon-Weiner and evenness indices for AOA- and AOB-related OTUs for samples from the Namibian Upwelling and Lake Lugano. Standard errors are represented by error bars for Shannon-Weiner and evenness indices.

**Table S1.** 16S rRNA gene sequences of 65 OTUs, which are related to the ammonium-oxidizing Bacteria (AOB), their accession numbers and % nucleotide identity with their closest known cultured relatives. The number of times these sequences were detected in each sample are indicated. NU= Namibian Upwelling, Lu=Lake Lugano

| **OTU** | **GenBank Accession no.** | **Closest Cultured Nitrifier Relative (GenBank Accession no.)** | **% Identity** | **NU(A)** | **NU (B)** | **NU (C)** | **Lu (A)** | **Lu (B)** | **Lu (C)** |
| --- | --- | --- | --- | --- | --- | --- | --- | --- | --- |
| **770** | LN908721 | *Nitrosospira briensis* (AY123800.1) | 100.0 | 0 | 0 | 1 | 474 | 6 | 36 |
| **1380** | LN908722 | *Nitrosospira briensis* C-128 (CP012371.1) | 99.0 | 0 | 0 | 0 | 12 | 1 | 1 |
| **3249** | LN908723 | *Nitrosospira briensis* C-128 (CP012371.1) | 95.0 | 1 | 0 | 0 | 6 | 0 | 3 |
| **7496** | LN908724 | *Nitrosospira multiformis* strain ATCC 25196 (NR_074736.1) | 94.0 | 0 | 0 | 2 | 0 | 0 | 3 |
| **8380** | LN908725 | *Nitrosomonas* sp. Is79A3 (CP002876.1) | 97.0 | 0 | 0 | 0 | 3 | 0 | 1 |
| **8510** | LN908726 | *Nitrosospira briensis* C-128 (CP012371.1) | 98.0 | 0 | 0 | 0 | 46 | 7 | 37 |
| **8787** | LN908727 | *Nitrosospira tenuis* strain Nv1 (NR_114773.1) | 97.0 | 0 | 0 | 0 | 1 | 0 | 0 |
| **8958** | LN908728 | *Nitrosospira* sp. Nl20 (AJ298729.1) and *Nitrosospira* *tenuis* strain Nv1 (NR_114773.1) | 98.0 | 0 | 0 | 0 | 2 | 0 | 1 |
| **9364** | LN908729 | *Nitrosospira* sp. Nl20 (AJ298729.1) and *Nitrosospira* *tenuis* strain Nv1 (NR_114773.1) | 97.0 | 0 | 0 | 0 | 7 | 2 | 1 |
| **9663** | LN908730 | *Nitrosospira* sp. Nl5 (AY123812.1) | 94.0 | 0 | 0 | 0 | 1 | 1 | 0 |
| **9812** | LN908731 | *Nitrosospira briensis* (AY123800.1) | 94.0 | 0 | 0 | 0 | 1 | 0 | 0 |
| **9840** | LN908732 | *Nitrosospira* sp. isolate D11 ( X84660.1) | 93.0 | 0 | 0 | 0 | 1 | 0 | 0 |
| **9842** | LN908733 | *Nitrosospira briensis* (AY123800.1) | 99.0 | 0 | 0 | 0 | 9 | 2 | 11 |
| **10163** | LN908734 | *Nitrosospira* sp. Nl20 (AJ298729.1) and *Nitrosospira* *tenuis* strain Nv1 (NR_114773.1) | 95.0 | 0 | 0 | 0 | 1 | 1 | 0 |
| **10363** | LN908735 | *Nitrosospira* sp. Nl20 (AJ298729.1) and *Nitrosospira* *tenuis* strain Nv1 (NR_114773.1) | 96.0 | 0 | 0 | 0 | 1 | 0 | 1 |
| **10451** | LN908736 | *Nitrosospira briensis* (AY123800.1) | 99.0 | 0 | 0 | 0 | 52 | 1 | 4 |
| **10502** | LN908737 | *Nitrosospira briensis* (AY123800.1) | 99.0 | 0 | 0 | 0 | 321 | 4 | 6 |
| **10573** | LN908738 | *Nitrosospira briensis* (AY123800.1) | 94.0 | 0 | 0 | 0 | 1 | 0 | 1 |
| **10637** | LN908739 | *Nitrosospira briensis* (AY123800.1) | 100.0 | 0 | 0 | 0 | 670 | 20 | 62 |
| **11405** | LN908740 | *Nitrosospira* sp. Nl20 (AJ298729.1) | 94.0 | 0 | 0 | 0 | 1 | 1 | 0 |
| **11475** | LN908741 | *Nitrosospira* sp. Nl20 (AJ298729.1) and *Nitrosospira* *tenuis* strain Nv1 (NR_114773.1) | 92.0 | 0 | 0 | 0 | 2 | 1 | 0 |
| **11652** | LN908742 | *Nitrosospira briensis* (AY123800.1) | 96.0 | 0 | 0 | 0 | 1 | 0 | 0 |
| **11829** | LN908743 | *Nitrosospira* sp. Nl20 (AJ298729.1) and *Nitrosospira* *tenuis* strain Nv1 (NR_114773.1) | 92.0 | 0 | 0 | 0 | 4 | 0 | 0 |
| **11906** | LN908744 | *Nitrosospira* sp. Nv6 (AY123805) | 98.0 | 0 | 0 | 0 | 3 | 2 | 4 |
| **12129** | LN908745 | *Nitrosospira* sp. Nl20 (AJ298729.1) and *Nitrosospira* *tenuis* strain Nv1 (NR_114773.1) | 94.0 | 0 | 0 | 0 | 1 | 0 | 0 |
| **12691** | LN908746 | *Nitrosospira* sp. isolate D11 ( X84660.1) | 97.0 | 0 | 0 | 0 | 1 | 0 | 0 |
| **12969** | LN908747 | *Nitrosospira briensis* (AY123800.1) | 93.0 | 0 | 0 | 0 | 1 | 1 | 1 |
| **13024** | LN908748 | *Nitrosospira briensis* (AY123800.1) | 94.0 | 0 | 0 | 0 | 3 | 1 | 0 |
| **13037** | LN908749 | *Nitrosospira briensis* C-128 (CP012371.1) | 94.0 | 0 | 0 | 0 | 1 | 0 | 0 |
| **13154** | LN908750 | *Nitrosospira briensis* (AY123800.1) | 98.0 | 0 | 0 | 0 | 2 | 2 | 2 |
| **13357** | LN908751 | *Nitrosospira briensis* (AY123800.1) | 100.0 | 0 | 0 | 0 | 597 | 331 | 90 |
| **13617** | LN908752 | Nitrosomonas ureae strain Nm10 (NR_104814.1) | 95.0 | 0 | 0 | 0 | 1 | 0 | 0 |
| **13997** | LN908753 | *Nitrosospira briensis* C-128 (CP012371.1) | 92.0 | 0 | 0 | 0 | 2 | 0 | 0 |
| **14287** | LN908754 | *Nitrosomonas oligotropha* strain Nm45 (NR_104820.1) | 98.0 | 0 | 0 | 0 | 1 | 0 | 0 |
| **14437** | LN908755 | *Nitrosospira briensis* (AY123800.1) | 99.0 | 0 | 0 | 0 | 1 | 12 | 408 |
| **14515** | LN908756 | Nitrosomonas sp. Is79A3 (CP002876.1) | 99.0 | 0 | 0 | 0 | 1 | 1 | 6 |
| **14586** | LN908757 | *Nitrosospira briensis* (AY123800.1) | 95.0 | 0 | 0 | 0 | 1 | 0 | 0 |
| **14738** | LN908758 | *Nitrosospira* sp. Nl20 (AJ298729.1) and *Nitrosospira* *tenuis* strain Nv1 (NR_114773.1) | 92.0 | 0 | 0 | 0 | 1 | 0 | 1 |
| **14740** | LN908759 | *Nitrosospira briensis* (AY123800.1) | 95.0 | 0 | 0 | 0 | 1 | 0 | 2 |
| **15135** | LN908760 | *Nitrosospira briensis* C-128 (CP012371.1) | 96.0 | 0 | 0 | 0 | 0 | 1 | 0 |
| **15544** | LN908761 | *Nitrosospira briensis* (AY123800.1) | 94.0 | 0 | 0 | 0 | 0 | 1 | 0 |
| **15549** | LN908762 | *Nitrosospira briensis* (AY123800.1) | 99.0 | 0 | 0 | 0 | 1 | 2 | 46 |
| **15587** | LN908763 | *Nitrosospira briensis* C-128 (CP012371.1) | 98.0 | 0 | 0 | 0 | 0 | 1 | 2 |
| **15765** | LN908764 | *Nitrosospira briensis* (AY123800.1) | 100.0 | 0 | 0 | 0 | 0 | 7 | 436 |
| **15821** | LN908765 | *Nitrosospira briensis* (AY123800.1) | 99.0 | 0 | 0 | 0 | 0 | 1 | 2 |
| **15926** | LN908766 | *Nitrosospira briensis* C-128 (CP012371.1) | 99.0 | 0 | 0 | 0 | 0 | 3 | 99 |
| **15971** | LN908767 | *Nitrosospira briensis* (AY123800.1) | 99.0 | 0 | 0 | 0 | 0 | 2 | 22 |
| **16577** | LN908768 | *Nitrosospira* sp. Nl20 (AJ298729.1) and *Nitrosospira* *tenuis* strain Nv1 (NR_114773.1) | 99.0 | 0 | 0 | 0 | 0 | 0 | 5 |
| **16622** | LN908769 | *Nitrosospira briensis* (AY123800.1) | 95.0 | 0 | 0 | 0 | 0 | 0 | 1 |
| **16833** | LN908770 | *Nitrosospira briensis* C-128 (CP012371.1) | 96.0 | 0 | 0 | 0 | 0 | 0 | 1 |
| **17058** | LN908771 | *Nitrosospira briensis* C-128 (CP012371.1) | 99.0 | 0 | 0 | 0 | 0 | 0 | 72 |
| **17089** | LN908772 | *Nitrosospira briensis* C-128 (CP012371.1) | 97.0 | 0 | 0 | 0 | 0 | 0 | 1 |
| **17142** | LN908773 | *Nitrosospira* sp. Nl20 (AJ298729.1) and *Nitrosospira* *tenuis* strain Nv1 (NR_114773.1) | 98.0 | 0 | 0 | 0 | 0 | 0 | 6 |
| **17377** | LN908774 | *Nitrosospira* sp. Nl20 (AJ298729.1) and *Nitrosospira* *tenuis* strain Nv1 (NR_114773.1) | 93.0 | 0 | 0 | 0 | 0 | 0 | 1 |
| **17824** | LN908775 | *Nitrosospira briensis* (AY123800.1) | 99.0 | 0 | 0 | 0 | 3 | 1 | 111 |
| **17943** | LN908776 | *Nitrosospira briensis* (AY123800.1) | 97.0 | 0 | 0 | 0 | 0 | 0 | 3 |
| **18051** | LN908777 | *Nitrosospira briensis* (AY123800.1) | 91.0 | 0 | 0 | 0 | 0 | 0 | 2 |
| **18136** | LN908778 | *Nitrosospira briensis* (AY123800.1) | 99.0 | 0 | 0 | 0 | 0 | 0 | 5 |
| **18341** | LN908779 | *Nitrosospira briensis* (AY123800.1) | 99.0 | 0 | 0 | 0 | 0 | 0 | 1 |
| **18468** | LN908780 | *Nitrosospira* sp. Nl20 (AJ298729.1) and *Nitrosospira* *tenuis* strain Nv1 (NR_114773.1) | 97.0 | 0 | 0 | 0 | 0 | 0 | 1 |
| **18734** | LN908781 | *Nitrosospira* sp. isolate D11 ( X84660.1 | 94.0 | 0 | 0 | 0 | 0 | 0 | 4 |
| **18870** | LN908782 | *Nitrosospira briensis* (AY123800.1) | 98.0 | 0 | 0 | 0 | 0 | 0 | 1 |
| **18932** | LN908783 | *Nitrosospira briensis* C-128 (CP012371.1) | 92.0 | 0 | 0 | 0 | 0 | 0 | 1 |
| **18937** | LN908784 | *Nitrosospira* sp. Nl5 (AJ23812.1) | 93.0 | 0 | 0 | 0 | 0 | 0 | 1 |
| **19851** | LN908785 | *Nitrosospira* sp. Nl20 (AJ298729.1) and *Nitrosospira* *tenuis* strain Nv1 (NR_114773.1) | 94.0 | 0 | 0 | 0 | 1 | 0 | 0 |

**Table S2.** 16S rRNA gene sequences of 442 OTUs which are related to the ammonium-oxidizing Archaea (AOA), their accession numbers and % nucleotide identity with their closest known cultured relatives. The number of times these sequences were detected in each sample are indicated. NU = Namibian Upwelling, Lu=Lake Lugano

| **OTU** | **GenBank Accession no.** | **Closest Cultured Nitrifier Relative (GenBank Accession no.)** | **% Identity** | **NU (A)** | **NU (B)** | **NU**  **(C)** | **Lu**  **(A)** | **Lu**  **(B)** | **Lu**  **(C)** |
| --- | --- | --- | --- | --- | --- | --- | --- | --- | --- |
| 33 | LN908279 | Candidatus Nitrosopelagicus brevis strain CN25 (CP007026.1) | 97.0 | 2 | 0 | 0 | 0 | 0 | 2 |
| 186 | LN908280 | Candidatus Nitrosopelagicus brevis strain CN25 (CP007026.1) | 97.0 | 1362 | 76 | 216 | 0 | 0 | 0 |
| 204 | LN908281 | Candidatus Nitrosopelagicus brevis strain CN25 (CP007026.1) | 97.0 | 10 | 0 | 0 | 0 | 0 | 0 |
| 274 | LN908282 | Candidatus Nitrosopumilus sp. NF5 (CP011070.1) and Candidatus Nitrosopumilus sp. D3C (CP010868.1) | 97.0 | 9058 | 723 | 4962 | 4 | 3 | 7 |
| 303 | LN908283 | Candidatus Nitrosopelagicus brevis strain CN25 (CP007026.1) | 96.0 | 1060 | 29 | 186 | 0 | 0 | 0 |
| 348 | LN908284 | Candidatus Nitrosopumilus sp. NF5 (CP011070.1) and Candidatus Nitrosopumilus sp. D3C (CP010868.1) | 96.0 | 4798 | 343 | 2385 | 3 | 1 | 4 |
| 401 | LN908285 | Candidatus Nitrosopelagicus brevis strain CN25 (CP007026.1) | 94.0 | 817 | 62 | 356 | 1 | 0 | 0 |
| 410 | LN908286 | Candidatus Nitrosopumilus sp. HCA1 (KF957663.1) | 98.0 | 37 | 8 | 13 | 3 | 2 | 3 |
| 443 | LN908287 | Candidatus Nitrosopelagicus brevis strain CN25 (CP007026.1) | 92.0 | 1 | 0 | 0 | 0 | 0 | 0 |
| 556 | LN908288 | Candidatus Nitrosopelagicus brevis strain CN25 (CP007026.1) | 92.0 | 379 | 22 | 70 | 0 | 0 | 0 |
| 563 | LN908289 | Candidatus Nitrosopumilus sp. NF5 (CP011070.1) and Candidatus Nitrosopumilus sp. D3C (CP010868.1) | 96.0 | 442 | 33 | 117 | 0 | 0 | 1 |
| 571 | LN908290 | Candidatus Nitrosopelagicus brevis strain CN25 (CP007026.1) | 98.0 | 1707 | 120 | 816 | 0 | 0 | 1 |
| 1686 | LN908291 | Candidatus Nitrosopelagicus brevis strain CN25 (CP007026.1) | 98.0 | 0 | 0 | 0 | 1 | 0 | 1 |
| 1699 | LN908292 | Candidatus Nitrosopelagicus brevis strain CN25 (CP007026.1) | 98.0 | 1444 | 84 | 530 | 2 | 0 | 4 |
| 1739 | LN908293 | Candidatus Nitrosopelagicus brevis strain CN25 (CP007026.1) | 98.0 | 1580 | 91 | 915 | 2 | 1 | 1 |
| 1746 | LN908294 | Candidatus Nitrosopelagicus brevis strain CN25 (CP007026.1) | 95.0 | 129 | 6 | 30 | 0 | 0 | 0 |
| 1766 | LN908295 | Candidatus Nitrosopelagicus brevis strain CN25 (CP007026.1) | 96.0 | 65 | 2 | 15 | 0 | 0 | 0 |
| 1838 | LN908296 | Candidatus Nitrosopelagicus brevis strain CN25 (CP007026.1) | 97.0 | 72 | 0 | 5 | 0 | 0 | 0 |
| 1892 | LN908297 | Candidatus Nitrosopelagicus brevis strain CN25 (CP007026.1) | 96.0 | 202 | 6 | 58 | 0 | 0 | 0 |
| 1903 | LN908298 | Candidatus Nitrosopumilus sp. NF5 (CP011070.1) and Candidatus Nitrosopumilus sp. D3C (CP010868.1) | 95.0 | 201 | 4 | 41 | 0 | 0 | 0 |
| 1905 | LN908299 | Candidatus Nitrosopelagicus brevis strain CN25 (CP007026.1) | 96.0 | 325 | 4 | 31 | 0 | 0 | 0 |
| 1927 | LN908300 | Candidatus Nitrosopelagicus brevis strain CN25 (CP007026.1) | 96.0 | 161 | 3 | 47 | 0 | 0 | 0 |
| 1938 | LN908301 | Candidatus Nitrosopelagicus brevis strain CN25 (CP007026.1) | 96.0 | 43 | 2 | 34 | 0 | 0 | 0 |
| 1983 | LN908302 | Candidatus Nitrosopelagicus brevis strain CN25 (CP007026.1) | 95.0 | 94 | 5 | 27 | 0 | 0 | 0 |
| 1995 | LN908303 | Candidatus Nitrosopelagicus brevis strain CN25 (CP007026.1) | 96.0 | 136 | 3 | 6 | 0 | 0 | 0 |
| 2034 | LN908304 | Candidatus Nitrosopelagicus brevis strain CN25 (CP007026.1) | 95.0 | 65 | 0 | 6 | 0 | 0 | 0 |
| 2041 | LN908305 | Candidatus Nitrosopelagicus brevis strain CN25 (CP007026.1) | 94.0 | 73 | 8 | 38 | 0 | 0 | 0 |
| 2054 | LN908306 | Candidatus Nitrosopelagicus brevis strain CN25 (CP007026.1) | 94.0 | 600 | 32 | 227 | 0 | 0 | 1 |
| 2084 | LN908307 | Candidatus Nitrosopelagicus brevis strain CN25 (CP007026.1) | 95.0 | 66 | 3 | 29 | 0 | 0 | 0 |
| 2089 | LN908308 | Candidatus Nitrosopelagicus brevis strain CN25 (CP007026.1) | 95.0 | 25 | 5 | 6 | 1 | 0 | 1 |
| 2091 | LN908309 | Candidatus Nitrosopelagicus brevis strain CN25 (CP007026.1) | 93.0 | 98 | 4 | 31 | 0 | 0 | 0 |
| 2105 | LN908310 | Candidatus Nitrosopumilus sp. NF5 (CP011070.1) and Candidatus Nitrosopumilus sp. D3C (CP010868.1) | 95.0 | 32 | 0 | 2 | 0 | 0 | 0 |
| 2128 | LN908311 | Candidatus Nitrosopelagicus brevis strain CN25 (CP007026.1) | 93.0 | 2 | 0 | 0 | 0 | 0 | 0 |
| 2147 | LN908312 | Candidatus Nitrosopelagicus brevis strain CN25 (CP007026.1) | 96.0 | 125 | 4 | 20 | 0 | 0 | 0 |
| 2149 | LN908313 | Candidatus Nitrosopelagicus brevis strain CN25 (CP007026.1) | 96.0 | 704 | 12 | 44 | 0 | 0 | 0 |
| 2165 | LN908314 | Candidatus Nitrosopelagicus brevis strain CN25 (CP007026.1) | 96.0 | 1 | 0 | 0 | 0 | 0 | 0 |
| 2166 | LN908315 | Candidatus Nitrosopumilus sp. HCA1 (KF957663.1) | 99.0 | 1 | 0 | 0 | 0 | 0 | 0 |
| 2167 | LN908316 | Candidatus Nitrosopumilus sp. NF5 (CP011070.1) and Candidatus Nitrosopumilus sp. D3C (CP010868.1) | 97.0 | 180 | 9 | 11 | 0 | 0 | 0 |
| 2171 | LN908317 | Candidatus Nitrosopelagicus brevis strain CN25 (CP007026.1) | 95.0 | 441 | 6 | 15 | 0 | 0 | 0 |
| 2200 | LN908318 | Candidatus Nitrosopumilus sp. NF5 (CP011070.1) and Candidatus Nitrosopumilus sp. D3C (CP010868.1) | 96.0 | 71 | 0 | 7 | 0 | 0 | 1 |
| 2208 | LN908319 | Candidatus Nitrosopelagicus brevis strain CN25 (CP007026.1) | 93.0 | 154 | 3 | 5 | 0 | 0 | 0 |
| 2231 | LN908320 | Candidatus Nitrosopumilus sp. HCA1 (KF957663.1) | 99.0 | 271 | 40 | 49 | 0 | 0 | 0 |
| 2258 | LN908321 | Candidatus Nitrosopumilus sp. NF5 (CP011070.1) and Candidatus Nitrosopumilus sp. D3C (CP010868.1) | 94.0 | 121 | 12 | 54 | 0 | 0 | 0 |
| 2274 | LN908322 | Candidatus Nitrosopelagicus brevis strain CN25 (CP007026.1) | 95.0 | 438 | 22 | 48 | 0 | 0 | 0 |
| 2278 | LN908323 | Candidatus Nitrosopelagicus brevis strain CN25 (CP007026.1) | 95.0 | 82 | 2 | 21 | 0 | 0 | 0 |
| 2283 | LN908324 | Candidatus Nitrosopumilus sp. NF5 (CP011070.1) and Candidatus Nitrosopumilus sp. D3C (CP010868.1) | 94.0 | 2 | 0 | 0 | 0 | 0 | 0 |
| 2284 | LN908325 | Candidatus Nitrosopumilus sp. NF5 (CP011070.1) and Candidatus Nitrosopumilus sp. D3C (CP010868.1) | 98.0 | 77 | 7 | 19 | 0 | 0 | 0 |
| 2295 | LN908326 | Candidatus Nitrosopelagicus brevis strain CN25 (CP007026.1) | 96.0 | 538 | 4 | 4 | 0 | 0 | 0 |
| 2307 | LN908327 | Candidatus Nitrosopelagicus brevis strain CN25 (CP007026.1) | 95.0 | 35 | 3 | 7 | 0 | 0 | 0 |
| 2313 | LN908328 | Candidatus Nitrosopelagicus brevis strain CN25 (CP007026.1) | 97.0 | 182 | 9 | 28 | 0 | 1 | 0 |
| 2339 | LN908329 | Candidatus Nitrosopelagicus brevis strain CN25 (CP007026.1) | 97.0 | 337 | 21 | 142 | 0 | 0 | 1 |
| 2365 | LN908330 | Candidatus Nitrosopelagicus brevis strain CN25 (CP007026.1) | 97.0 | 67 | 4 | 17 | 0 | 0 | 0 |
| 2375 | LN908331 | Candidatus Nitrosopumilus sp. HCA1 (KF957663.1) | 99.0 | 622 | 73 | 152 | 19 | 0 | 0 |
| 2394 | LN908332 | Candidatus Nitrosopelagicus brevis strain CN25 (CP007026.1) | 96.0 | 78 | 1 | 0 | 0 | 0 | 0 |
| 2412 | LN908333 | Candidatus Nitrosopelagicus brevis strain CN25 (CP007026.1) | 95.0 | 101 | 0 | 9 | 0 | 0 | 0 |
| 2424 | LN908334 | Candidatus Nitrosopelagicus brevis strain CN25 (CP007026.1) | 95.0 | 107 | 5 | 13 | 0 | 0 | 0 |
| 2446 | LN908335 | Candidatus Nitrosopelagicus brevis strain CN25 (CP007026.1) | 95.0 | 170 | 10 | 87 | 0 | 0 | 0 |
| 2492 | LN908336 | Candidatus Nitrosopelagicus brevis strain CN25 (CP007026.1) | 96.0 | 14 | 0 | 0 | 1 | 0 | 0 |
| 2497 | LN908337 | Candidatus Nitrosopumilus sp. NM25 (AB546961.1) | 94.0 | 46 | 1 | 2 | 0 | 0 | 0 |
| 2507 | LN908338 | Candidatus Nitrosopelagicus brevis strain CN25 (CP007026.1) | 96.0 | 169 | 1 | 10 | 1 | 0 | 0 |
| 2538 | LN908339 | Candidatus Nitrosopumilus sp. HCA1 (KF957663.1) | 98.0 | 332 | 0 | 2 | 0 | 0 | 0 |
| 2545 | LN908340 | Candidatus Nitrosopumilus sp. NF5 (CP011070.1) and Candidatus Nitrosopumilus sp. D3C (CP010868.1) | 96.0 | 13 | 0 | 1 | 0 | 0 | 0 |
| 2574 | LN908341 | Candidatus Nitrosopelagicus brevis strain CN25 (CP007026.1) | 98.0 | 636 | 11 | 52 | 0 | 0 | 0 |
| 2613 | LN908342 | Candidatus Nitrosopelagicus brevis strain CN25 (CP007026.1) | 95.0 | 158 | 7 | 29 | 0 | 0 | 0 |
| 2629 | LN908343 | Candidatus Nitrosopumilus sp. NF5 (CP011070.1) and Candidatus Nitrosopumilus sp. D3C (CP010868.1) | 90.0 | 4 | 0 | 0 | 0 | 0 | 0 |
| 2638 | LN908344 | Candidatus Nitrosopumilus sp. NF5 (CP011070.1) and Candidatus Nitrosopumilus sp. D3C (CP010868.1) | 89.0 | 5 | 0 | 0 | 0 | 0 | 0 |
| 2662 | LN908345 | Candidatus Nitrosopumilus sp. NF5 (CP011070.1) and Candidatus Nitrosopumilus sp. D3C (CP010868.1) | 96.0 | 334 | 24 | 206 | 0 | 0 | 0 |
| 2682 | LN908346 | Candidatus Nitrosopumilus sp. NF5 (CP011070.1) and Candidatus Nitrosopumilus sp. D3C (CP010868.1) | 95.0 | 3 | 0 | 1 | 0 | 0 | 0 |
| 2696 | LN908347 | Candidatus Nitrosopelagicus brevis strain CN25 (CP007026.1) | 98.0 | 51 | 4 | 19 | 0 | 0 | 0 |
| 2712 | LN908348 | Candidatus Nitrosopelagicus brevis strain CN25 (CP007026.1) | 98.0 | 1316 | 98 | 459 | 0 | 0 | 1 |
| 2720 | LN908349 | Candidatus Nitrosopelagicus brevis strain CN25 (CP007026.1) | 95.0 | 1615 | 146 | 323 | 0 | 0 | 1 |
| 2726 | LN908350 | Candidatus Nitrosopelagicus brevis strain CN25 (CP007026.1) | 95.0 | 450 | 19 | 64 | 0 | 0 | 0 |
| 2742 | LN908351 | Candidatus Nitrosopelagicus brevis strain CN25 (CP007026.1) | 95.0 | 457 | 31 | 89 | 0 | 0 | 1 |
| 2744 | LN908352 | Candidatus Nitrosopelagicus brevis strain CN25 (CP007026.1) | 96.0 | 417 | 14 | 75 | 0 | 0 | 0 |
| 2772 | LN908353 | Candidatus Nitrosopumilus sp. NF5 (CP011070.1) and Candidatus Nitrosopumilus sp. D3C (CP010868.1) | 97.0 | 5 | 0 | 2 | 0 | 0 | 0 |
| 2791 | LN908354 | Candidatus Nitrosopelagicus brevis strain CN25 (CP007026.1) | 96.0 | 32 | 1 | 14 | 0 | 0 | 0 |
| 2794 | LN908355 | Candidatus Nitrosopelagicus brevis strain CN25 (CP007026.1) | 96.0 | 57 | 0 | 0 | 0 | 0 | 0 |
| 2816 | LN908356 | Candidatus Nitrosopelagicus brevis strain CN25 (CP007026.1 | 96.0 | 49 | 1 | 13 | 0 | 0 | 0 |
| 2823 | LN908357 | Candidatus Nitrosopumilus sp. HCA1 (KF957663.1) | 100.0 | 175 | 8 | 12 | 0 | 0 | 0 |
| 2830 | LN908358 | Candidatus Nitrosopelagicus brevis strain CN25 (CP007026.1) | 93.0 | 1 | 1 | 1 | 0 | 0 | 0 |
| 2858 | LN908359 | Candidatus Nitrosopelagicus brevis strain CN25 (CP007026.1) | 96.0 | 1 | 0 | 0 | 0 | 0 | 0 |
| 2866 | LN908360 | Candidatus Nitrosopelagicus brevis strain CN25 (CP007026.1) | 98.0 | 676 | 12 | 57 | 0 | 0 | 0 |
| 2869 | LN908361 | Candidatus Nitrosopelagicus brevis strain CN25 (CP007026.1) | 96.0 | 9 | 0 | 3 | 0 | 0 | 0 |
| 2871 | LN908362 | Candidatus Nitrosopumilus sp. HCA1 (KF957663.1) | 93.0 | 1 | 0 | 1 | 0 | 0 | 0 |
| 2878 | LN908363 | Candidatus Nitrosopelagicus brevis strain CN25 (CP007026.1) | 96.0 | 1080 | 50 | 117 | 0 | 0 | 0 |
| 2880 | LN908364 | Candidatus Nitrosopumilus sp. HCA1 (KF957663.1) | 98.0 | 72 | 2 | 16 | 1 | 0 | 0 |
| 2893 | LN908365 | Candidatus Nitrosopelagicus brevis strain CN25 (CP007026.1) | 96.0 | 717 | 18 | 95 | 0 | 0 | 0 |
| 2928 | LN908366 | Candidatus Nitrosopumilus sp. NF5 (CP011070.1) and Candidatus Nitrosopumilus sp. D3C (CP010868.1) | 96.0 | 120 | 1 | 13 | 0 | 0 | 0 |
| 2960 | LN908367 | Candidatus Nitrosopelagicus brevis strain CN25 (CP007026.1) | 96.0 | 6 | 0 | 0 | 0 | 0 | 0 |
| 2967 | LN908368 | Candidatus Nitrosopumilus sp. NF5 (CP011070.1) and Candidatus Nitrosopumilus sp. D3C (CP010868.1) | 93.0 | 1 | 0 | 0 | 0 | 0 | 0 |
| 2976 | LN908369 | Candidatus Nitrosopelagicus brevis strain CN25 (CP007026.1) | 96.0 | 1 | 1 | 1 | 0 | 0 | 0 |
| 2984 | LN908370 | Candidatus Nitrosopelagicus brevis strain CN25 (CP007026.1) | 96.0 | 157 | 11 | 39 | 0 | 0 | 0 |
| 2990 | LN908371 | Candidatus Nitrosopumilus sp. HCA1 (KF957663.1) | 93.0 | 3 | 0 | 0 | 0 | 0 | 0 |
| 2996 | LN908372 | Candidatus Nitrosopelagicus brevis strain CN25 (CP007026.1) | 95.0 | 940 | 68 | 200 | 0 | 0 | 0 |
| 3000 | LN908373 | Candidatus Nitrosopelagicus brevis strain CN25 (CP007026.1) | 95.0 | 54 | 1 | 2 | 0 | 0 | 0 |
| 3010 | LN908374 | Candidatus Nitrosopumilus sp. NF5 (CP011070.1) and Candidatus Nitrosopumilus sp. D3C (CP010868.1) | 93.0 | 1 | 0 | 0 | 0 | 0 | 0 |
| 3049 | LN908375 | Candidatus Nitrosopelagicus brevis strain CN25 (CP007026.1) | 96.0 | 21 | 1 | 7 | 1 | 0 | 0 |
| 3112 | LN908376 | Candidatus Nitrosopelagicus brevis strain CN25 (CP007026.1) | 92.0 | 1 | 0 | 0 | 0 | 0 | 0 |
| 3130 | LN908377 | Candidatus Nitrosopumilus sp. NF5 (CP011070.1) and Candidatus Nitrosopumilus sp. D3C (CP010868.1) | 97.0 | 6 | 0 | 0 | 0 | 0 | 0 |
| 3153 | LN908378 | Candidatus Nitrosopelagicus brevis strain CN25 (CP007026.1) | 97.0 | 305 | 25 | 58 | 0 | 0 | 0 |
| 3159 | LN908379 | Candidatus Nitrosopelagicus brevis strain CN25 (CP007026.1) | 95.0 | 3882 | 326 | 1985 | 2 | 1 | 0 |
| 3167 | LN908380 | Candidatus Nitrosopelagicus brevis strain CN25 (CP007026.1) | 96.0 | 306 | 34 | 130 | 0 | 0 | 0 |
| 3207 | LN908381 | Candidatus Nitrosopelagicus brevis strain CN25 (CP007026.1) | 96.0 | 694 | 48 | 113 | 0 | 0 | 0 |
| 3257 | LN908382 | Candidatus Nitrosopumilus sp. NF5 (CP011070.1) and Candidatus Nitrosopumilus sp. D3C (CP010868.1) | 97.0 | 975 | 68 | 162 | 0 | 0 | 0 |
| 3258 | LN908383 | Candidatus Nitrosopumilus sp. NF5 (CP011070.1) and Candidatus Nitrosopumilus sp. D3C (CP010868.1) | 95.0 | 159 | 17 | 47 | 0 | 0 | 0 |
| 3280 | LN908384 | Candidatus Nitrosopelagicus brevis strain CN25 (CP007026.1) | 98.0 | 267 | 4 | 33 | 0 | 0 | 0 |
| 3281 | LN908385 | Candidatus Nitrosopelagicus brevis strain CN25 (CP007026.1) | 97.0 | 665 | 61 | 184 | 0 | 0 | 0 |
| 3286 | LN908386 | Candidatus Nitrosopelagicus brevis strain CN25 (CP007026.1) | 95.0 | 74 | 5 | 11 | 0 | 0 | 0 |
| 3295 | LN908387 | Candidatus Nitrosopumilus sp. HCA1 (KF957663.1) | 93.0 | 193 | 4 | 6 | 0 | 0 | 1 |
| 3306 | LN908388 | Candidatus Nitrosopelagicus brevis strain CN25 (CP007026.1) | 95.0 | 269 | 11 | 69 | 0 | 0 | 0 |
| 3307 | LN908389 | Candidatus Nitrosopelagicus brevis strain CN25 (CP007026.1) | 96.0 | 374 | 29 | 60 | 0 | 0 | 0 |
| 3315 | LN908390 | Candidatus Nitrosopumilus sp. HCA1 (KF957663.1) | 95.0 | 1 | 1 | 2 | 0 | 0 | 0 |
| 3351 | LN908391 | Candidatus Nitrosopumilus sp. NF5 (CP011070.1) and Candidatus Nitrosopumilus sp. D3C (CP010868.1) | 89.0 | 1 | 0 | 1 | 0 | 0 | 0 |
| 3363 | LN908392 | Candidatus Nitrosopumilus sp. NF5 (CP011070.1) and Candidatus Nitrosopumilus sp. D3C (CP010868.1) | 92.0 | 1 | 0 | 0 | 0 | 0 | 0 |
| 3375 | LN908393 | Candidatus Nitrosopumilus sp. NF5 (CP011070.1) and Candidatus Nitrosopumilus sp. D3C (CP010868.1) | 93.0 | 1 | 2 | 1 | 0 | 0 | 0 |
| 3377 | LN908394 | Candidatus Nitrosopumilus sp. NF5 (CP011070.1) and Candidatus Nitrosopumilus sp. D3C (CP010868.1) | 96.0 | 44 | 7 | 16 | 0 | 0 | 0 |
| 3398 | LN908395 | Candidatus Nitrosopumilus sp. NF5 (CP011070.1) and Candidatus Nitrosopumilus sp. D3C (CP010868.1) | 92.0 | 2 | 0 | 0 | 0 | 0 | 0 |
| 3411 | LN908396 | Candidatus Nitrosopelagicus brevis strain CN25 (CP007026.1) | 96.0 | 497 | 7 | 70 | 0 | 0 | 0 |
| 3434 | LN908397 | Candidatus Nitrosopelagicus brevis strain CN25 (CP007026.1) | 98.0 | 1864 | 40 | 128 | 0 | 0 | 0 |
| 3437 | LN908398 | Candidatus Nitrosopumilus sp. NF5 (CP011070.1) and Candidatus Nitrosopumilus sp. D3C (CP010868.1) | 96.0 | 845 | 71 | 227 | 0 | 0 | 0 |
| 3438 | LN908399 | Candidatus Nitrosopumilus sp. NF5 (CP011070.1) and Candidatus Nitrosopumilus sp. D3C (CP010868.1) | 89.0 | 1 | 0 | 0 | 0 | 0 | 0 |
| 3441 | LN908400 | Candidatus Nitrosopelagicus brevis strain CN25 (CP007026.1) | 96.0 | 2525 | 249 | 491 | 0 | 0 | 0 |
| 3446 | LN908401 | Candidatus Nitrosopumilus sp. NF5 (CP011070.1) and Candidatus Nitrosopumilus sp. D3C (CP010868.1) | 91.0 | 1 | 0 | 3 | 0 | 0 | 0 |
| 3472 | LN908402 | Candidatus Nitrosopelagicus brevis strain CN25 (CP007026.1) | 94.0 | 448 | 44 | 96 | 0 | 0 | 0 |
| 3479 | LN908403 | Candidatus Nitrosopumilus sp. NF5 (CP011070.1) and Candidatus Nitrosopumilus sp. D3C (CP010868.1) | 94.0 | 1 | 0 | 0 | 0 | 0 | 0 |
| 3491 | LN908404 | Candidatus Nitrosopelagicus brevis strain CN25 (CP007026.1) | 93.0 | 1 | 0 | 0 | 0 | 0 | 0 |
| 3492 | LN908405 | Candidatus Nitrosopelagicus brevis strain CN25 (CP007026.1) | 95.0 | 2 | 1 | 1 | 0 | 0 | 0 |
| 3509 | LN908406 | *Nitrosopumilus maritimus* strain SCM1 (NR_102913) | 98.0 | 884 | 27 | 85 | 0 | 0 | 0 |
| 3547 | LN908407 | Candidatus Nitrosopumilus sp. NF5 (CP011070.1) and Candidatus Nitrosopumilus sp. D3C (CP010868.1) | 89.0 | 1 | 0 | 2 | 0 | 0 | 0 |
| 3555 | LN908408 | Candidatus Nitrosopelagicus brevis strain CN25 (CP007026.1) | 97.0 | 1 | 0 | 1 | 0 | 0 | 0 |
| 3557 | LN908409 | Candidatus Nitrosopumilus sp. NF5 (CP011070.1) and Candidatus Nitrosopumilus sp. D3C (CP010868.1) | 88.0 | 1 | 0 | 0 | 0 | 0 | 0 |
| 3572 | LN908410 | Candidatus Nitrosopelagicus brevis strain CN25 (CP007026.1) | 95.0 | 407 | 42 | 104 | 0 | 0 | 0 |
| 3573 | LN908411 | Candidatus Nitrosopumilus sp. NF5 (CP011070.1) and Candidatus Nitrosopumilus sp. D3C (CP010868.1) | 96.0 | 590 | 45 | 164 | 0 | 0 | 1 |
| 3576 | LN908412 | Candidatus Nitrosopelagicus brevis strain CN25 (CP007026.1) | 96.0 | 19 | 3 | 4 | 0 | 0 | 0 |
| 3594 | LN908413 | Candidatus Nitrosopumilus sp. PS0 (KF957664.1) | 97.0 | 32 | 2 | 22 | 0 | 0 | 0 |
| 3620 | LN908414 | Candidatus Nitrosopelagicus brevis strain CN25 (CP007026.1) | 87.0 | 2 | 0 | 0 | 0 | 0 | 0 |
| 3646 | LN908415 | Candidatus Nitrosopumilus sp. PS0 (KF957664.1) | 97.0 | 2 | 2 | 0 | 0 | 0 | 0 |
| 3724 | LN908416 | Candidatus Nitrosopelagicus brevis strain CN25 (CP007026.1) | 95.0 | 116 | 3 | 22 | 0 | 0 | 0 |
| 3746 | LN908417 | Candidatus Nitrosopumilus sp. NF5 (CP011070.1) and Candidatus Nitrosopumilus sp. D3C (CP010868.1) | 91.0 | 6 | 0 | 1 | 0 | 0 | 0 |
| 3748 | LN908418 | Candidatus Nitrosopumilus sp. NF5 (CP011070.1) and Candidatus Nitrosopumilus sp. D3C (CP010868.1) | 91.0 | 481 | 11 | 42 | 0 | 0 | 0 |
| 3753 | LN908419 | Candidatus Nitrosopelagicus brevis strain CN25 (CP007026.1) | 100.0 | 460 | 40 | 101 | 0 | 1 | 0 |
| 3778 | LN908420 | Candidatus Nitrosopumilus sp. NF5 (CP011070.1) and Candidatus Nitrosopumilus sp. D3C (CP010868.1) | 97.0 | 1360 | 142 | 414 | 0 | 0 | 0 |
| 3783 | LN908421 | Candidatus Nitrosopumilus sp. NF5 (CP011070.1) and Candidatus Nitrosopumilus sp. D3C (CP010868.1) | 97.0 | 1 | 0 | 0 | 0 | 0 | 0 |
| 3822 | LN908422 | Candidatus Nitrosopumilus sp. HCA1 (KF957663.1) | 95.0 | 526 | 11 | 105 | 0 | 0 | 0 |
| 3841 | LN908423 | Candidatus Nitrosopumilus sp. NF5 (CP011070.1) and Candidatus Nitrosopumilus sp. D3C (CP010868.1) | 90.0 | 2 | 0 | 0 | 0 | 0 | 0 |
| 3856 | LN908424 | Candidatus Nitrosopumilus sp. NF5 (CP011070.1) and Candidatus Nitrosopumilus sp. D3C (CP010868.1) | 96.0 | 2 | 1 | 1 | 0 | 0 | 0 |
| 3875 | LN908425 | Candidatus Nitrosopumilus sp. NF5 (CP011070.1) and Candidatus Nitrosopumilus sp. D3C (CP010868.1) | 97.0 | 77 | 5 | 28 | 0 | 0 | 0 |
| 3888 | LN908426 | Candidatus Nitrosopelagicus brevis strain CN25 (CP007026.1) | 95.0 | 39 | 1 | 12 | 0 | 0 | 0 |
| 3905 | LN908427 | Candidatus Nitrosopelagicus brevis strain CN25 (CP007026.1) | 95.0 | 27 | 5 | 16 | 0 | 0 | 0 |
| 3911 | LN908428 | Candidatus Nitrosopelagicus brevis strain CN25 (CP007026.1) | 95.0 | 685 | 84 | 531 | 0 | 0 | 0 |
| 3913 | LN908429 | Candidatus Nitrosopelagicus brevis strain CN25 (CP007026.1) | 94.0 | 1 | 0 | 0 | 0 | 0 | 0 |
| 3926 | LN908430 | Candidatus Nitrosopelagicus brevis strain CN25 (CP007026.1) | 96.0 | 364 | 33 | 168 | 0 | 0 | 0 |
| 3941 | LN908431 | Candidatus Nitrosopelagicus brevis strain CN25 (CP007026.1) | 97.0 | 74 | 4 | 14 | 0 | 0 | 0 |
| 4000 | LN908432 | Candidatus Nitrosopumilus sp. NF5 (CP011070.1) and Candidatus Nitrosopumilus sp. D3C (CP010868.1) | 97.0 | 147 | 20 | 72 | 0 | 0 | 0 |
| 4003 | LN908433 | Candidatus Nitrosopelagicus brevis strain CN25 (CP007026.1) | 96.0 | 608 | 21 | 71 | 0 | 0 | 0 |
| 4054 | LN908434 | Candidatus Nitrosopumilus sp. NF5 (CP011070.1) and Candidatus Nitrosopumilus sp. D3C (CP010868.1) | 96.0 | 829 | 89 | 209 | 0 | 0 | 0 |
| 4064 | LN908435 | Candidatus Nitrosopumilus sp. HCA1 (KF957663.1) | 100.0 | 1251 | 208 | 405 | 0 | 0 | 0 |
| 4072 | LN908436 | Candidatus Nitrosopumilus sp. NF5 (CP011070.1) and Candidatus Nitrosopumilus sp. D3C (CP010868.1) | 96.0 | 449 | 40 | 165 | 0 | 0 | 0 |
| 4078 | LN908437 | Candidatus Nitrosopelagicus brevis strain CN25 (CP007026.1) | 96.0 | 188 | 16 | 86 | 0 | 0 | 0 |
| 4081 | LN908438 | Candidatus Nitrosopelagicus brevis strain CN25 (CP007026.1) | 95.0 | 1462 | 173 | 426 | 0 | 0 | 0 |
| 4104 | LN908439 | Candidatus Nitrosopelagicus brevis strain CN25 (CP007026.1) | 95.0 | 278 | 21 | 73 | 0 | 0 | 0 |
| 4114 | LN908440 | Candidatus Nitrosopumilus sp. NF5 (CP011070.1) and Candidatus Nitrosopumilus sp. D3C (CP010868.1) | 94.0 | 191 | 24 | 80 | 0 | 0 | 0 |
| 4143 | LN908441 | Candidatus Nitrosopelagicus brevis strain CN25 (CP007026.1) | 95.0 | 3 | 0 | 0 | 0 | 0 | 0 |
| 4168 | LN908442 | Candidatus Nitrosopumilus sp. NF5 (CP011070.1) and Candidatus Nitrosopumilus sp. D3C (CP010868.1) | 88.0 | 1 | 0 | 0 | 0 | 0 | 0 |
| 4176 | LN908443 | Candidatus Nitrosopumilus sp. NF5 (CP011070.1) and Candidatus Nitrosopumilus sp. D3C (CP010868.1) | 88.0 | 1 | 0 | 0 | 0 | 0 | 0 |
| 4206 | LN908444 | Candidatus Nitrosopelagicus brevis strain CN25 (CP007026.1) | 96.0 | 2265 | 201 | 588 | 0 | 0 | 0 |
| 4212 | LN908445 | Candidatus Nitrosopumilus sp. NF5 (CP011070.1) and Candidatus Nitrosopumilus sp. D3C (CP010868.1) | 95.0 | 94 | 14 | 34 | 0 | 0 | 0 |
| 4213 | LN908446 | Candidatus Nitrosopumilus sp. NF5 (CP011070.1) and Candidatus Nitrosopumilus sp. D3C (CP010868.1) | 89.0 | 1 | 0 | 0 | 0 | 0 | 0 |
| 4216 | LN908447 | Candidatus Nitrosopumilus sp. NF5 (CP011070.1) and Candidatus Nitrosopumilus sp. D3C (CP010868.1) | 97.0 | 362 | 51 | 131 | 0 | 0 | 0 |
| 4228 | LN908448 | Candidatus Nitrosopumilus sp. NF5 (CP011070.1) and Candidatus Nitrosopumilus sp. D3C (CP010868.1) | 97.0 | 1437 | 211 | 447 | 0 | 0 | 0 |
| 4259 | LN908449 | Candidatus Nitrosopelagicus brevis strain CN25 (CP007026.1) | 98.0 | 1 | 0 | 0 | 0 | 0 | 0 |
| 4264 | LN908450 | Candidatus Nitrosopelagicus brevis strain CN25 (CP007026.1) | 96.0 | 359 | 45 | 135 | 0 | 0 | 1 |
| 4274 | LN908451 | Candidatus Nitrosopumilus sp. NF5 (CP011070.1) and Candidatus Nitrosopumilus sp. D3C (CP010868.1) | 96.0 | 464 | 61 | 148 | 0 | 0 | 0 |
| 4286 | LN908452 | Candidatus Nitrosopumilus sp. NF5 (CP011070.1) and Candidatus Nitrosopumilus sp. D3C (CP010868.1) | 96.0 | 9 | 1 | 3 | 0 | 0 | 0 |
| 4339 | LN908453 | Candidatus Nitrosopumilus sp. NF5 (CP011070.1) and Candidatus Nitrosopumilus sp. D3C (CP010868.1) | 96.0 | 27 | 5 | 7 | 0 | 0 | 0 |
| 4346 | LN908454 | Candidatus Nitrosopumilus sp. NF5 (CP011070.1) and Candidatus Nitrosopumilus sp. D3C (CP010868.1) | 96.0 | 182 | 23 | 84 | 0 | 0 | 2 |
| 4375 | LN908455 | Candidatus Nitrosopelagicus brevis strain CN25 (CP007026.1) | 98.0 | 2149 | 289 | 669 | 0 | 0 | 0 |
| 4401 | LN908456 | Candidatus Nitrosopelagicus brevis strain CN25 (CP007026.1) | 96.0 | 53 | 13 | 13 | 0 | 0 | 1 |
| 4403 | LN908457 | Candidatus Nitrosopumilus sp. NF5 (CP011070.1) and Candidatus Nitrosopumilus sp. D3C (CP010868.1) | 96.0 | 59 | 12 | 56 | 0 | 0 | 2 |
| 4415 | LN908458 | Candidatus Nitrosopelagicus brevis strain CN25 (CP007026.1) | 88.0 | 1 | 0 | 0 | 0 | 0 | 0 |
| 4418 | LN908459 | Candidatus Nitrosopumilus sp. NF5 (CP011070.1) and Candidatus Nitrosopumilus sp. D3C (CP010868.1) | 98.0 | 5 | 0 | 5 | 0 | 0 | 0 |
| 4436 | LN908460 | Candidatus Nitrosopumilus sp. HCA1 (KF957663.1) | 98.0 | 137 | 18 | 43 | 0 | 0 | 0 |
| 4438 | LN908461 | Candidatus Nitrosopelagicus brevis strain CN25 (CP007026.1) | 98.0 | 1 | 0 | 0 | 0 | 0 | 0 |
| 4446 | LN908462 | Candidatus Nitrosopelagicus brevis strain CN25 (CP007026.1) | 94.0 | 2 | 0 | 0 | 0 | 0 | 0 |
| 4453 | LN908463 | Candidatus Nitrosopelagicus brevis strain CN25 (CP007026.1) | 96.0 | 11 | 1 | 2 | 0 | 0 | 0 |
| 4464 | LN908464 | Candidatus Nitrosopelagicus brevis strain CN25 (CP007026.1) | 94.0 | 1 | 1 | 0 | 0 | 0 | 0 |
| 4478 | LN908465 | Candidatus Nitrosopelagicus brevis strain CN25 (CP007026.1) | 93.0 | 3 | 2 | 4 | 0 | 0 | 0 |
| 4487 | LN908466 | Candidatus Nitrosopelagicus brevis strain CN25 (CP007026.1) | 96.0 | 4139 | 608 | 1596 | 0 | 0 | 0 |
| 4504 | LN908467 | Candidatus Nitrosopelagicus brevis strain CN25 (CP007026.1) | 95.0 | 5 | 2 | 5 | 0 | 0 | 0 |
| 4516 | LN908468 | Candidatus Nitrosopumilus sp. NF5 (CP011070.1) and Candidatus Nitrosopumilus sp. D3C (CP010868.1) | 90.0 | 1 | 0 | 0 | 0 | 0 | 0 |
| 4519 | LN908469 | Candidatus Nitrosopelagicus brevis strain CN25 (CP007026.1) | 96.0 | 1 | 0 | 0 | 0 | 0 | 0 |
| 4544 | LN908470 | Candidatus Nitrosopumilus sp. HCA1 (KF957663.1) | 98.0 | 1403 | 244 | 715 | 0 | 3 | 3 |
| 4553 | LN908471 | Candidatus Nitrosopelagicus brevis strain CN25 (CP007026.1) | 93.0 | 1 | 0 | 0 | 0 | 0 | 0 |
| 4564 | LN908472 | Candidatus Nitrosopelagicus brevis strain CN25 (CP007026.1) | 96.0 | 3 | 0 | 14 | 0 | 0 | 0 |
| 4566 | LN908473 | Candidatus Nitrosopelagicus brevis strain CN25 (CP007026.1) | 94.0 | 1 | 0 | 2 | 0 | 0 | 0 |
| 4600 | LN908474 | Candidatus Nitrosopumilus sp. NF5 (CP011070.1) and Candidatus Nitrosopumilus sp. D3C (CP010868.1) | 95.0 | 1 | 0 | 0 | 0 | 0 | 0 |
| 4603 | LN908475 | Candidatus Nitrosopelagicus brevis strain CN25 (CP007026.1) | 95.0 | 2 | 0 | 459 | 0 | 0 | 0 |
| 4623 | LN908476 | Candidatus Nitrosopumilus sp. NF5 (CP011070.1) and Candidatus Nitrosopumilus sp. D3C (CP010868.1) | 89.0 | 2 | 0 | 0 | 0 | 0 | 0 |
| 4657 | LN908477 | Candidatus Nitrosopumilus sp. NF5 (CP011070.1) and Candidatus Nitrosopumilus sp. D3C (CP010868.1) | 89.0 | 2 | 0 | 0 | 0 | 0 | 0 |
| 4700 | LN908478 | Candidatus Nitrosopelagicus brevis strain CN25 (CP007026.1) | 98.0 | 4 | 0 | 81 | 1 | 0 | 0 |
| 4725 | LN908479 | Candidatus Nitrosopumilus sp. NF5 (CP011070.1) and Candidatus Nitrosopumilus sp. D3C (CP010868.1) | 96.0 | 2 | 0 | 24 | 0 | 0 | 0 |
| 4745 | LN908480 | Candidatus Nitrosopumilus sp. PS0 (KF957664.1) | 94.0 | 1 | 0 | 3 | 0 | 0 | 0 |
| 4772 | LN908481 | Candidatus Nitrosopumilus sp. NF5 (CP011070.1) and Candidatus Nitrosopumilus sp. D3C (CP010868.1) | 98.0 | 1 | 0 | 42 | 0 | 0 | 0 |
| 4776 | LN908482 | Candidatus Nitrosopumilus sp. NF5 (CP011070.1) and Candidatus Nitrosopumilus sp. D3C (CP010868.1) | 88.0 | 1 | 0 | 4 | 0 | 0 | 0 |
| 4793 | LN908483 | Candidatus Nitrosopelagicus brevis strain CN25 (CP007026.1) | 96.0 | 1 | 0 | 40 | 0 | 0 | 0 |
| 4823 | LN908484 | Candidatus Nitrosopelagicus brevis strain CN25 (CP007026.1) | 89.0 | 1 | 0 | 0 | 0 | 0 | 0 |
| 4829 | LN908485 | Candidatus Nitrosopelagicus brevis strain CN25 (CP007026.1) | 96.0 | 3 | 0 | 35 | 0 | 0 | 0 |
| 4841 | LN908486 | Candidatus Nitrosopelagicus brevis strain CN25 (CP007026.1) | 94.0 | 1 | 0 | 0 | 0 | 0 | 0 |
| 4844 | LN908487 | Candidatus Nitrosopelagicus brevis strain CN25 (CP007026.1) | 92.0 | 2 | 0 | 1 | 0 | 0 | 0 |
| 4891 | LN908488 | Candidatus Nitrosopelagicus brevis strain CN25 (CP007026.1) | 93.0 | 1 | 3 | 3 | 0 | 0 | 0 |
| 4909 | LN908489 | Candidatus Nitrosopumilus sp. NF5 (CP011070.1) and Candidatus Nitrosopumilus sp. D3C (CP010868.1) | 96.0 | 1 | 0 | 11 | 0 | 0 | 0 |
| 4953 | LN908490 | Candidatus Nitrosopelagicus brevis strain CN25 (CP007026.1) | 96.0 | 1 | 0 | 21 | 0 | 0 | 0 |
| 4956 | LN908491 | Candidatus Nitrosopumilus sp. NF5 (CP011070.1) and Candidatus Nitrosopumilus sp. D3C (CP010868.1) | 96.0 | 1 | 0 | 35 | 0 | 0 | 0 |
| 4959 | LN908492 | Candidatus Nitrosopumilus sp. NF5 (CP011070.1) and Candidatus Nitrosopumilus sp. D3C (CP010868.1) | 96.0 | 8 | 0 | 409 | 0 | 0 | 0 |
| 4967 | LN908493 | Candidatus Nitrosopumilus sp. NF5 (CP011070.1) and Candidatus Nitrosopumilus sp. D3C (CP010868.1) | 96.0 | 1 | 0 | 8 | 0 | 0 | 0 |
| 4972 | LN908494 | Candidatus Nitrosopumilus sp. NF5 (CP011070.1) and Candidatus Nitrosopumilus sp. D3C (CP010868.1) | 96.0 | 3 | 0 | 78 | 0 | 0 | 0 |
| 4973 | LN908495 | Candidatus Nitrosopelagicus brevis strain CN25 (CP007026.1) | 87.0 | 1 | 0 | 0 | 0 | 0 | 0 |
| 5012 | LN908496 | Candidatus Nitrosopelagicus brevis strain CN25 (CP007026.1) | 96.0 | 2 | 0 | 19 | 0 | 0 | 0 |
| 5055 | LN908497 | Candidatus Nitrosopumilus sp. NF5 (CP011070.1) and Candidatus Nitrosopumilus sp. D3C (CP010868.1) | 96.0 | 1 | 0 | 3 | 0 | 0 | 0 |
| 5085 | LN908498 | Candidatus Nitrosopumilus sp. NF5 (CP011070.1) and Candidatus Nitrosopumilus sp. D3C (CP010868.1) | 97.0 | 1 | 0 | 20 | 0 | 0 | 0 |
| 5086 | LN908499 | Candidatus Nitrosopumilus sp. NF5 (CP011070.1) and Candidatus Nitrosopumilus sp. D3C (CP010868.1) | 96.0 | 1 | 0 | 58 | 0 | 0 | 0 |
| 5151 | LN908500 | Candidatus Nitrosopumilus sp. NF5 (CP011070.1) and Candidatus Nitrosopumilus sp. D3C (CP010868.1) | 90.0 | 1 | 0 | 0 | 0 | 0 | 0 |
| 5165 | LN908501 | *Nitrosopumilus maritimus* strain SCM1 (NR_102913) | 94.0 | 1 | 0 | 3 | 0 | 0 | 0 |
| 5169 | LN908502 | Candidatus Nitrosopumilus sp. NF5 (CP011070.1) and Candidatus Nitrosopumilus sp. D3C (CP010868.1) | 95.0 | 1 | 0 | 21 | 0 | 0 | 0 |
| 5177 | LN908503 | Candidatus Nitrosopumilus sp. NF5 (CP011070.1) and Candidatus Nitrosopumilus sp. D3C (CP010868.1) | 89.0 | 1 | 0 | 0 | 0 | 0 | 0 |
| 5216 | LN908504 | Candidatus Nitrosopelagicus brevis strain CN25 (CP007026.1) | 88.0 | 1 | 0 | 0 | 0 | 0 | 0 |
| 5224 | LN908505 | Candidatus Nitrosopelagicus brevis strain CN25 (CP007026.1) | 96.0 | 1 | 0 | 24 | 0 | 0 | 0 |
| 5235 | LN908506 | Candidatus Nitrosopelagicus brevis strain CN25 (CP007026.1) | 96.0 | 2 | 0 | 85 | 0 | 0 | 1 |
| 5240 | LN908507 | Candidatus Nitrosopumilus sp. NF5 (CP011070.1) and Candidatus Nitrosopumilus sp. D3C (CP010868.1) | 96.0 | 2 | 0 | 0 | 0 | 0 | 0 |
| 5254 | LN908508 | Candidatus Nitrosopumilus sp. HCA1 (KF957663.1) | 91.0 | 1 | 0 | 0 | 0 | 0 | 0 |
| 5286 | LN908509 | Candidatus Nitrosopelagicus brevis strain CN25 (CP007026.1) | 94.0 | 1 | 0 | 1 | 0 | 0 | 0 |
| 5307 | LN908510 | Candidatus Nitrosopelagicus brevis strain CN25 (CP007026.1) | 89.0 | 2 | 0 | 0 | 0 | 0 | 0 |
| 5312 | LN908511 | Candidatus Nitrosopumilus sp. HCA1 (KF957663.1) | 98.0 | 4 | 0 | 26 | 81 | 2 | 7 |
| 5318 | LN908512 | Candidatus Nitrosopelagicus brevis strain CN25 (CP007026.1) | 89.0 | 1 | 0 | 0 | 0 | 0 | 0 |
| 5319 | LN908513 | Candidatus Nitrosopelagicus brevis strain CN25 (CP007026.1) | 91.0 | 1 | 0 | 1 | 0 | 0 | 0 |
| 5382 | LN908514 | Candidatus Nitrosopumilus sp. NF5 (CP011070.1) and Candidatus Nitrosopumilus sp. D3C (CP010868.1) | 92.0 | 1 | 0 | 0 | 0 | 0 | 0 |
| 5389 | LN908515 | Candidatus Nitrosopelagicus brevis strain CN25 (CP007026.1) | 96.0 | 1 | 0 | 1 | 0 | 0 | 0 |
| 5390 | LN908516 | Candidatus Nitrosopumilus sp. NF5 (CP011070.1) and Candidatus Nitrosopumilus sp. D3C (CP010868.1) | 95.0 | 1 | 2 | 0 | 0 | 0 | 0 |
| 5394 | LN908517 | Candidatus Nitrosopelagicus brevis strain CN25 (CP007026.1) | 95.0 | 1 | 0 | 62 | 0 | 0 | 0 |
| 5397 | LN908518 | Candidatus Nitrosopumilus sp. NF5 (CP011070.1) and Candidatus Nitrosopumilus sp. D3C (CP010868.1) | 96.0 | 1 | 0 | 1 | 0 | 0 | 0 |
| 5418 | LN908519 | Candidatus Nitrosopelagicus brevis strain CN25 (CP007026.1) | 95.0 | 3 | 0 | 5 | 0 | 0 | 0 |
| 5424 | LN908520 | *Nitrosopumilus maritimus* strain SCM1 (NR_102913) | 94.0 | 2 | 2 | 36 | 0 | 0 | 0 |
| 5435 | LN908521 | Candidatus Nitrosopumilus sp. NF5 (CP011070.1) and Candidatus Nitrosopumilus sp. D3C (CP010868.1) | 96.0 | 4 | 2 | 107 | 0 | 0 | 2 |
| 5440 | LN908522 | Candidatus Nitrosopelagicus brevis strain CN25 (CP007026.1) | 92.0 | 1 | 0 | 0 | 0 | 0 | 0 |
| 5456 | LN908523 | Candidatus Nitrosopelagicus brevis strain CN25 (CP007026.1) | 97.0 | 1 | 0 | 0 | 0 | 0 | 0 |
| 5511 | LN908524 | Candidatus Nitrosopumilus sp. NF5 (CP011070.1) and Candidatus Nitrosopumilus sp. D3C (CP010868.1) | 91.0 | 2 | 0 | 0 | 0 | 0 | 0 |
| 5512 | LN908525 | Candidatus Nitrosopelagicus brevis strain CN25 (CP007026.1) | 94.0 | 1 | 0 | 0 | 0 | 0 | 0 |
| 5543 | LN908526 | Candidatus Nitrosopumilus sp. NF5 (CP011070.1) and Candidatus Nitrosopumilus sp. D3C (CP010868.1) | 96.0 | 5 | 0 | 591 | 0 | 0 | 0 |
| 5544 | LN908527 | Candidatus Nitrosopelagicus brevis strain CN25 (CP007026.1) | 96.0 | 1 | 2 | 0 | 0 | 0 | 0 |
| 5550 | LN908528 | Candidatus Nitrosopelagicus brevis strain CN25 (CP007026.1) | 93.0 | 1 | 0 | 0 | 0 | 0 | 0 |
| 5556 | LN908529 | Candidatus Nitrosopelagicus brevis strain CN25 (CP007026.1) | 94.0 | 1 | 0 | 11 | 0 | 0 | 0 |
| 5586 | LN908530 | Candidatus Nitrosopelagicus brevis strain CN25 (CP007026.1) | 95.0 | 10 | 1 | 142 | 0 | 2 | 0 |
| 5594 | LN908531 | Candidatus Nitrosopumilus sp. NF5 (CP011070.1) and Candidatus Nitrosopumilus sp. D3C (CP010868.1) | 97.0 | 1 | 0 | 0 | 0 | 0 | 0 |
| 5660 | LN908532 | Candidatus Nitrosopumilus sp. NF5 (CP011070.1) and Candidatus Nitrosopumilus sp. D3C (CP010868.1) | 87.0 | 1 | 2 | 1 | 0 | 0 | 0 |
| 5689 | LN908533 | Candidatus Nitrosopumilus sp. HCA1 (KF957663.1) | 92.0 | 1 | 0 | 0 | 0 | 0 | 0 |
| 5691 | LN908534 | Candidatus Nitrosopumilus sp. NF5 (CP011070.1) and Candidatus Nitrosopumilus sp. D3C (CP010868.1) | 93.0 | 1 | 0 | 0 | 0 | 0 | 0 |
| 5742 | LN908535 | Candidatus Nitrosopelagicus brevis strain CN25 (CP007026.1) | 95.0 | 2 | 0 | 91 | 0 | 0 | 1 |
| 5746 | LN908536 | Candidatus Nitrosopumilus sp. NF5 (CP011070.1) and Candidatus Nitrosopumilus sp. D3C (CP010868.1) | 97.0 | 1 | 0 | 0 | 0 | 0 | 0 |
| 5823 | LN908537 | Candidatus Nitrosopelagicus brevis strain CN25 (CP007026.1) | 94.0 | 1 | 0 | 2 | 0 | 0 | 0 |
| 5836 | LN908538 | Candidatus Nitrosopelagicus brevis strain CN25 (CP007026.1) | 96.0 | 2 | 0 | 166 | 0 | 1 | 0 |
| 5881 | LN908539 | Candidatus Nitrosopumilus sp. NF5 (CP011070.1) and Candidatus Nitrosopumilus sp. D3C (CP010868.1) | 94.0 | 1 | 0 | 8 | 1 | 0 | 0 |
| 5888 | LN908540 | Candidatus Nitrosopelagicus brevis strain CN25 (CP007026.1) | 96.0 | 3 | 0 | 113 | 0 | 0 | 0 |
| 5918 | LN908541 | Candidatus Nitrosopelagicus brevis strain CN25 (CP007026.1) | 93.0 | 1 | 0 | 0 | 0 | 0 | 0 |
| 5923 | LN908542 | Candidatus Nitrosopelagicus brevis strain CN25 (CP007026.1) | 96.0 | 2 | 0 | 251 | 0 | 0 | 0 |
| 5929 | LN908543 | Candidatus Nitrosopumilus sp. NF5 (CP011070.1) and Candidatus Nitrosopumilus sp. D3C (CP010868.1) | 97.0 | 1 | 0 | 7 | 0 | 0 | 1 |
| 5945 | LN908544 | Candidatus Nitrosopumilus sp. NF5 (CP011070.1) and Candidatus Nitrosopumilus sp. D3C (CP010868.1) | 95.0 | 1 | 0 | 88 | 0 | 0 | 0 |
| 5953 | LN908545 | Candidatus Nitrosopelagicus brevis strain CN25 (CP007026.1) | 93.0 | 0 | 2 | 2 | 0 | 0 | 0 |
| 5962 | LN908546 | Candidatus Nitrosopelagicus brevis strain CN25 (CP007026.1) | 94.0 | 0 | 1 | 0 | 0 | 0 | 0 |
| 5977 | LN908547 | Candidatus Nitrosopelagicus brevis strain CN25 (CP007026.1) | 93.0 | 0 | 2 | 5 | 0 | 0 | 0 |
| 5983 | LN908548 | Candidatus Nitrosopelagicus brevis strain CN25 (CP007026.1) | 94.0 | 0 | 1 | 8 | 0 | 0 | 0 |
| 6005 | LN908549 | Candidatus Nitrosopumilus sp. NF5 (CP011070.1) and Candidatus Nitrosopumilus sp. D3C (CP010868.1) | 95.0 | 0 | 1 | 6 | 0 | 0 | 0 |
| 6023 | LN908550 | Candidatus Nitrosopumilus sp. NF5 (CP011070.1) and Candidatus Nitrosopumilus sp. D3C (CP010868.1) | 89.0 | 0 | 1 | 0 | 0 | 0 | 0 |
| 6027 | LN908551 | Candidatus Nitrosopumilus sp. NF5 (CP011070.1) and Candidatus Nitrosopumilus sp. D3C (CP010868.1) | 89.0 | 0 | 1 | 0 | 0 | 0 | 0 |
| 6051 | LN908552 | Candidatus Nitrosopumilus sp. HCA1 (KF957663.1) | 98.0 | 1 | 3 | 16 | 0 | 0 | 0 |
| 6057 | LN908553 | Candidatus Nitrosopumilus sp. HCA1 (KF957663.1) | 98.0 | 1 | 1 | 5 | 1 | 1 | 2 |
| 6067 | LN908554 | Candidatus Nitrosopelagicus brevis strain CN25 (CP007026.1) | 95.0 | 0 | 1 | 1 | 0 | 0 | 0 |
| 6070 | LN908555 | Candidatus Nitrosopelagicus brevis strain CN25 (CP007026.1) | 94.0 | 0 | 1 | 3 | 0 | 0 | 0 |
| 6124 | LN908556 | Candidatus Nitrosopumilus sp. NF5 (CP011070.1) and Candidatus Nitrosopumilus sp. D3C (CP010868.1) | 89.0 | 0 | 1 | 0 | 0 | 0 | 0 |
| 6134 | LN908557 | Candidatus Nitrosopumilus sp. HCA1 (KF957663.1) | 97.0 | 0 | 1 | 0 | 0 | 0 | 0 |
| 6139 | LN908558 | Candidatus Nitrosopelagicus brevis strain CN25 (CP007026.1) | 96.0 | 0 | 1 | 162 | 0 | 0 | 0 |
| 6154 | LN908559 | Candidatus Nitrosopumilus sp. NF5 (CP011070.1) and Candidatus Nitrosopumilus sp. D3C (CP010868.1) | 97.0 | 1 | 2 | 731 | 1 | 0 | 0 |
| 6170 | LN908560 | Candidatus Nitrosopumilus sp. NF5 (CP011070.1) and Candidatus Nitrosopumilus sp. D3C (CP010868.1) | 96.0 | 0 | 1 | 9 | 0 | 0 | 0 |
| 6191 | LN908561 | Candidatus Nitrosopumilus sp. HCA1 (KF957663.1) | 99.0 | 0 | 4 | 40 | 0 | 0 | 1 |
| 6212 | LN908562 | Candidatus Nitrosopumilus sp. HCA1 (KF957663.1) | 99.0 | 0 | 1 | 0 | 0 | 0 | 0 |
| 6246 | LN908563 | Candidatus Nitrosopelagicus brevis strain CN25 (CP007026.1) | 93.0 | 0 | 1 | 0 | 0 | 0 | 0 |
| 6262 | LN908564 | Candidatus Nitrosopelagicus brevis strain CN25 (CP007026.1) | 91.0 | 0 | 1 | 0 | 0 | 0 | 0 |
| 6265 | LN908565 | Candidatus Nitrosopelagicus brevis strain CN25 (CP007026.1) | 98.0 | 0 | 2 | 50 | 0 | 0 | 0 |
| 6267 | LN908566 | Candidatus Nitrosopelagicus brevis strain CN25 (CP007026.1) | 96.0 | 0 | 2 | 479 | 0 | 0 | 0 |
| 6307 | LN908567 | *Nitrosopumilus maritimus* strain SCM1 (NR_102913) | 95.0 | 0 | 1 | 1 | 0 | 0 | 0 |
| 6317 | LN908568 | Candidatus Nitrosopelagicus brevis strain CN25 (CP007026.1) | 96.0 | 0 | 1 | 9 | 0 | 0 | 0 |
| 6345 | LN908569 | Candidatus Nitrosopelagicus brevis strain CN25 (CP007026.1) | 91.0 | 0 | 1 | 0 | 0 | 0 | 0 |
| 6352 | LN908570 | Candidatus Nitrosopumilus sp. NF5 (CP011070.1) and Candidatus Nitrosopumilus sp. D3C (CP010868.1) | 97.0 | 0 | 1 | 1 | 0 | 0 | 0 |
| 6368 | LN908571 | Candidatus Nitrosopelagicus brevis strain CN25 (CP007026.1) | 98.0 | 2 | 1 | 29 | 0 | 0 | 1 |
| 6372 | LN908572 | Candidatus Nitrosopumilus sp. HCA1 (KF957663.1) | 99.0 | 0 | 1 | 2 | 0 | 0 | 0 |
| 6382 | LN908573 | Candidatus Nitrosopumilus sp. HCA1 (KF957663.1) | 91.0 | 0 | 1 | 2 | 0 | 0 | 0 |
| 6416 | LN908574 | Candidatus Nitrosopelagicus brevis strain CN25 (CP007026.1) | 97.0 | 1 | 1 | 2 | 0 | 0 | 3 |
| 6442 | LN908575 | Candidatus Nitrosopelagicus brevis strain CN25 (CP007026.1) | 96.0 | 3 | 1 | 176 | 0 | 0 | 2 |
| 6447 | LN908576 | Candidatus Nitrosopelagicus brevis strain CN25 (CP007026.1) | 89.0 | 0 | 1 | 0 | 0 | 0 | 0 |
| 6492 | LN908577 | Candidatus Nitrosopumilus sp. NF5 (CP011070.1) and Candidatus Nitrosopumilus sp. D3C (CP010868.1) | 97.0 | 0 | 1 | 6 | 0 | 0 | 0 |
| 6494 | LN908578 | Candidatus Nitrosopumilus sp. NF5 (CP011070.1) and Candidatus Nitrosopumilus sp. D3C (CP010868.1) | 92.0 | 1 | 1 | 1 | 0 | 0 | 0 |
| 6535 | LN908579 | Candidatus Nitrosopelagicus brevis strain CN25 (CP007026.1) | 95.0 | 4 | 0 | 471 | 3 | 0 | 0 |
| 6547 | LN908580 | Candidatus Nitrosopelagicus brevis strain CN25 (CP007026.1) | 96.0 | 2 | 0 | 151 | 0 | 0 | 0 |
| 6551 | LN908581 | Candidatus Nitrosopumilus sp. HCA1 (KF957663.1) | 98.0 | 2 | 0 | 41 | 0 | 0 | 0 |
| 6568 | LN908582 | Candidatus Nitrosopumilus sp. NF5 (CP011070.1) and Candidatus Nitrosopumilus sp. D3C (CP010868.1) | 93.0 | 0 | 0 | 1 | 0 | 0 | 0 |
| 6575 | LN908583 | Candidatus Nitrosopelagicus brevis strain CN25 (CP007026.1) | 92.0 | 0 | 0 | 1 | 0 | 0 | 0 |
| 6584 | LN908584 | Candidatus Nitrosopelagicus brevis strain CN25 (CP007026.1) | 96.0 | 0 | 0 | 318 | 0 | 0 | 0 |
| 6612 | LN908585 | Candidatus Nitrosopumilus sp. NF5 (CP011070.1) and Candidatus Nitrosopumilus sp. D3C (CP010868.1) | 98.0 | 0 | 0 | 20 | 0 | 0 | 0 |
| 6629 | LN908586 | Candidatus Nitrosopelagicus brevis strain CN25 (CP007026.1) | 97.0 | 2 | 0 | 695 | 4 | 3 | 1 |
| 6657 | LN908587 | Candidatus Nitrosopumilus sp. HCA1 (KF957663.1) | 98.0 | 23 | 5 | 1001 | 8 | 13 | 15 |
| 6660 | LN908588 | Candidatus Nitrosopelagicus brevis strain CN25 (CP007026.1) | 91.0 | 0 | 0 | 2 | 0 | 0 | 0 |
| 6682 | LN908589 | Candidatus Nitrosopelagicus brevis strain CN25 (CP007026.1) | 94.0 | 6 | 0 | 241 | 0 | 0 | 2 |
| 6725 | LN908590 | Candidatus Nitrosopelagicus brevis strain CN25 (CP007026.1) | 96.0 | 5 | 0 | 220 | 0 | 1 | 0 |
| 6741 | LN908591 | Candidatus Nitrosopelagicus brevis strain CN25 (CP007026.1) | 95.0 | 4 | 0 | 213 | 0 | 0 | 0 |
| 6755 | LN908592 | Candidatus Nitrosopumilus sp. NF5 (CP011070.1) and Candidatus Nitrosopumilus sp. D3C (CP010868.1) | 93.0 | 0 | 0 | 2 | 0 | 0 | 0 |
| 6784 | LN908593 | Candidatus Nitrosopelagicus brevis strain CN25 (CP007026.1) | 95.0 | 0 | 0 | 1 | 0 | 0 | 0 |
| 6817 | LN908594 | Candidatus Nitrosopelagicus brevis strain CN25 (CP007026.1) | 97.0 | 0 | 0 | 3 | 0 | 0 | 0 |
| 6832 | LN908595 | Candidatus Nitrosopelagicus brevis strain CN25 (CP007026.1) | 92.0 | 0 | 0 | 1 | 0 | 0 | 0 |
| 6842 | LN908596 | Candidatus Nitrosopelagicus brevis strain CN25 (CP007026.1) | 96.0 | 1 | 0 | 2725 | 1 | 1 | 0 |
| 6853 | LN908597 | Candidatus Nitrosopelagicus brevis strain CN25 (CP007026.1) | 96.0 | 0 | 0 | 266 | 0 | 0 | 0 |
| 6860 | LN908598 | Candidatus Nitrosopumilus sp. NF5 (CP011070.1) and Candidatus Nitrosopumilus sp. D3C (CP010868.1) | 98.0 | 3 | 0 | 134 | 0 | 0 | 0 |
| 6876 | LN908599 | Candidatus Nitrosopelagicus brevis strain CN25 (CP007026.1) | 96.0 | 2 | 0 | 3049 | 1 | 0 | 1 |
| 6882 | LN908600 | Candidatus Nitrosopelagicus brevis strain CN25 (CP007026.1) | 96.0 | 0 | 0 | 1049 | 0 | 0 | 0 |
| 6904 | LN908601 | Candidatus Nitrosopumilus sp. HCA1 (KF957663.1) | 92.0 | 0 | 0 | 1 | 0 | 0 | 0 |
| 6908 | LN908602 | Candidatus Nitrosopelagicus brevis strain CN25 (CP007026.1) | 96.0 | 0 | 0 | 13 | 0 | 0 | 0 |
| 6909 | LN908603 | Candidatus Nitrosopelagicus brevis strain CN25 (CP007026.1) | 96.0 | 0 | 0 | 2 | 0 | 0 | 0 |
| 6917 | LN908604 | Candidatus Nitrosopumilus sp. NF5 (CP011070.1) and Candidatus Nitrosopumilus sp. D3C (CP010868.1) | 96.0 | 1 | 0 | 16 | 0 | 0 | 0 |
| 6933 | LN908605 | Candidatus Nitrosopumilus sp. HCA1 (KF957663.1) | 100.0 | 3 | 0 | 677 | 6 | 6 | 26 |
| 6940 | LN908606 | Candidatus Nitrosopelagicus brevis strain CN25 (CP007026.1) | 92.0 | 0 | 0 | 2 | 0 | 0 | 0 |
| 6968 | LN908607 | Candidatus Nitrosopumilus sp. NF5 (CP011070.1) and Candidatus Nitrosopumilus sp. D3C (CP010868.1) | 95.0 | 0 | 0 | 204 | 0 | 1 | 0 |
| 6973 | LN908608 | Candidatus Nitrosopelagicus brevis strain CN25 (CP007026.1) | 88.0 | 0 | 0 | 1 | 0 | 0 | 0 |
| 6982 | LN908609 | Candidatus Nitrosopelagicus brevis strain CN25 (CP007026.1) | 96.0 | 2 | 0 | 547 | 0 | 0 | 0 |
| 7004 | LN908610 | Candidatus Nitrosopumilus sp. HCA1 (KF957663.1) | 99.0 | 3 | 0 | 65 | 1 | 0 | 1 |
| 7043 | LN908611 | Candidatus Nitrosopelagicus brevis strain CN25 (CP007026.1) | 100.0 | 3 | 0 | 167 | 1 | 3 | 9 |
| 7047 | LN908612 | Candidatus Nitrosopumilus sp. NF5 (CP011070.1) and Candidatus Nitrosopumilus sp. D3C (CP010868.1) | 96.0 | 0 | 0 | 19 | 0 | 0 | 0 |
| 7068 | LN908613 | Candidatus Nitrosopumilus sp. NF5 (CP011070.1) and Candidatus Nitrosopumilus sp. D3C (CP010868.1) | 95.0 | 0 | 0 | 2 | 0 | 0 | 0 |
| 7085 | LN908614 | Candidatus Nitrosopumilus sp. NF5 (CP011070.1) and Candidatus Nitrosopumilus sp. D3C (CP010868.1) | 98.0 | 0 | 0 | 4 | 0 | 0 | 0 |
| 7090 | LN908615 | Candidatus Nitrosopumilus sp. NF5 (CP011070.1) and Candidatus Nitrosopumilus sp. D3C (CP010868.1) | 92.0 | 0 | 0 | 1 | 0 | 0 | 0 |
| 7094 | LN908616 | Candidatus Nitrosopelagicus brevis strain CN25 (CP007026.1) | 95.0 | 0 | 0 | 326 | 0 | 0 | 0 |
| 7097 | LN908617 | Candidatus Nitrosopelagicus brevis strain CN25 (CP007026.1) | 95.0 | 0 | 0 | 516 | 1 | 0 | 0 |
| 7099 | LN908618 | Candidatus Nitrosopelagicus brevis strain CN25 (CP007026.1) | 98.0 | 0 | 0 | 101 | 0 | 0 | 0 |
| 7103 | LN908619 | Candidatus Nitrosopelagicus brevis strain CN25 (CP007026.1) | 96.0 | 1 | 0 | 210 | 0 | 0 | 0 |
| 7122 | LN908620 | Candidatus Nitrosopelagicus brevis strain CN25 (CP007026.1) | 92.0 | 0 | 0 | 1 | 0 | 0 | 0 |
| 7127 | LN908621 | Candidatus Nitrosopumilus sp. NF5 (CP011070.1) and Candidatus Nitrosopumilus sp. D3C (CP010868.1) | 86.0 | 0 | 0 | 1 | 0 | 0 | 0 |
| 7133 | LN908622 | Candidatus Nitrosopelagicus brevis strain CN25 (CP007026.1) | 98.0 | 1 | 1 | 1531 | 0 | 0 | 0 |
| 7159 | LN908623 | Candidatus Nitrosopelagicus brevis strain CN25 (CP007026.1) | 92.0 | 0 | 0 | 1 | 0 | 0 | 0 |
| 7179 | LN908624 | Candidatus Nitrosopumilus sp. NF5 (CP011070.1) and Candidatus Nitrosopumilus sp. D3C (CP010868.1) | 94.0 | 0 | 0 | 1 | 0 | 0 | 0 |
| 7186 | LN908625 | Candidatus Nitrosopumilus sp. NF5 (CP011070.1) and Candidatus Nitrosopumilus sp. D3C (CP010868.1) | 97.0 | 17 | 6 | 9 | 0 | 0 | 1 |
| 7218 | LN908626 | Candidatus Nitrosopelagicus brevis strain CN25 (CP007026.1) | 94.0 | 0 | 0 | 3 | 0 | 0 | 0 |
| 7241 | LN908627 | Candidatus Nitrosopelagicus brevis strain CN25 (CP007026.1) | 88.0 | 0 | 0 | 1 | 0 | 0 | 0 |
| 7249 | LN908628 | Candidatus Nitrosopelagicus brevis strain CN25 (CP007026.1) | 95.0 | 2 | 0 | 2 | 1 | 0 | 2 |
| 7256 | LN908629 | Candidatus Nitrosopumilus sp. HCA1 (KF957663.1) | 94.0 | 0 | 0 | 1 | 0 | 0 | 0 |
| 7259 | LN908630 | Candidatus Nitrosopelagicus brevis strain CN25 (CP007026.1) | 95.0 | 0 | 0 | 2 | 0 | 0 | 0 |
| 7262 | LN908631 | Candidatus Nitrosopumilus sp. NF5 (CP011070.1) and Candidatus Nitrosopumilus sp. D3C (CP010868.1) | 95.0 | 0 | 0 | 3 | 0 | 0 | 0 |
| 7265 | LN908632 | Candidatus Nitrosopumilus sp. NF5 (CP011070.1) and Candidatus Nitrosopumilus sp. D3C (CP010868.1) | 94.0 | 0 | 0 | 2 | 0 | 0 | 0 |
| 7290 | LN908633 | Candidatus Nitrosopelagicus brevis strain CN25 (CP007026.1) | 90.0 | 0 | 0 | 1 | 0 | 0 | 0 |
| 7306 | LN908634 | Candidatus Nitrosopumilus sp. NF5 (CP011070.1) and Candidatus Nitrosopumilus sp. D3C (CP010868.1) | 96.0 | 0 | 0 | 1 | 0 | 0 | 0 |
| 7324 | LN908635 | Candidatus Nitrosopumilus sp. NF5 (CP011070.1) and Candidatus Nitrosopumilus sp. D3C (CP010868.1) | 95.0 | 7 | 2 | 7 | 1 | 0 | 0 |
| 7333 | LN908636 | Candidatus Nitrosopumilus sp. NF5 (CP011070.1) and Candidatus Nitrosopumilus sp. D3C (CP010868.1) | 94.0 | 0 | 0 | 2 | 0 | 0 | 0 |
| 7376 | LN908637 | Candidatus Nitrosopelagicus brevis strain CN25 (CP007026.1) | 96.0 | 2 | 0 | 1 | 0 | 0 | 0 |
| 7397 | LN908638 | Candidatus Nitrosopelagicus brevis strain CN25 (CP007026.1) | 96.0 | 4 | 0 | 3 | 1 | 1 | 0 |
| 7432 | LN908639 | Candidatus Nitrosopelagicus brevis strain CN25 (CP007026.1) | 96.0 | 0 | 0 | 1 | 0 | 0 | 0 |
| 7436 | LN908640 | Candidatus Nitrosopelagicus brevis strain CN25 (CP007026.1) | 95.0 | 0 | 0 | 1 | 0 | 0 | 0 |
| 7458 | LN908641 | Candidatus Nitrosopumilus sp. NF5 (CP011070.1) and Candidatus Nitrosopumilus sp. D3C (CP010868.1) | 93.0 | 0 | 0 | 1 | 0 | 0 | 0 |
| 7477 | LN908642 | Candidatus Nitrosopelagicus brevis strain CN25 (CP007026.1) | 95.0 | 19 | 1 | 10 | 1 | 3 | 2 |
| 7491 | LN908643 | Candidatus Nitrosopumilus sp. NF5 (CP011070.1) and Candidatus Nitrosopumilus sp. D3C (CP010868.1) | 96.0 | 0 | 0 | 2 | 0 | 0 | 0 |
| 7514 | LN908644 | Candidatus Nitrosopelagicus brevis strain CN25 (CP007026.1) | 96.0 | 14 | 2 | 16 | 1 | 0 | 0 |
| 7585 | LN908645 | Candidatus Nitrosopelagicus brevis strain CN25 (CP007026.1) | 96.0 | 0 | 0 | 1 | 0 | 0 | 0 |
| 7617 | LN908646 | Candidatus Nitrosopumilus sp. NF5 (CP011070.1) and Candidatus Nitrosopumilus sp. D3C (CP010868.1) | 96.0 | 36 | 2 | 28 | 5 | 2 | 2 |
| 7618 | LN908647 | Candidatus Nitrosopelagicus brevis strain CN25 (CP007026.1) | 96.0 | 5 | 1 | 6 | 1 | 0 | 0 |
| 7623 | LN908648 | Candidatus Nitrosopelagicus brevis strain CN25 (CP007026.1) | 91.0 | 0 | 0 | 1 | 0 | 0 | 0 |
| 7632 | LN908649 | Candidatus Nitrosopumilus sp. NF5 (CP011070.1) and Candidatus Nitrosopumilus sp. D3C (CP010868.1) | 94.0 | 0 | 0 | 1 | 0 | 0 | 0 |
| 7672 | LN908650 | Candidatus Nitrosopelagicus brevis strain CN25 (CP007026.1) | 89.0 | 0 | 0 | 1 | 0 | 0 | 0 |
| 7728 | LN908651 | Candidatus Nitrosopumilus sp. NF5 (CP011070.1) and Candidatus Nitrosopumilus sp. D3C (CP010868.1) | 95.0 | 0 | 0 | 1 | 0 | 0 | 0 |
| 7731 | LN908652 | Candidatus Nitrosopumilus sp. NF5 (CP011070.1) and Candidatus Nitrosopumilus sp. D3C (CP010868.1) | 92.0 | 0 | 0 | 2 | 0 | 0 | 0 |
| 7732 | LN908653 | Candidatus Nitrosopelagicus brevis strain CN25 (CP007026.1) | 95.0 | 14 | 0 | 7 | 1 | 0 | 4 |
| 7735 | LN908654 | Candidatus Nitrosopumilus sp. NF5 (CP011070.1) and Candidatus Nitrosopumilus sp. D3C (CP010868.1) | 93.0 | 0 | 1 | 1 | 0 | 0 | 0 |
| 7743 | LN908655 | Candidatus Nitrosopelagicus brevis strain CN25 (CP007026.1) | 95.0 | 12 | 1 | 10 | 1 | 0 | 3 |
| 7752 | LN908656 | Candidatus Nitrosopelagicus brevis strain CN25 (CP007026.1) | 96.0 | 0 | 0 | 2 | 0 | 0 | 0 |
| 7786 | LN908657 | Candidatus Nitrosopelagicus brevis strain CN25 (CP007026.1) | 95.0 | 0 | 0 | 1 | 0 | 0 | 0 |
| 7789 | LN908658 | Candidatus Nitrosopelagicus brevis strain CN25 (CP007026.1) | 94.0 | 0 | 0 | 2 | 0 | 0 | 0 |
| 7806 | LN908659 | Candidatus Nitrosopumilus sp. NF5 (CP011070.1) and Candidatus Nitrosopumilus sp. D3C (CP010868.1) | 92.0 | 0 | 0 | 1 | 0 | 0 | 0 |
| 7835 | LN908660 | Candidatus Nitrosopelagicus brevis strain CN25 (CP007026.1) | 97.0 | 2 | 0 | 2 | 0 | 0 | 0 |
| 7844 | LN908661 | Candidatus Nitrosopelagicus brevis strain CN25 (CP007026.1) | 96.0 | 40 | 4 | 30 | 3 | 1 | 1 |
| 7851 | LN908662 | Candidatus Nitrosopelagicus brevis strain CN25 (CP007026.1) | 96.0 | 1 | 0 | 1 | 1 | 0 | 0 |
| 7862 | LN908663 | Candidatus Nitrosopelagicus brevis strain CN25 (CP007026.1) | 91.0 | 0 | 0 | 1 | 0 | 0 | 0 |
| 7898 | LN908664 | Candidatus Nitrosopumilus sp. NF5 (CP011070.1) and Candidatus Nitrosopumilus sp. D3C (CP010868.1) | 93.0 | 0 | 0 | 1 | 0 | 0 | 0 |
| 7901 | LN908665 | Candidatus Nitrosopumilus sp. NF5 (CP011070.1) and Candidatus Nitrosopumilus sp. D3C (CP010868.1) | 94.0 | 0 | 0 | 1 | 0 | 0 | 0 |
| 7903 | LN908666 | Candidatus Nitrosopelagicus brevis strain CN25 (CP007026.1) | 96.0 | 12 | 0 | 11 | 0 | 1 | 1 |
| 7905 | LN908667 | Candidatus Nitrosopumilus sp. NF5 (CP011070.1) and Candidatus Nitrosopumilus sp. D3C (CP010868.1) | 91.0 | 0 | 0 | 1 | 0 | 0 | 0 |
| 7934 | LN908668 | Candidatus Nitrosopelagicus brevis strain CN25 (CP007026.1) | 96.0 | 1 | 0 | 1 | 0 | 0 | 0 |
| 7959 | LN908669 | Candidatus Nitrosopelagicus brevis strain CN25 (CP007026.1) | 96.0 | 11 | 2 | 16 | 0 | 0 | 1 |
| 7975 | LN908670 | Candidatus Nitrosopelagicus brevis strain CN25 (CP007026.1) | 96.0 | 20 | 3 | 21 | 0 | 0 | 0 |
| 7976 | LN908671 | Candidatus Nitrosopelagicus brevis strain CN25 (CP007026.1) | 96.0 | 3 | 0 | 2 | 0 | 0 | 1 |
| 7997 | LN908672 | Candidatus Nitrosopelagicus brevis strain CN25 (CP007026.1) | 96.0 | 7 | 0 | 7 | 1 | 0 | 1 |
| 8002 | LN908673 | Candidatus Nitrosopumilus sp. NF5 (CP011070.1) and Candidatus Nitrosopumilus sp. D3C (CP010868.1) | 96.0 | 11 | 2 | 6 | 0 | 2 | 1 |
| 8003 | LN908674 | Candidatus Nitrosopelagicus brevis strain CN25 (CP007026.1) | 92.0 | 0 | 1 | 1 | 0 | 0 | 0 |
| 8016 | LN908675 | Candidatus Nitrosopelagicus brevis strain CN25 (CP007026.1) | 96.0 | 28 | 2 | 16 | 3 | 0 | 2 |
| 8019 | LN908676 | Candidatus Nitrosopumilus sp. NF5 (CP011070.1) and Candidatus Nitrosopumilus sp. D3C (CP010868.1) | 96.0 | 1 | 0 | 3 | 0 | 0 | 0 |
| 8023 | LN908677 | Candidatus Nitrosopelagicus brevis strain CN25 (CP007026.1) | 96.0 | 12 | 0 | 2 | 0 | 0 | 0 |
| 8078 | LN908678 | Candidatus Nitrosopumilus sp. NF5 (CP011070.1) and Candidatus Nitrosopumilus sp. D3C (CP010868.1) | 89.0 | 0 | 0 | 1 | 0 | 0 | 0 |
| 8099 | LN908679 | Candidatus Nitrosopumilus sp. NF5 (CP011070.1) and Candidatus Nitrosopumilus sp. D3C (CP010868.1) | 86.0 | 0 | 0 | 1 | 0 | 0 | 0 |
| 8105 | LN908680 | Candidatus Nitrosopelagicus brevis strain CN25 (CP007026.1) | 96.0 | 0 | 0 | 1 | 0 | 0 | 0 |
| 8134 | LN908681 | Candidatus Nitrosopelagicus brevis strain CN25 (CP007026.1) | 96.0 | 0 | 0 | 1 | 0 | 0 | 0 |
| 8198 | LN908682 | Candidatus Nitrosopelagicus brevis strain CN25 (CP007026.1) | 96.0 | 0 | 0 | 2 | 0 | 0 | 0 |
| 8225 | LN908683 | Candidatus Nitrosopelagicus brevis strain CN25 (CP007026.1) | 96.0 | 17 | 2 | 17 | 1 | 0 | 2 |
| 8268 | LN908684 | Candidatus Nitrosopumilus sp. PS0 (KF957664.1) | 96.0 | 0 | 0 | 1 | 0 | 0 | 0 |
| 8274 | LN908685 | Candidatus Nitrosopelagicus brevis strain CN25 (CP007026.1) | 89.0 | 0 | 0 | 1 | 0 | 0 | 0 |
| 8294 | LN908686 | Candidatus Nitrosopumilus sp. NF5 (CP011070.1) and Candidatus Nitrosopumilus sp. D3C (CP010868.1) | 94.0 | 0 | 0 | 1 | 0 | 0 | 0 |
| 8303 | LN908687 | Candidatus Nitrosopumilus sp. NF5 (CP011070.1) and Candidatus Nitrosopumilus sp. D3C (CP010868.1) | 98.0 | 27 | 1 | 9 | 5 | 7 | 14 |
| 8359 | LN908688 | Candidatus Nitrosopelagicus brevis strain CN25 (CP007026.1) | 91.0 | 0 | 0 | 1 | 0 | 0 | 0 |
| 8361 | LN908689 | Candidatus Nitrosopelagicus brevis strain CN25 (CP007026.1) | 98.0 | 38 | 3 | 25 | 6 | 9 | 25 |
| 8364 | LN908690 | Candidatus Nitrosopumilus sp. NF5 (CP011070.1) and Candidatus Nitrosopumilus sp. D3C (CP010868.1) | 96.0 | 27 | 0 | 13 | 1 | 0 | 0 |
| 8367 | LN908691 | Candidatus Nitrosopumilus sp. NF5 (CP011070.1) and Candidatus Nitrosopumilus sp. D3C (CP010868.1) | 94.0 | 5 | 0 | 6 | 0 | 0 | 0 |
| 10655 | LN908692 | Candidatus Nitrosopumilus sp. HCA1 (KF957663.1) | 98.0 | 6 | 4 | 6 | 382 | 113 | 360 |
| 10815 | LN908693 | Candidatus Nitrosopelagicus brevis strain CN25 (CP007026.1) | 94.0 | 0 | 0 | 0 | 3 | 0 | 0 |
| 12986 | LN908694 | Candidatus Nitrosopumilus sp. NF5 (CP011070.1) and Candidatus Nitrosopumilus sp. D3C (CP010868.1) | 89.0 | 0 | 0 | 0 | 1 | 0 | 0 |
| 13116 | LN908695 | Candidatus Nitrosopumilus sp. HCA1 (KF957663.1) | 89.0 | 0 | 0 | 0 | 1 | 0 | 0 |
| 15118 | LN908696 | Candidatus Nitrosopumilus sp. NF5 (CP011070.1) and Candidatus Nitrosopumilus sp. D3C (CP010868.1) | 90.0 | 0 | 0 | 0 | 0 | 1 | 0 |
| 15582 | LN908697 | Candidatus Nitrosopumilus sp. HCA1 (KF957663.1) | 92.0 | 0 | 0 | 0 | 0 | 2 | 0 |
| 15592 | LN908698 | Candidatus Nitrosopelagicus brevis strain CN25 (CP007026.1) | 99.0 | 0 | 0 | 2 | 0 | 1 | 2 |
| 15622 | LN908699 | Candidatus Nitrosopelagicus brevis strain CN25 (CP007026.1) | 94.0 | 0 | 0 | 0 | 0 | 1 | 0 |
| 15796 | LN908700 | Candidatus Nitrososphaera evergladensis SR1 (CP007174.1) | 94.0 | 0 | 0 | 0 | 0 | 1 | 0 |
| 16003 | LN908701 | Candidatus Nitrosopumilus sp. HCA1 (KF957663.1) | 92.0 | 0 | 0 | 0 | 0 | 1 | 0 |
| 16021 | LN908702 | Candidatus Nitrosopelagicus brevis strain CN25 (CP007026.1) | 97.0 | 7 | 0 | 6 | 0 | 2 | 4 |
| 16053 | LN908703 | Candidatus Nitrosopumilus sp. HCA1 (KF957663.1) | 99.0 | 0 | 1 | 3 | 0 | 1 | 11 |
| 16152 | LN908704 | Candidatus Nitrosopumilus sp. HCA1 (KF957663.1) | 96.0 | 0 | 0 | 0 | 0 | 1 | 3 |
| 16307 | LN908705 | Candidatus Nitrosopumilus sp. HCA1 (KF957663.1) | 99.0 | 0 | 1 | 0 | 0 | 1 | 6 |
| 16911 | LN908706 | Candidatus Nitrosopumilus sp. NF5 (CP011070.1) and Candidatus Nitrosopumilus sp. D3C (CP010868.1) | 91.0 | 0 | 0 | 0 | 0 | 0 | 1 |
| 17259 | LN908707 | Candidatus Nitrosopumilus sp. NF5 (CP011070.1) and Candidatus Nitrosopumilus sp. D3C (CP010868.1) | 90.0 | 0 | 0 | 0 | 0 | 0 | 1 |
| 18331 | LN908708 | Candidatus Nitrosopumilus sp. HCA1 (KF957663.1) | 93.0 | 0 | 0 | 0 | 0 | 0 | 1 |
| 18360 | LN908709 | *Nitrososphaera viennensis* EN76 (CP007536.1) | 97.0 | 0 | 0 | 0 | 0 | 0 | 1 |
| 18962 | LN908710 | Candidatus Nitrosopumilus sp. HCA1 (KF957663.1) | 91.0 | 0 | 0 | 0 | 0 | 0 | 1 |
| 19169 | LN908711 | *Nitrososphaera viennensis* EN76 (CP007536.1) | 95.0 | 0 | 0 | 0 | 0 | 0 | 1 |
| 19401 | LN908712 | Candidatus Nitrosopelagicus brevis strain CN25 (CP007026.1) | 94.0 | 1 | 0 | 0 | 0 | 0 | 0 |
| 19456 | LN908713 | Candidatus Nitrosopumilus sp. HCA1 (KF957663.1) | 94.0 | 1 | 0 | 0 | 0 | 0 | 0 |
| 19555 | LN908714 | Candidatus Nitrosopumilus sp. NF5 (CP011070.1) and Candidatus Nitrosopumilus sp. D3C (CP010868.1) | 92.0 | 0 | 0 | 1 | 0 | 0 | 0 |
| 19566 | LN908715 | Candidatus Nitrosopelagicus brevis strain CN25 (CP007026.1) | 97.0 | 0 | 0 | 2 | 0 | 0 | 0 |
| 19604 | LN908716 | Candidatus Nitrosopumilus sp. NF5 (CP011070.1) and Candidatus Nitrosopumilus sp. D3C (CP010868.1) | 96.0 | 0 | 0 | 1 | 0 | 0 | 0 |
| 19694 | LN908717 | Candidatus Nitrosopelagicus brevis strain CN25 (CP007026.1) | 94.0 | 0 | 0 | 0 | 1 | 0 | 0 |
| 19802 | LN908718 | Candidatus Nitrosopumilus sp. HCA1 (KF957663.1) | 97.0 | 0 | 0 | 0 | 0 | 0 | 1 |
| 19838 | LN908719 | Candidatus Nitrosopelagicus brevis strain CN25 (CP007026.1) | 96.0 | 0 | 0 | 1 | 0 | 0 | 0 |
| 19839 | LN908720 | Candidatus Nitrosopumilus sp. NF5 (CP011070.1) and Candidatus Nitrosopumilus sp. D3C (CP010868.1) | 95.0 | 0 | 0 | 1 | 0 | 0 | 0 |

**Supplement References**

Kaiser, J., Rockmann, T., & Brenninkmeijer, C. A. M. (2003). Complete and accurate mass spectrometric isotope analysis of tropospheric nitrous oxide. *Journal of Geophysical Research*, *108*(D15), 1–17. http://doi.org/10.1029/2003JD003613

Sigman, D. M., Casciotti, K. L., Andreani, M., Barford, C., Galanter, M., & Bohlke, J. K. (2001). A bacterial method for the nitrogen isotopic analysis of nitrate in seawater and freshwater. *Analytical Chemistry*, *73*, 4145–4153.
